# Fast-SG: an alignment-free algorithm for hybrid assembly

**DOI:** 10.1093/gigascience/giy048

**Published:** 2018-05-05

**Authors:** Alex Di Genova, Gonzalo A Ruz, Marie-France Sagot, Alejandro Maass

**Affiliations:** 1 Facultad de Ingeniería y Ciencias, Universidad Adolfo Ibáñez, Santiago, Chile; 2 Mathomics Bioinformatics Laboratory, Center for Mathematical Modeling, University of Chile, Av. Beauchef 851., 7th floor, Santiago, Chile; 3 Inria Grenoble Rhon̂e-Alpes, 655, Avenue de l’Europe, 38334 Montbonnot, France; 4 CNRS, UMR5558, Université Claude Bernard Lyon 1, 43, Boulevard du 11 Novembre 1918, 69622 Villeurbanne, France; 5 Fondap Center for Genome Regulation, Av. Blanco Encalada 2085, 3rd floor, Santiago, Chile; 6 Center of Applied Ecology and Sustainability (CAPES), Santiago, Chile; 7 Department of Mathematical Engineering, University of Chile, Av. Beauchef 851., 5th floor, Santiago, Chile

**Keywords:** hybrid assembly, genome scaffolding, alignment-free

## Abstract

**Background:**

Long-read sequencing technologies are the ultimate solution for genome repeats, allowing near reference-level reconstructions of large genomes. However, long-read *de novo* assembly pipelines are computationally intense and require a considerable amount of coverage, thereby hindering their broad application to the assembly of large genomes. Alternatively, hybrid assembly methods that combine short- and long-read sequencing technologies can reduce the time and cost required to produce *de novo* assemblies of large genomes.

**Results:**

Here, we propose a new method, called Fast-SG, that uses a new ultrafast alignment-free algorithm specifically designed for constructing a scaffolding graph using light-weight data structures. Fast-SG can construct the graph from either short or long reads. This allows the reuse of efficient algorithms designed for short-read data and permits the definition of novel modular hybrid assembly pipelines. Using comprehensive standard datasets and benchmarks, we show how Fast-SG outperforms the state-of-the-art short-read aligners when building the scaffoldinggraph and can be used to extract linking information from either raw or error-corrected long reads. We also show how a hybrid assembly approach using Fast-SG with shallow long-read coverage (5X) and moderate computational resources can produce long-range and accurate reconstructions of the genomes of *Arabidopsis thaliana* (Ler-0) and human (NA12878).

**Conclusions:**

Fast-SG opens a door to achieve accurate hybrid long-range reconstructions of large genomes with low effort, high portability, and low cost.

## Findings

### Background

The major challenge of whole genome *de novo* assembly is to solve repeats [1, 2]. These correspond to nearly identical genomic sequences that occur at multiple locations in a genome. To address this challenge, two major types of approaches have been proposed, one using paired short reads [3] and the other long reads [4].

In the second case, the aim is to entirely capture the repeats within the long reads. The nonrepeated suffix and prefix sequences of such long reads are used to compute unique overlaps, which then make it possible to unambiguously expand the original reads into larger ones, called contigs, in a process that may sometimes (but not always) directly lead to inferring the entire genomic sequence.

The first type of approach needs to be associated to an operation called genome scaffolding. The short reads are still first assembled into contigs as above, either by computing overlaps [5] or by using de Bruijn graphs [6]. The contigs obtained in this case will, however, not span the whole genome. Indeed, most often they will be much shorter. They then need to be joined (i.e., linked together) in a second step. The linking information is in general provided by paired-end or mate-pair sequencing. Commonly, genomic fragments larger than 1 kb from which both ends are sequenced are denoted as mate-pair libraries, otherwise they are referred to in the literature as paired-end libraries. Genome scaffolding that uses paired short reads introduces gaps (i.e., unknown sequences) between the contigs, thereby once again not leading to the entire genomic sequence but to a set of so-called scaffold sequences, or scaffolds for short. A scaffold thus represents a set of ordered and oriented contigs.

The genome scaffolding problem was first formulated by Huson et al. [7]. The method proposed by the authors started by building what is called a scaffolding graph where the nodes represent the contigs and the edges encode the number of mate-pairs (weight), the orientation, and the distance between two different contigs. A greedy algorithm is then used to heuristically obtain optimal paths that will correspond to the scaffold sequences.

Most of the scaffolding methods that have been developed since Huson et al.'s formulation use the same type of graph, built with ultrafast short-read aligners [8-10] as a foundation for the scaffolding [3]. Algorithmic innovations in the area are mainly focused on how to select optimal paths (usually those of maximal weight) and thus obtain large and accurate scaffolds. Various approaches have been proposed based on dynamic programming [11], breadth-first search [12], maximum weight matching [13], or branch and bound [14], among others.

The new long-read sequencing technologies (Pacific Biosciences, Oxford Nanopore) suddenly changed the genome assembly scene by producing very long (>10 kb) reads that contain a high level of errors (on average 15% at the current time). These new technologies nevertheless extended the landscape of solvable repeat sequences [15]. Currently, *de novo* assemblers that use such long reads [4, 16] are thus able to finish bacterial genomes and to produce highly continuous reconstructions of human genomes [4, 17]. However, *de novo* assemblies of large genomes based on computing overlaps [5] are computationally intense [4] and require a considerable amount of coverage (50X) in order to error correct the inaccurate long-read sequences by self-correction methods, thereby hindering a broad application of these methods to the *de novo* assembly of large genomes [17].

*De novo* assemblies using long reads have nevertheless proven to be scalable to chromosomes [18, 19] when associated with complementary long-range information from novel library preparation techniques [20, 21]. Such new experimental libraries are sequenced on Illumina machines, leading to conventional paired-end reads. DOVETAIL genomics [20] thus produces useful linking information in the range of 1–200 kb, while 10X genomics [22], by using barcodes in a clever manner, produces linked-reads of up to 100 kb. Both technologies use long-range information within their assembly pipelines [20, 22] to build a scaffolding graph to which they apply their own algorithmic solutions to obtain the scaffold sequences. Both technologies were conceived with the aim of replacing the expensive and time-consuming experimental protocols required to produce long-range mate-pair libraries [23, 24] with short-read sequencing.

In principle, long-range information can be extracted directly from long reads in ranges restricted to the latter’s actual sizes. Such information can then be used to devise a hybrid assembly method, where high-quality contigs from short-read assemblies are used as nodes of the scaffolding graph, edges are created using linking information from the long reads, and the scaffolds are generated by a short-read scaffolder. However, there is currently a lack of algorithms for building a scaffolding graph from the long reads. Such an algorithm would allow the reuse of efficient existing short-read algorithms to compose novel hybrid assembly pipelines.

Being able to build such a graph from either short or long reads in an ultrafast way with moderate computational resources while keeping the structure standard enough to be compatible with the existing efficient short-read scaffolders are the main challenges that we address here. The method that we propose, Fast-SG, uses an alignment-free algorithm [25] strategy as well as information from varied sequence sources (Illumina, Pacific Biosciences, and Oxford Nanopore) and was conceived to maximize scalability, speed, and modularity. The latter characteristic, in particular, allows one to define novel hybrid assembly pipelines, which permits the efficient assembly of large genomes.

Fast-SG was extensively tested using a comprehensive set of standard datasets [3, 26] and benchmarks. We show that Fast-SG enables the hybrid assembly of large genomes and is especially effective with shallow long-read coverage data (5X–10X). Our hybrid strategy consists of the construction of several synthetic mate-pair libraries that could have an insert size up to Bacterial Artificial Chromosome (BACs,180 kb) and can be combined with a short-read scaffolder to generate long-range scaffolds. Such strategy scales to human-size genomes with moderate computational resources. Moreover, we show that Fast-SG is faster (7X–15X) than classic short-read aligners and is a powerful alternative for scaffolding with short mate-pair data.

We conclude by providing a procedure for an effective hybrid assembly with Fast-SG and we discuss how the strategy that we propose can be extended to use long reads to fill the gaps and error correct the scaffold sequences.

### Algorithm

#### Fast-SG index

The Fast-SG index consists of all the unique *k*-mers present in the set of target contigs at a given *k*-mer length. For each of them, we store the position, the strand and the contig of origin, using lightweight data structures such as Minimal Perfect Hashing [27] and Probabilistic Dictionary [28]. In the first step, we define the unique *k*-mers as being those with a frequency equal to 1 from the total set of distinct *k*-mers present in the target contig/genome sequences. To identify unique *k*-mers, we use Kmc3 [29], an ultrafast, parallel, and memory-frugal *k*-mer counter.

In the second step, each unique *k*-mer is hashed to the space of [2^0^, 2^64^] using a rolling hash function [30] and with hash values written on the fly to a binary file. Rolling hashing has the helpful property of computing hash values for consecutive *k*-mers in a sequence in $\mathcal {O}(k+l)$ time, where *k* is the *k*-mer length, *l* is the sequence length, and *k* < *l*. We use an efficient library implementation of rolling hash algorithms called Nthash [31], which implements a barrel shift function and a seed table of integers to compute hash values in both DNA strands faster.

In the third step, the static hash values stored in the binary file are used as input to create a minimal perfect hash function (henceforth denoted byMphf). Mphf provides a collision-free and space-saving way to store and look up hash values in constant worst-case access time for static sets. We use the library implementation provided by Limasset et al. [27], called Bbhash, which is simple, parallel, fast, and memory frugal. Moreover, it can store 10^10^ hash values using moderate computational resources (5Gb). The major feature ofMphf is its ability to map each key of *S* (in our case, the unique *k*-mer hashed values) to an integer in the interval [1, *N*] (injective function), with *N* = |*S*|, while avoiding the implicit storage of hash values by using cascade hash functions in conjunction with bit vectors. A significant parameter of Bbhash is the γ (gamma) factor. We use a γ factor equal to 4, which is an optimal value for fast query time, fast construction, and low memory usage [27]. When performing a query in the Mphf structure, it returns an index in the interval of [1, *N*], which has the same size as the static set *S*, allowing storage of related data for each *s* ∈ *S* using simple arrays. If we query a key not present in the initial static set *S*, Mphf could return a value in the interval [1, *N*] that is a false positive [28].

In the fourth step, to control the false-positive rate (*p*) of Mphf, we use a probabilistic set [28]. For each indexed element *s* ∈ *S* (unique *k*-mers), we store a fingerprint value using 16 bits in an array of size *N* = |*S*| at the corresponding Mphf index of *s*. The fingerprint is built by rehashing the hash value of *s* using the xor-shift hash function in the range [2^0^, 2^16^] and storing it in a bit-set array structure. We selected a fingerprint of size 16 bits because it has a low false-positive rate *p* = 1/2^16^ = 0.0000152.

Finally, we added the associated contig_id, strand and coordinate values of each unique *k*-mer stored in the Mphf and the probabilistic dictionary (Mphf-PD) by performing a single pass through the set of contigs/genome sequences using the same *k*-mer size. For each *k*-mer hit, we store the values (contig_id, coordinate and strand) in the index returned by the Mphf-PD structure using three vectors with the same size as the set *S*. After storing all the associated values, we end our index construction and return a reference to the new object. This object is the Fast-SG index. The memory required per *k*-mer is composed of 6 bits for the Mphf, 16 bits for the probabilistic dictionary, 32 bits for the contig_id, 32 bits for the coordinate, and 1 bit for the strand, adding to a total memory of 87 bits.

#### Fast-SG alignment-free method

The core of Fast-SG is an alignment-free algorithm specifically designed to construct the scaffolding graph from either short or long reads using lightweight data structures. Such graphs are built using as information the read pairs that map uniquely to different contigs. If the mappings are within an expected distance from one another given the respective orientation of the reads, an edge is added to the graph between the contigs [3]. The uniqueness property of the mapping is ensured by its high-quality score, which represents the confidence that the read indeed belongs to the reported genomic location [9, 10]. When a read belongs to two possible genomic locations, a score of 0 is commonly assigned.

**Figure 1:**
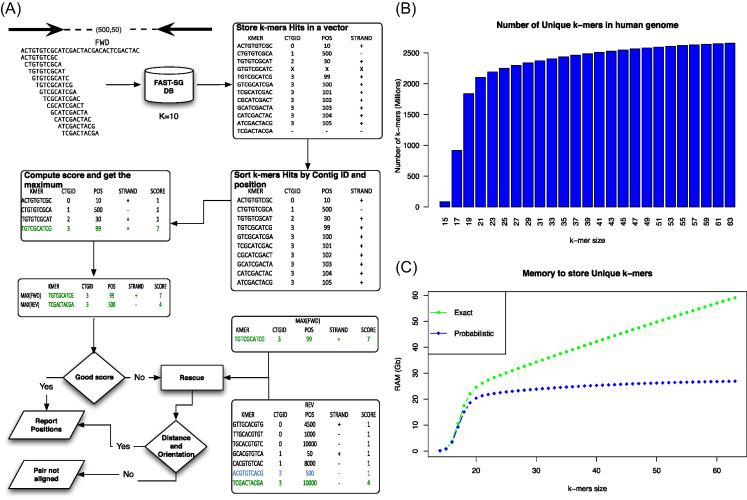
**(A)** Overview of the Fast-SG algorithm. **(B)** Number of unique *k*-mers (*y*-axis) in the human genome GRCh38.p10 as a function of the *k*-mer size (*x*-axis). **(C)** Memory required for indexing the unique *k*-mers of the human genome by Fast-SG and using an exact implementation. The blue dotted-line shows the memory required by Fast-SG as a function of the *k*-mer size. Green shows the memory required by an exact implementation that uses 2 bits per base. The amount of memory used by such implementation increases as a function of the *k*-mer size (*x*-axis). The memory of the index used in Fast-SG only increases with the number of *k*-mers to store.

Current short-read aligners identify the high-quality score mappings by indexing all the *k*-mers present in the set of contigs and using a seed-and-extend [9, 10] alignment approach. Instead, in Fast-SG, only the *k*-mers with a frequency equal to 1 are considered, and no alignment is performed. After building the Fast-SG index, the contig location for a pair of reads is determined following a number of steps as illustrated in Fig. 1A.

The first step performs look-ups of the *k*-mers of the forward (resp. reverse) read sequence (on both strands by using a rolling hash function) in the Fast-SG index and fills a vector of hits of a predefined size. The size of the vector depends on the error rate of the sequencing technology. The default chosen in Fast-SG is of 10 for Illumina and 20 for the long-read technologies. In the second step, the forward (resp. reverse) vector of the *k*-mer hits is sorted by contig and, inside each contig, by coordinate. In the third step, a score is computed for the forward (resp. reverse) read that corresponds to the maximum number of hits falling inside a window of size equal to the length of the read. If the score of both reads in a pair reaches a predefined minimum, in the fourth step the genomic location of the pair is reported. Otherwise, a pair rescue is attempted (fifth step) by fixing the location of the best scored read and looking for a *k*-mer hit in the mate-pair that satisfies the expected distance and orientation (Fig. 1A).

A major parameter of the algorithm is the *k*-mer size as this governs the number of unique *k*-mers to be indexed in a given genome or, in our case, a set of contigs. In Fig. 1B, we show how the number of unique *k*-mers increases as a function of the *k*-mer size in the human genome (GRCh38.p10). However, large *k*-mers need reads with low error rates for a successful match. To define an appropriate *k*-mer size, it is necessary to take into account both the error rate and the length of the query sequence. Almost all short-read aligners use as seeds short *k*-mers (15–32 base pairs) because they have a low probability of containing errors and provide enough specificity [9, 10, 32]. Additionally, the available long-read algorithms such as Canu [4], Lordec [33], and MaSuRCA [34], among others, use short *k*-mers (15–19 base pairs) at some stages to deal with the large error rates (15%) present in the current long-read technologies. In practice, Fast-SG supports a *k*-mer size of up to 256 base pairs. However, for the Illumina reads, values of *k* between 15 and 80 were tested, while for long reads, these ranged from 15 to 22 base pairs, which according to our benchmarks provide enough specificity, even for large genomes (Fig. 1B). There are, for instance, 1.83 billion unique 19-mers (Fig. 1B) in the human genome, which is a good approximation of the nonrepetitive regions for this genome [2].

Another issue of working with *k*-mers is the memory required for storing them for fast look-ups. This was addressed by implementing a novel probabilistic data structure (Fast-SG index) that only requires 87 bits per *k*-mer, while memory increases as a function of the number of unique *k*-mers to store (Fig. 1C). In order to index in memory all the unique *k*-mers of the human genome at a given *k*-mer size (<256 bp), less than 30 Gb of memory is required (Fig. 1C).

Finally, the genomic location of the read pairs is reported using a single representative unique *k*-mer for each read in Sequence Alignment/Map (SAM) format [35], thus allowing for an easy integration with scaffolders that support this standard format. The steps of scoring and pair rescuing follow some of the ideas used in the Ssaha [32] and Bwa-mem [36] aligners.

#### Illumina mate-pair reads alignment

Illumina mate-pair reads are aligned using the algorithm described previously (Fast-SG alignment-free strategy). The forward read (QF) is iterated *k*-mer by *k*-mer where, for each *k*-mer, we ask if it is present in the Fast-SG index until 10 hits are stored in the vector *vectorFUH*. If the score of QF is larger than 3, we attempt to fill the vector *vectorRUH* (QR) of the reverse read. Then, if the score of each read is larger than 5, the positions are reported. Otherwise, we attempt pair rescue by fixing the position of the best-scored read and requiring a minimum score of 4 for the rescued read. These parameters of minimum and pair-rescue scores were set from empirically derived defaults. Such default short-read parameters can be modified by theuser.

#### Extraction of synthetic pairs from long reads

Synthetic pairs of reads (QF and QR) are extracted from the long-read sequences that have a default read length of 200 base pairs in forward-reverse orientation and separated by a distance *D* (insert size). Multiple values of *D* can be specified to comprehensively extract linking information from the long reads. After extracting a synthetic pair, each query sequence (QF and QR) is aligned using the algorithm described previously (Fast-SG alignment-free strategy). A minimum score of 15 and a minimum rescue score of 4 are used as default parameters. Then, as default, a moving window of 100 bp is adopted to extract another pair, until the complete long-read sequence is scanned. The default long-read parameters can be modified by theuser.

#### Estimation of the genomic library parameters

The genomic library parameters for insert size, standard deviation, and orientation are estimated using a subset of the mate-pair sequences in order to use them in the rescue step of Fast-SG. These subsets of mate-pair reads are aligned to the target contigs/genomes, and the read pairs located within contigs are used to estimate the library parameters. For Illumina, we use  100 000 pairs, which are aligned to the target sequences using a minimum score of 8 and without pair rescue. Then, for each aligned pair within contigs, we save the pair orientation and distance. To infer the average insert size and standard deviation, we remove 10% outliers from both tails of the values stored by sorting the observed insert sizes by increasing order. The orientation is computed using a majority rule on the four possible orientations for a pair of reads (FR, RR, FF, RF). For long reads, we use 1 000 long-read sequences and we extract the specified insert sizes to infer the average insert size and standard deviation as for the Illumina reads. The orientation for the synthetic libraries is not estimated because all pairs are created in forward-reverse orientation.

#### Concurrent steps of Fast-SG

The index construction and alignment steps in Fast-SG are concurrent. The Fast-SG index can use multiple threads to construct the Mphf [27] and store the associated *k*-mer information (contig_id, coordinate, strand). Chunks of 5 Mb of contig sequences are used to populate in parallel the Fast-SG index. The Fast-SG alignment step is concurrent by taking chunks of  500 000 and 1 000 for the short and long reads, respectively. The concurrent steps are implemented using the Pthread library. The user specifies the number of central processing units (CPUs) to be used.

### Data description

#### Datasets and software

We collected a comprehensive collection of standard datasets (Table 1) that are frequently used to benchmark the new sequencing technologies, scaffolding tools, or genome assembly pipelines.

**Table 1:** Sequencing datasets and Illumina assemblies used to evaluate the performance of Fast-SG

Long-read datasets						
	Number of reads	Average read length (bp)	Technology	Machine	Illumina assemblies	
					Number of contigs	N50
	164 472	9 009	ONT	R9.2	140	106 241
E.coli K12	1 192 955	4 412	PacBIO	Sequel System		
	22 391 084	298	Illumina	MiSeq		
*S.cerevisiae* W303	594 243	4 795	PacBio	PacBio	890	52 324
	561 176	9 633	PacBio	Sequel System	2 384	320 571
*A. thaliana* (Ler-0)	46 129 480	300	Illumina	MiSeq		
Human (NA12878)	1 415 868	16 324	ONT	R9.4	37 393	202 174
**Short-read datasets**						
	**Number of reads**	**Read length**	**Insert size**	**SRA/ENA**	**Illumina assemblies**	
					**Number of contigs**	**N50**
*S.aureus*	3 494 070	37	3 500	SRR022865	170	47 016
*R.sphaeroides*	2 050 868	101	3 500	SRR034528	577	15 351
*P.falciparum* (short)	52 542 302	76	550	ERR034295	9 318	2 995
*P.falciparum* (long)	1 562 080	75	3 000	ERR163027		
*H.sapiens* chr14	22 669 408	101	2 600	SRR067771	19 936	12 963

Further details are provided in the Data Description subsection and in the Supplementary Material 1.

Long-read datasets were used to investigate the capacity of Fast-SG to extract linking information from long reads and then the performance of short-read scaffolders fed with Fast-SG when compared to a dedicated long-read scaffolder. In the first case, the genome of *Escherichia coli*K12 was adopted as it has been sequenced by multiple long-read technologies and is commonly used to validate the long-read algorithms [4]. In the second case, both *E. coli K12* and *Saccharomices cerevisiae* W303 (Table 1) were used to prove that short-read scaffolders can use synthetic mate-pair libraries extracted from long reads.

To explore the amount of long-read coverage required by the hybrid solutions, we compared the performance of the latter to the results obtained by Canu [4], a state-of-the-art long-read assembler. In the first step, we used the genome of *Arabidopsis thaliana* and then in the second step we used a complete human genome (NA12878, Table 1). NA12878 was selected because it was sequenced on a variety of platforms [17, 20, 22, 37] and assembled by a variety of algorithms [4, 20, 22, 34]. It thus allows comparison of the complete landscape of currently available long-range technologies and assembly pipelines.

To assess the performance of Fast-SG for constructing the scaffolding graph from short reads, we used all the short-read datasets and Illumina assemblies defined in Hunt et al. [3]. These short-read datasets include the genomes of *Staphylococcus aureus*, *Rhodobacter sphaeroides*, and *Plasmodium falciparum* and the human chromosome 14 (Table 1) and are commonly used as the gold standard for validation of the scaffolding tools [11-14].

We coupled Fast-SG with two well-established scaffolders, Opera-LG [11] and Besst2 [12], and two more recently published scaffolders, ScaffMatch [13] and Boss [14], to produce scaffold sequences from short- or long-read data. All the chosen scaffolders have different algorithms to select optimal paths from the scaffolding graph and use the Sam/Bam format as input. Besst2 was excluded from the hybrid scaffolding experiments due to an exception produced while Besst2 computes the average contig coverage from synthetic mate-pair libraries. All the software and reference genomes used are described in Supplementary Material 1.

#### Short- and long-read benchmarks

All scaffold sequences generated from alignments produced by Fast-SG, by the short-read aligners, and by Links were evaluated following the standard defined by Hunt et al. [3]. For each dataset, the true contig layout is known and the scaffold sequences were compared against it in order to determine the following scaffolding errors (represented as a bit-wise flag):

**0** = Correct pair of contigs.
**1** = Contigs originated from same reference sequence, but their orientation in the scaffolds is incorrect.
**2** = Contigs originated from different reference sequences.
**4** = Contigs originated from the same reference sequence but are the wrong distance apart.
**5** = **4+1**, Contigs originated from same reference sequence, but their orientation and distance in the scaffold are incorrect.
**8** = Contigs originated from the same reference sequence but are not in the correct order.
**12** = **8+4** Contigs originated from the same reference sequence but are not in the correct order and distance.

From the previous values, we computed the F-score metric, which was first introduced by Mandric and Zelikovsky [13] and adopted in Luo et al*. *[14], also with the purpose of improving and summarizing in a single metric the performance of a scaffolding tool. In brief, if we denote *P* as the number of potential joins that can be made, *TP* as the number of correct joins performed by a scaffolder (true positives), and *FP* as the number of wrong joins (false positives), we can calculate the following quality metrics:

\begin{equation*}Recall=\frac{TP}{P}
\end{equation*}

\begin{equation*}
Precision=\frac{TP}{(TP+FP)}
\end{equation*}

\begin{equation*}
F-Score=2*\frac{(Recall*Precision)}{(Recall+Precision)}
\end{equation*}

The structural quality of the hybrid and *de novo* assemblies was determined via direct comparison with the nearest reference genomes available using Nucmer [38] and reported using the Gage statistics [26], which from 1-to-1 alignments evaluates both the identity and the structural breakpoints (inversions, relocations, and translocations). All commands executed in each benchmark are specified in Supplementary Materials 2–5.

### Results

#### Extracting synthetic mate-pair libraries from long reads

Despite the high per-base error rate of the long-read technologies, the long-range information encoded in a long read has proven to be highly accurate. On the other hand, current experimental protocols to produce long-range mate-pair libraries using short-read technologies are time consuming and expensive [23, 24]. Moreover, library contamination occurs when the circularization step fails during construction, resulting in mate-pairs with short insert size and in the wrong orientation [12]. Extracting synthetic mate-pair libraries directly from long reads could improve the performance of the current short-read scaffolders and replace the need for sequencing multiple mate-pair libraries for scaffolding.

To demonstrate the utility of Fast-SG to create synthetic mate-pair libraries from long reads, we collected the latest chemistry data sequenced with the Oxford Nanopore (ONT; 1D reads sequenced on R9.2 flow cells) and Pacific Biosciences (PacBio; Sequel System) technologies for the genome of *E. coli* K12 (Table 1). The long reads were error-corrected using Illumina reads (Supplementary Material 2) with Lordec [33], a hybrid error-correction method.

**Figure 2:**
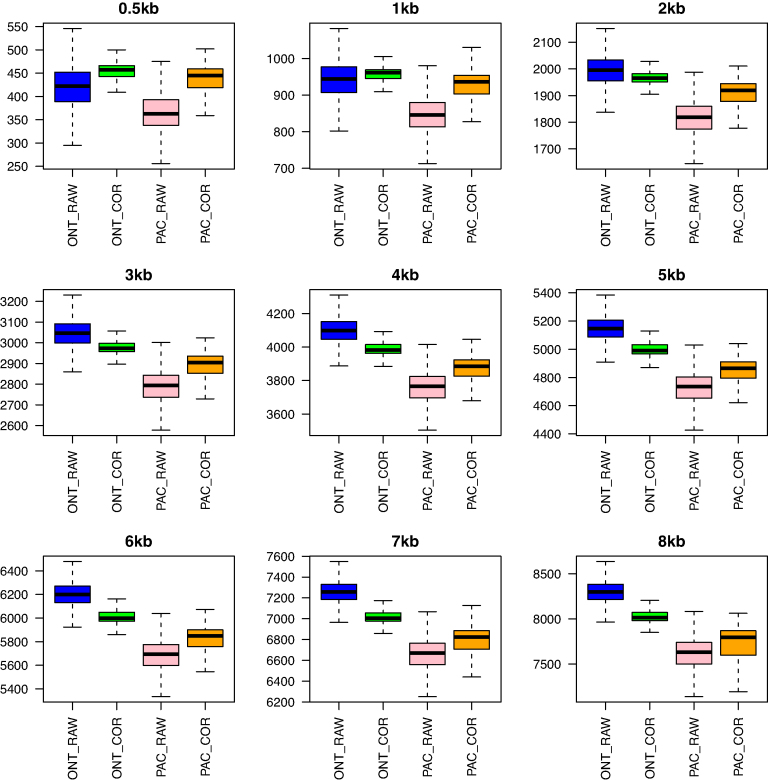
Box plots of the insert size distribution observed for each synthetic library in the genome of *Escherichia coli* K12. The box plots were drawn extracting from the Fast-SG alignments a minimum of 5 000 insert sizes from the mate-pair reads mapped within contigs for each combination of synthetic library and long-read technology. The percentage of outliers detected in the raw ONT reads ranged from a minimum of 0.37% (0.5 kb) to a maximum of 4.24% (8 kb), while for raw PacBio it ranged from a minimum of 0.25% (0.5 kb) to a maximum of 9.85% (8 kb). The number of outliers increased with the error correction for both long-read technologies, reaching an average of 9.32% (std 1.73%) and 8.32% (std 3.74%) for the ONT and PacBio reads, respectively. The box plots were drawn excluding outliers.

Fast-SG was used to generate synthetic mate-pair libraries in the range of 0.5–8 kb from the corrected and uncorrected long reads using a *k*-mer size of 15, at which 98% of the *k*-mers are unique in the reference *E. coli* K12 genome. Synthetic mate-pair reads were aligned to an Illumina assembly of *E. coli K12* (Table 1). Near-perfect synthetic mate-pair libraries were obtained with a low percentage of outliers (<9.85%) for all insert sizes (Fig. 2). Moreover, the hybrid error correction reduced the standard deviation and allowed the average insert size to get close to the specified size of each synthetic library. However, the hybrid error correction increased the number of outliers in both technologies (Fig. 2). The observed average insert size (Fig. 2) in the synthetic libraries from ONT are slightly higher than the observed ones in PacBio, thus reflecting the nature of the error of each long-read technology, which are deletions for ONT [4] and substitutions for PacBio [4].

We computed the recall achieved by Fast-SG at the levels of the *k*-mers and of the synthetic mate-pair reads (the length of the forward and reverse reads equals 200 base pairs) for each long-read technology from either raw or corrected reads (Supplementary Table S8). At the *k*-mer level, Fast-SG has a recall of 8.3% and 5.05% for the uncorrected reads of ONT and PacBio, respectively. The hybrid error correction increased the *k*-mer recall by 10% for both long-read technologies. At the synthetic mate-pair read level, we observed a recall of 49.42% and 31.65% for the raw ONT and raw PacBio reads, respectively. The hybrid error correction increases the synthetic mate-pair read recall for ONT to 75.12% and for PacBio to 65.02%. We observed that Fast-SG is more effective aligning synthetic mate-pair reads from raw ONT than from raw PacBio reads. We expect that this is due to the nature of the ONT errors (major deletions) as Fast-SG is designed to deal with short indels. Despite the low *k*-mer recall, Fast-SG achieved a decent synthetic mate-pair read recall on this dataset from both long-read technologies and extracted near-perfect synthetic mate-pair libraries. The synthetic mate-pair libraries can be used as input to a short-read scaffolder to generate scaffold sequences through a combination of short- and long-read technologies.

#### Comparison of Fast-SG coupled with short-read scaffolders against Links

We compared the results obtained by Fast-SG coupled with Opera-LG [11], ScaffMatch [13], and Boss [14] against Links [39], which is a scaffolder specifically designed to extract paired *k*-mers from long reads and use them to join contigs.

Fast-SG and Links were applied with default parameters (*k*-mer of size 15) to create the synthetic mate-pair libraries in the range of 0.5–8 kb using as input the uncorrected long reads and Illumina assemblies available for both species (Table 1). Since Links performs better with high long-read coverage [39], we subsampled 50X and 30X of coverage from *E. coli* K12 and *S. cerevisiae* W303, respectively.

Fast-SG is two times faster than Links and requires two orders of magnitude less memory to extract linking information from the long reads (Supplementary Table S9). The percentages of linked pairs extracted by both methods is comparable (with Fast-SG being slightly superior). As expected, the percentage of linked pairs increases as a function of the insert size length for both long-read technologies (Supplementary Table S10).

**Figure 3:**
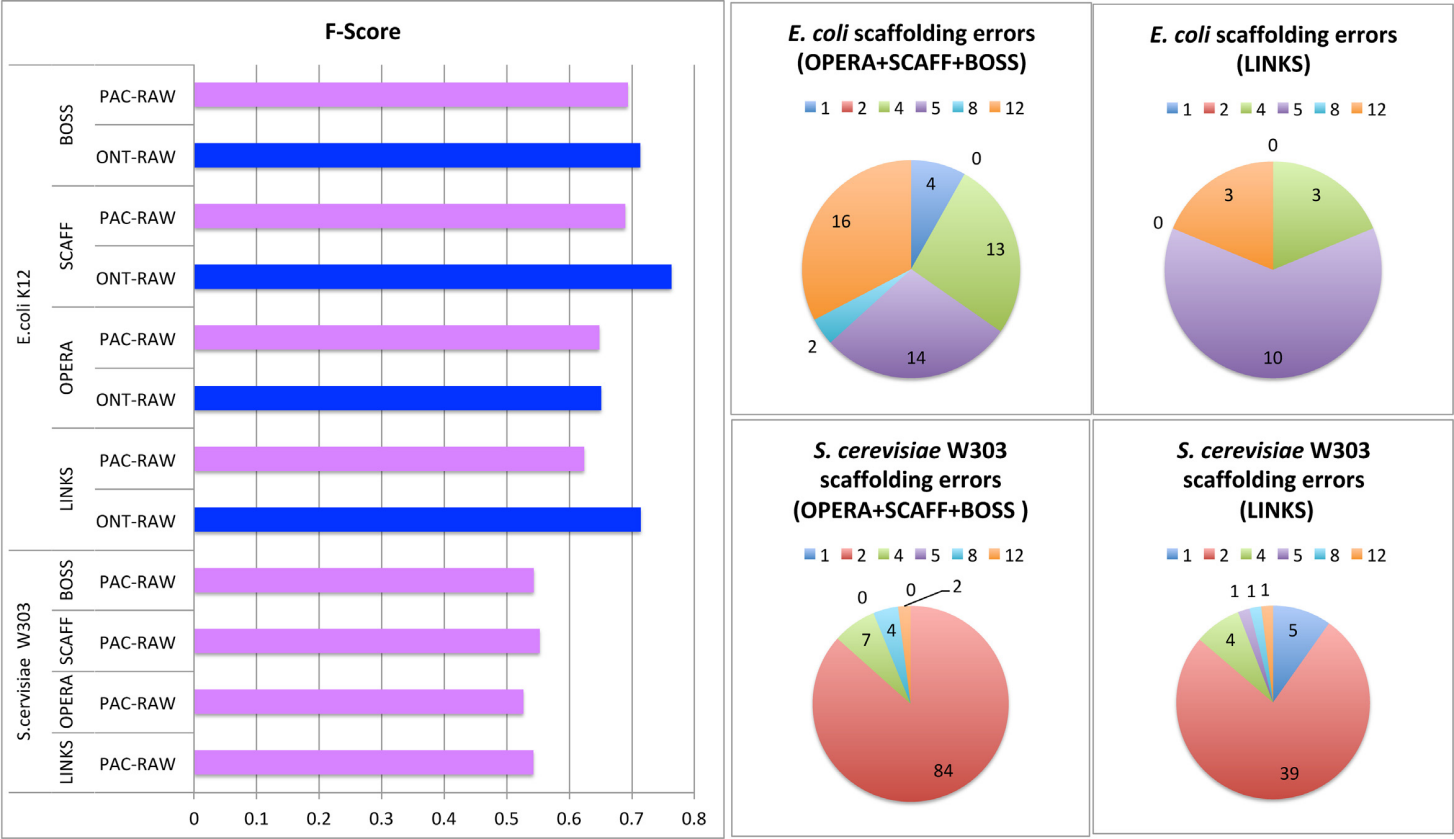
Synthetic libraries scaffolding benchmark. The F-score (Methods section) was computed with the scripts provided by Hunt et al. [3] on the scaffold sequences produced by each scaffolding tool. The pie charts show the number of scaffolding errors for Links and for the short-read scaffolders fed with the Fast-SG alignments for both *E. coli* K12 and *S. cerevisiae* W303. The definition of the scaffolding errors (colors in pie chart) are provided in the Short- and long-reads benchmarks subsection

A more informative comparison involved assessing the quality of the scaffolds [3] produced by Links on one hand, and on the other, by the short-read scaffolders coupled with Fast-SG. To evaluate the scaffolding results, the number of correct and erroneous joins were computed in each test case using the scripts provided in Hunt et al. [3]. Moreover, the F-score metric (Short- and long-reads benchmarks subsection) was used to summarize in a single statistic the performance of each scaffolder. Based on the F-score values, the short-read scaffolders using Fast-SG reached better or comparable results than Links (Fig. 3). Moreover, Links produced more scaffolding errors in two out of the three datasets tested (Supplementary Table S11). With respect to the *E. coli* dataset, the scaffolding errors made by the short-read scaffolders using Fast-SG (Fig. 3) were related to the gap size estimation (type error 4), orientation (type errors 1 and 5), and relocation (type errors 8 and 12). The major source of errors in the scaffolds produced by Links was of type 5. This measures the correct orientation and distance between pairs of contigs (Fig. 3). On the *S. cerevisiae* W303 dataset, the major source of scaffolding errors was translocation (type error 2) for both methods. However, Links has almost double the number of scaffolding errors compared to Fast-SG coupled with Opera-LG or Boss on this dataset (Fig. 3, Supplementary Table S11).

Concerning the short-read scaffolders, Boss and ScaffMatch reached higher F-score values than Opera-LG (Fig. 3). However, they tended to produce more scaffolding errors (Supplementary Table S11). It is important to notice also that the scaffolding errors observed here can be further reduced because fragmented Illumina assemblies (Table 1) were used in order to maximize the possibility of the scaffolders to make joins.

Overall, the performance of the short-read scaffolders coupled with Fast-SG was superior or comparable to Links, a scaffolder specifically designed for long reads. Fast-SG thus allows the conversion of tools designed for short-read scaffolding into a long-read scaffolder in a fast and modular way.

#### Using Fast-SG to perform the hybrid assembly of Arabidopsis thaliana (Ler-0)

An important goal of hybrid assembly methods is to reduce the long-read coverage required to produce long-range genome assemblies. Here, we examine the long-read coverage required by our hybrid assembly method to produce long-range hybrid assemblies comparable to Canu [4], which is a state-of-the-art *de novo* long-read assembler.

Briefly, the hybrid assembly using Fast-SG proceeded as follows. In the first step, a single Illumina library (Table 1) covering 100X the *A. thaliana* (Ler-0) genome was assembled using DiscovarDeNovo [37], which is one of the best tools for assembling a single Illumina fragment (paired-end) library. The resulting assembly contained 2 384 scaffolds with a N50 of 320 kb and a total size of 119 Mb (Table 2). The DiscovarDeNovo assembly took 6.6 hours on 20 CPUs. In a second step, 50X PacBio reads (P5-C3) were error-corrected (Table 1), with the same Illumina reads used for the *de novo* assembly, using Lordec. Lordec took 14.2 hours on 20 CPUs. In a third step, the error-corrected long reads were randomly subsampled with a coverage between 5X and 50X, and Fast-SG (using 21-mers) was used to create 12 synthetic mate-pair libraries in the range of 1–20 kb for each subsample. The total number of mate-pair reads aligned at each coverage value ranged from 11.85 to 104.99 million for 5X to 50X, respectively (Supplementary Table S12). On average, 7.2% of the synthetic mate-pair reads aligned by Fast-SG were linking (i.e., connecting two different contigs) in each subsample. Moreover, a near perfect insert size distribution and a low percentage of outliers were observed for each synthetic library (Supplementary Fig. S1). Fast-SG took 2.15 hours on 20 CPUs to process the whole dataset. Finally, Opera-LG, Boss, and ScaffMatch were fed with the Fast-SG alignments to produce the scaffold sequences (Table 2). All short-read scaffolders generated scaffold sequences in at most half an hour (Opera-LG 22 min, Boss 24 min,and ScaffMatch 30 min) using a single CPU.

**Table 2:** Hybrid and long-read assemblies of *Arabidopsis thaliana* (Ler-0)

Number of scaffolds	MAX	N50	Size (Mb)	Fold	Long Read Coverage	Scaffolder /Assembler	Breakpoints			1-to-1 identity	% Ref covered
							Number	Bases (Mb)	% Error		
2 384	1 551 485	320 571	119.45	1.00	-	Discovar	91	0.48	0.49	99.07	82.044
1 577	5 305 497	1 076 408	120.05	3.36	5X	Opera-LG	174	0.978	1.00	99.07	82.054
1 368	9 953 317	2 475 756	120.26	7.72	10X	Opera-LG	202	1.197	1.22	99.07	82.047
1 249	16 906 870	4 165 132	120.32	12.99	15X	Opera-LG	206	1.237	1.26	99.07	82.052
1 179	18 032 662	4 941 257	120.41	15.41	20X	Opera-LG	218	1.588	1.62	99.07	82.060
1 103	14 710 653	4 756 724	120.43	14.84	30X	Opera-LG	227	1.728	1.76	99.07	82.055
1 049	10 003 725	4 667 601	120.41	14.56	50X	Opera-LG	230	1.732	1.76	99.07	82.060
1 345	8 867 374	1 632 787	120.40	5.09	5X	ScaffM	195	1.620	1.65	99.07	82.058
1 143	8 867 059	5 142 417	120.65	16.04	10X	ScaffM	203	1.319	1.34	99.07	82.045
1 072	11 814 750	6 165 459	120.73	19.23	15X	ScaffM	205	1.330	1.36	99.07	82.045
1 020	11 873 221	6 221 109	120.80	19.41	20X	ScaffM	207	1.477	1.50	99.07	82.039
958	13 946 812	7 073 179	120.90	22.06	30X	ScaffM	209	1.651	1.68	99.07	82.042
923	13 957 620	6 292 557	120.85	19.63	50X	ScaffM	210	1.712	1.74	99.07	82.041
1 593	5 296 335	1 037 785	119.96	3.24	5X	Boss	179	1.171	1.19	99.07	82.061
1 371	13 608 688	2 554 739	120.17	7.97	10X	Boss	200	1.335	1.36	99.07	82.054
1 239	13 643 115	2 829 628	120.22	8.83	15X	Boss	207	1.189	1.21	99.07	82.061
1 173	7 977 908	3 005 451	120.23	9.38	20X	Boss	212	1.564	1.59	99.07	82.060
1 093	9 004 636	2 974 378	120.28	9.28	30X	Boss	219	1.575	1.60	99.07	82.057
1 031	11 011 921	3 179 270	120.29	9.92	50X	Boss	229	2.162	2.20	99.07	82.050
1 439	447 211	80 063	89.84	-	10X	Canu	107	0.675	1.10	98.19	51.188
259	4 542 617	1 170 676	118.25	-	20X	Canu-p	201	0.969	0.99	99.06	81.907
258	4 543 625	1 170 942	118.31	-	20X	Canu-q	183	0.831	0.85	99.02	81.808
259	4 535 400	1 168 180	118.05	-	20X	Canu	185	1.030	1.09	98.82	78.874
119	15 152 700	6 219 401	120.67	-	50X	Canu	219	1.766	1.79	99.02	82.565
88	15 945 651	8 307 845	121.45	-	150X	Canu	215	1.935	1.95	99.06	82.938

Continuity was measured using maximum and N50 contig/scaffold size, where N50 is the contig/scaffold length such that half of the assembly size is obtained by adding contigs/scaffolds sorted in descending order by length. The quality of the assembly was evaluated via a direct comparison against the *A. thaliana* TAIR10 reference genome using Nucmer [38] and reported using the Gage [26] statistics, which from 1-to-1 alignment evaluates both identity and structural breakpoints (inversions, relocations, and translocations). An optimal assembly has high continuity, low breakpoint errors, high identity, and high coverage of the reference genome. Canu-p and Canu-q are Canu assemblies polished with Pilon [48] and Quiver, respectively. Pilon and Quiver are tools used after a long-read assembly to improve the quality of the consensus sequence. All datasets and commands used for the hybrid assembly of *A. thaliana* (Ler-0) are detailed in Table 1 and Supplementary Materials 2 and 3.

The hybrid and the Canu assemblies available were structurally validated by a whole genome alignment against the reference *A. thaliana* TAIR10 genome (Table 2, Supplementary Material 2).

As can be seen in Table 2, all hybrid assembly pipelines were able to produce long-range scaffolds (N50 >1 Mb) with a high coverage of the reference genome, low number of errors (<2.2%), low amount of sequence gaps (1.46 Mb as maximum), and with an identity higher than any Canu assembly. All hybrid assemblies at 5X coverage reached a N50 scaffold size comparable to the contig N50 obtained by a polished Canu assembly requiring 20X of coverage and 100X of Illumina reads (Table 2). Additionally, all hybrid assembly pipelines seemed to plateau after 30X of long-read coverage as was previously observed for this dataset [4]. However, ScaffMatch, the most aggressive scaffolder tested, at 10X–30X of coverage produced accurate scaffolds having an N50 comparable to the Canu assemblies requiring 50X or 150X of coverage (Table 2).

All assemblies of *A. thaliana* (Ler-0) were comparable in terms of the number and amount of sequences involved in structural errors (Table 2). Moreover, the major source of structural errors observed in both assembly strategies were mainly relocations, which explains more than 50% of the amount of sequences involved in miss-assemblies (Supplementary Fig. S3).

Overall, we demonstrated that the hybrid assemblies were comparable in terms of continuity, completeness, and accuracy to the assemblies obtained by Canu, which is considered a state-of-the-art *de novo* long-read assembly pipeline. Furthermore, the proposed hybrid assembly strategy allowed faster and cheaper reconstructions of the *A. thaliana* (Ler-0) genome and was remarkably efficient at shallow long-read coverage (5X–10X).

#### Using Fast-SG to perform the hybrid assembly of a diploid human genome (NA12878)

An ultimate benchmark for any assembly method or sequencing technology is to assemble a complete human genome [4, 20, 22, 34, 40]. We performed a hybrid assembly of the Utah/Ceph NA12878 human diploid genome using a low coverage (5X) of ultra-long Nanopore reads (Table 1, [17]), a DiscovarDeNovo assembly built from 50X of 250 bp Illumina reads (Table 1, [37]), Fast-SG, and ScaffMatch [13].

Fast-SG (using 22-mers) was run to create 20 synthetic mate-pair libraries in the range of 2–180 kb using as input a total of 1.4 million uncorrected Nanopore reads (N50 64.75 kb, Table 1), which have a total size of 23.11 Gb and cover about 7X that of the human genome. A total of 455.9 million synthetic mate-pair reads (11.15% linking contigs, Supplementary Table S13) were aligned to the DiscovarDeNovo assembly, with a near-perfect distribution of insert sizes and a low percentage of outliers observed (Supplementary Fig. S2). Fast-SG required 8 hours using 20 CPUs to complete the task and used a maximum of 25 Gb of memory. ScaffMatch was then fed with the alignments of Fast-SG and took 5.18 hours using a single CPU with a peak memory of 30.87 Gb to generate the scaffold sequences. The resulting hybrid assembly is referred to here as the DFS (DiscovarDeNovo+Fast-SG+ScaffMatch) assembly.

We evaluated the accuracy of the DFS assembly together with the public assemblies of NA12878 that were built using Canu [17], MaSuRCA [34], 10X genomics [22], and DOVETAIL genomics [20] by means of whole genome alignments against the complete human reference genome (Table 3).

**Table 3:** Hybrid and long-read assemblies of NA12878

		Discovar	Dfs	10X	Dovetail	Canu-p	MaSuRCA
**Assembly statistics**	Number	37 393	7 323	9 926	9 463	2 337	4 885
	Min.	2 000	2 000	2 000	2 000	2 981	4 103
	Max.	1 380 479	30 548 185	69 726 354	95 295 052	50 410 306	9 066 374
	N50	202 174	6 445 123	16 305 019	24 472 662	7 667 013	1 695 766
	Size	2 794 627 041	2 884 349 664	2 835 096 130	2 800 321 128	2 866 880 913	2 849 443 591
**Long-read coverage**		-	7X	-	-	35X	7X
**1-to-1 alignments**							
	Length	2 793 980 166	2 797 898 328	2 778 947 064	2 799 630 879	2 811 439 829	2 845 550 340
	Identity	99.8	99.8	99.79	99.8	99.28	99.67
	% Ref covered	90.16	90.29	89.68	90.35	90.73	91.83
**Breakpoints**							
Relocations	Number	120	1 151	688	997	501	374
	Bases (Mb)	0.361	5.604	4.810	0.582	2.281	2.071
Translocations	Number	373	1 856	883	976	1 082	941
	Bases (Mb)	4.840	11.279	7.838	6.576	13.781	13.933
Inversions	Number	53	768	871	2,813	299	240
	Bases (Mb)	0.151	3.886	7.273	0.736	2.903	3.008
Total	Number	546	3 775	2 442	4 786	1 882	1 555
	Bases (Mb)	5.353	20.769	19.921	7.894	18.964	19.012
	%1-to-1	0.192	0.742	0.717	0.282	0.675	0.668

Assembly statistics: *Number* - number of contigs/scaffolds assembled; *Max/Min* - the maximum/minimum contig/scaffold size in base pairs; *N50* - contig/scaffold length such that half of the assembly size is obtained by adding contigs/scaffolds sorted in descending order by length; *size* - total size of the assembly in base pairs; 1-to-1 alignments: *length* - total length of nonrepetitive alignments between the assembly and GRCh38.p10 detected by Nucmer; *identity* - average identity between the assembly and GRCh38.p10 computed from the 1-to-1 alignments; *%Ref covered* - percentage of the GRCh38.p10 that is covered by 1-to-1 alignments where the length of the reference was set to 3.1 Gb; *Breakpoints -* structural errors were obtained from 1-to-1 alignments and reported using the Gage metrics (relocations, translocations, and inversions); *number* - counts the number of breakpoints by sort; *bases (Mb)* - adds the number of bases involved in breakpoints extracted from the Dnadiff report (qdiff file) in mega bases; *%1-to-1* - percentage of structural errors with respect to the total 1-to-1 alignment length. Public NA12878 assemblies were downloaded and used for validation and comparisons against the DFS hybrid assembly pipeline.

In terms of continuity (N50, Table 3), the DFS assembly is more than 4X larger than a MaSuRCA hybrid assembly built with the same long-read dataset and 100X of Illumina reads [49]. Moreover, it is comparable to a polished Canu assembly built with 35X of long-read coverage [17]. DOVETAIL genomics and 10X genomics reached larger N50 scaffolds (Table 3), which are 2.5X and 3.7X larger than the DFS assembly, respectively. All assemblies are comparable in terms of size, 1-to-1 alignment length, and coverage of the reference genome (Table 3).

In terms of identity (Table 3), DOVETAIL genomics and DFS are the leading pipelines. DOVETAIL genomics and DFS both use the DiscovarDeNovo assembly as input for scaffolding. Both software programs maintain the high identity of the DiscovarDeNovo assembly because contig bases are not changed in the scaffolding process.

Regarding the structural errors, all assembly pipelines are highly accurate with less than 1% of the total 1-to-1 alignment length involved in such errors (Table 3, Supplementary Fig. S4). Moreover, translocation is the structural error that accumulates the greatest amount of miss-assembled bases on all assembly pipelines (Table 3). A more detailed inspection of the 1-to-1 alignments revealed that DFS, 10X genomics, and DOVETAIL genomics tend to skip the short contigs (Supplementary Table S14), which is a known problem of scaffolding tools [3]. However, more complex miss-assemblies involving several structural errors were observed in the chimeric contigs assembled by Canu and MaSuRCA (Supplementary Table S15).

In terms of speed, the whole DFS pipeline (933 CPU hours) was 22X times faster than MaSuRCA (21 000 CPU hours; personal communication), 162X times faster than Canu (151 000 CPU hours [17]), and comparable to 10X genomics and DOVETAIL genomics.

Finally, we call attention to the fact that the hybrid assembly solution that we propose (using 14 ONT flow cells and 50X of 250 bp paired-end reads sequenced on Hiseq2500) is approximately 3 times cheaper than the Canu solution (using 53 flow cells and 50X of Illumina).

In summary, we demonstrated in this experiment that the DFS hybrid assembly pipeline produced an accurate and long-range reconstruction of a diploid human genome that was faster and cheaper than the current state-of-the-art long-read assembly pipelines.

#### Compatibility of Fast-SG with Illumina mate-pair libraries

In this section, we explore the usefulness of Fast-SG as an alternative to commonly used short-read alignment software for scaffolding graph construction from short-read data. Indeed, Hunt et al. [3] demonstrated that the quality of the scaffolding results is highly dependent on the short-read aligner being used and that precision is more important than maximizing the number of reads aligned to the contigs.

We assessed the performance of Fast-SG for aligning short reads on simulated Illumina data from the complete human reference genome (GRCh38.p10, Supplementary Material 4) together with Bowtie [8], Bowtie2 [10], Bwa-Mem [36], and Bwa [9], which are commonly used short-read aligners for constructing a scaffolding graph [3].

Our results show that the Fast-SG precision is high for any *k*-mer size (99.21% as minimum), is superior to Bowtie2-local (98.17%), and is comparable to Bowtie2-global (99.74%). However, Bwa-Mem (99.97%) is the leading tool (Supplementary Table S16). In terms of speed, Fast-SG performs the best. Indeed, it is between 7X and 14X times faster (depending on the *k*-mer size) than the next fastest program, which is Bowtie2-global (Supplementary Table S16). The recall of Fast-SG depends on the *k*-mer size used (Supplementary Table S16, Fig. 1B). The recall of Fast-SG (71.67%) is comparable to Bowtie (71.52%) for optimal *k*-mer values (*k* = 25-30). Larger *k*-mer values (*k*>50) decrease the recall of Fast-SG due to sequencing errors and read length. To map short reads of 101 base pairs in length, we therefore recommend use of*k*-mer values in the range of 25 to 30 base pairs.

A more informative evaluation consists of assessing the performance of Fast-SG on real Illumina data. Such evaluation was done on four real test cases (Table 1) and using four short-read scaffolders. The short reads were aligned using Fast-SG and the aforementioned short-read aligners. The scaffolders were fed with such alignments and run with identical commands overall (Supplementary Material 5).

In relation to the number of paired reads mapped (Supplementary Fig. S5), Fast-SG aligned on average more pairs than Bowtie or Bwa and was comparable to Bowtie2-global. However, it aligns fewer pairs than Bowtie2-local or Bwa-Mem. From the number of paired reads aligned across the four test cases, we noticed that the behavior of Fast-SG depends on the *k*-mer size chosen. With larger sizes, Fast-SG resembles global methods, while with shorter sizes, it is closer to local methods (Supplementary Fig. S5).

The average contig read-coverage statistic that is used to tag the repeated contigs before scaffolding [2] was extracted from the results of Opera-LG. Such statistics were used to compute a pairwise Pearson correlation to determine the linear relationship between the short-read aligners and Fast-SG (Supplementary Fig. S6). We observed that the average contig read-coverage computed from the Fast-SG alignments correlated more on average with Bowtie ($\overline{x}$=0.933), Bwa ($\overline{x}$=0.905), and Bowtie2-global ($\overline{x}$=0.814) than with Bwa-Mem ($\overline{x}$=0.772) or Bowtie2-local ($\overline{x}$=0.725) on the datasets of *S. aureus*, *R. sphaeroides*, and *P. falciparum* (Supplementary Fig. S6).

**Figure 4:**
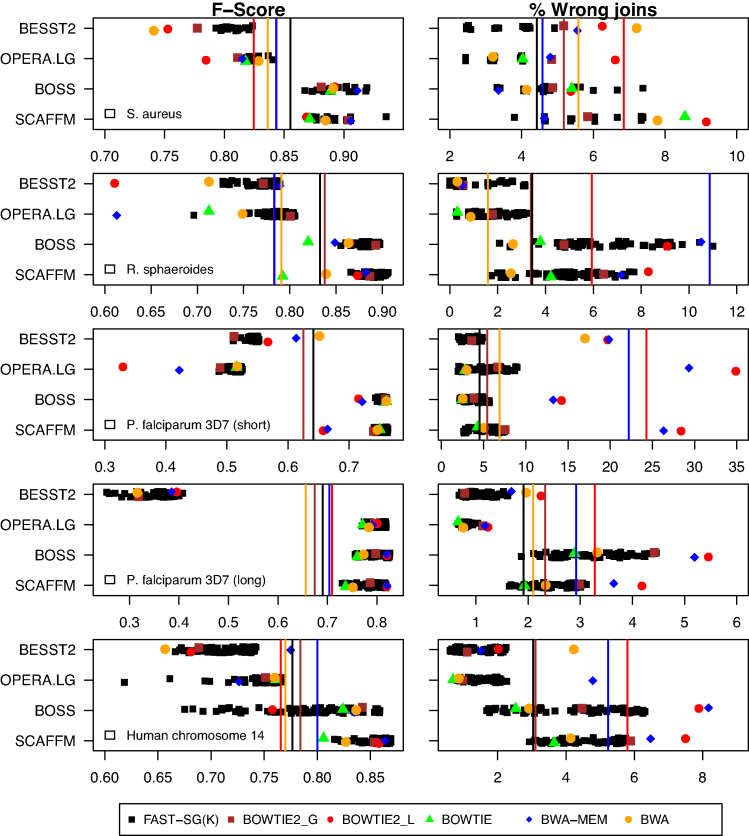
Illumina scaffolding benchmark. Four real datasets (Table 1), five Illumina libraries, and four scaffolding tools were used to assess the performance of Fast-SG and the short-read aligners for building the scaffolding graph by means of an F-score metric and percentage of wrong joins (Algorithms section, and Supplementary Material 4). Fast-SG was run with various *k*-mer sizes in the range of *k* = 12–28, *k* = 12–70, *k*= 15–66, and *k* = 15–80 for *Staphylococcus aureus*, *Rhodobacter sphaeroides*, *Plasmodium falciparum*, and the human chromosome 14, respectively. Short-read aligners were run with the wrapper or instructions provided by the scaffolding tools when possible or using the default parameters. Single data points provide the F-score and error rate for each combination of scaffolding tool and aligner in each dataset. The vertical lines show for each dataset the average F-score or error rate values obtained by each of the short-read aligners or Fast-SG together with the four scaffolding tools. Vertical lines for Bowtie were not plotted since it cannot be used with Besst2. For the *P. falciparum* (short) dataset, the average F-scores (vertical lines) were omitted for Bwa, Bwa-mem, and Bowtie2-Local due to poor performance (high error rate). The commands used for the aligners and scaffolding tools are detailed in Supplementary Material 5.

The results of the four test cases in terms of F-score and error rate are illustrated in Fig. 4 and detailed in Supplementary Tables S17 to S20. For almost all the test cases and scaffolding tools, Fast-SG reached the largest F-score (Fig. 4) for some *k*-mer values. Moreover, Fast-SG had a superior average performance in terms of F-score in relation to the four scaffolders tested in two of the five datasets (Fig. 4, vertical lines) and allowed the scaffolding tools to obtain more accurate scaffolding results in four of the five datasets (Fig. 4, vertical lines).

The low GC content genome of *P. falciparum* proved to be particularly challenging to the scaffolders using local alignment methods (namely, Bwa-Mem or Bowtie2-local). These indeed tended to produce several wrong joins (Fig. 4), indicating that the local alignment methods are not an appropriate choice for scaffolding this genome. A possible explanation for the poor performance observed in this particular case is that the local alignment methods mapped 10% more reads than the global ones and more than Fast-SG (Supplementary Fig. S5). However, there is a low correlation in the average contig read-coverage between the local alignment methods and Fast-SG (Supplementary Fig. S6), suggesting many wrong mappings in the extra 10% aligned reads.

In conclusion, over the four test cases and four scaffolders benchmarked, Fast-SG consistently reached better scaffolding results than the short-read aligners evaluated and may be considered as an effective tool for constructing a scaffolding graph from short reads.

#### Procedure for effective hybrid assembly with Fast-SG

The *de novo* assembly of a large genome is a difficult task. Genome complexity (size, repetitiveness, heterozygosity, polyploidy), as well as the algorithm and the sequencing platform adopted, are all factors that may affect the quality of the resulting assembly. Here, we provide a procedure for an effective hybrid assembly using Fast-SG that is based on our experience and benchmark results.

**Figure 5:**
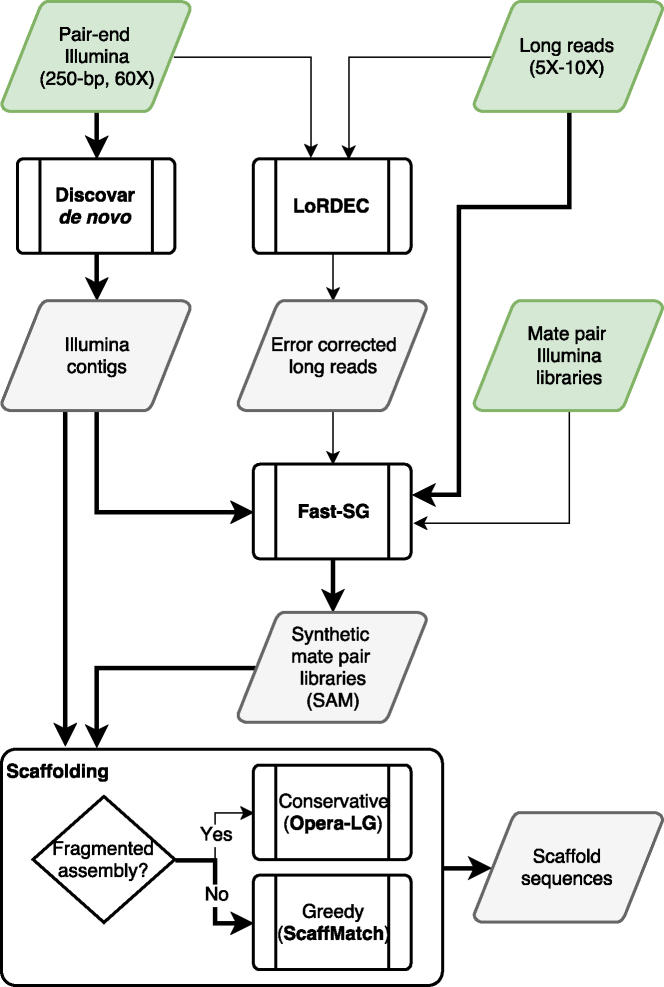
Fast-SG hybrid assembly workflow. Thick black lines represent the common path for hybrid assembly with Fast-SG. Thin black lines show alternative paths when long reads were error-corrected or Illumina mate-pair libraries were sequenced.

The first step is to produce the best possible Illumina contig assembly (Fig. 5, N50 >100 kb). To achieve this, we recommend the use of a single Illumina fragment library (paired-end) prepared using a Polymerase Chain Reaction (PCR)-free protocol (550 bp insert size). The Illumina library should be sequenced using either the MiSeq or the HiSeq2500 platform to generate paired reads of 250 bases at about 60X of genome coverage [37]. The Illumina library must be assembled with a de Bruijn graph assembler supporting a large *k*-mer size (*k*  = 200) or a multi *k*-mer approach [41]. We tested DiscovarDenovo (*k* = 200) and obtained good-quality contigs (N50 >100 kb), but either Abyss [42] or Spades [41] can be used to create the contigs.

The second step is to sequence at shallow coverage (5X–10X) the longest possible reads by using the ONT or PacBio technologies (Fig. 5). At the moment, we recommend the use of 1D ONT reads because the latest ONT machines produce longer reads than PacBio machines and Fast-SG is more adapted to the error profile of ONT than of PacBio. In cases where ONT reads are not available, we recommend hybrid error-correcting of the PacBio reads using Lordec (*k* = 19 − 21) before applying Fast-SG (Fig. 5).

The third step is to use Fast-SG to comprehensively extract linking information from the long reads by creating multiple-insert-size synthetic mate-pair libraries that lead to an improved scaffolding [43, 44]. In practice, we were able to create synthetic mate-pair libraries in the size ranges of 2–20 kb and 2–180 kb from PacBio and ONT (ultralong reads), respectively. The *k*-mer size parameter of Fast-SG depends on the quality of the long reads. With raw long reads, we recommend using short *k*-mer sizes (*k*= 17−22) to overcome the high error rate. Larger *k*-mer sizes (*k*= 17−40) can be used with error-corrected long reads or with Illumina mate-pair libraries (Fig. 5). After running Fast-SG, we recommend verifying the quality of each synthetic mate-pair library generated. To check the synthetic libraries, it is possible to plot the distribution of the observed insert size statistics that are computed from the read pairs aligned within contigs. Figure 2 and the Supplementary Figs. S1 and S2 provide examples of such distribution. Additionally, statistics of the percentage of outliers and standard deviation can be computed from the observed insert sizes. For instance, a high percentage of outliers (>30%) and a larger-than-expected standard deviation (>30% of average) are both indicative of a low-quality synthetic library. The latter must be discarded from the scaffolding step. Fast-SG computes and reports (log file) the observed average insert size for each synthetic library, which allows for an easy identification of low-quality synthetic libraries.

The fourth step is to select a short-read scaffolder. We showed that there are two classes of short-read scaffolding tools, one more conservative (this class includes Opera-LG and Besst2) and another more greedy (including Boss and ScaffMatch). The greedier scaffolders reach higher F-score values than the conservative ones. However, the greedy ones tend to produce more scaffolding errors (Fig. 4). According to our evaluations, we recommend a more greedy scaffolder (ScaffMatch) when the Illumina contig assembly is not highly fragmented (N50 >100 kb). Otherwise, a more conservative scaffolder (Opera-LG) should be used to avoid scaffolding errors.

Finally, a full hybrid assembly example is described step-by-step in the following wiki-page of Fast-SG [50].

#### Discussion

The proposed hybrid assembly method could be improved by using the sequence between the synthetic mate pairs (inner sequence), either for assigning a new weight to the edges before scaffolding or for placing the skipped contigs after scaffolding. An edge of the scaffolding graph can be reweighted by computing the edit distance among the inner sequences and then eliminating the pairs that have a large edit distance. Edlib [45] is an efficient library that could be used to perform this task. The skipped contigs can be unambiguously placed by computing a consensus sequence of the scaffolding gaps from the inner sequences and then aligning the skipped contigs to the consensus gap sequence, taking into account the lengths of the gap and of the skipped contig. The consensus of the inner sequences can be computed more quickly using the Spoa library, which implements a partial order alignment algorithm [46]. These two improvements coupled with an appropriate ultra-long Nanopore read coverage (10X) could lead to a hybrid assembly pipeline that is superior to the current long-range mate-pair technologies where these improvements are not possible due to the fact that, in both technologies, the gap sequence between pairs is unknown.

Clearly, improvement in the base accuracy of long reads will increase the recall of Fast-SG and thus impact positively on the hybrid assembly process. Notice, however, that read recall is less important because not all of the sequenced reads are useful for scaffolding. Indeed, we showed with the Illumina scaffolding benchmarks that the short-read aligners with higher read recall produced the worst scaffolding results. Additionally, Fast-SG was designed to enable construction of the scaffolding graph from uniquely mapped read pairs (Fast-SG index). It thus discards any repetitive sequences as they are not useful for building the scaffolding graph. ONT is a fast-evolving technology, and current use of the new 1*D*^2^ chemistry or improvement in the base callers are two alternatives that could lead to an increased base accuracy of the ONT reads.

## Conclusions

Here, we introduced a new method, Fast-SG, that enables the construction of a scaffolding graph from either short or long reads, allowing for accurate construction of the scaffold sequences as well as for software reuse.

We showed that near-perfect synthetic libraries are obtained with Fast-SG from either corrected or uncorrected PacBio and Nanopore long reads. The insert size is restricted to the actual long-read size. However, using ultralong Nanopore reads,Fast-SG is able to extract synthetic libraries of even bacterial artificial chromosome clone sizes with insert sizes of 150–180 kb. Those kinds of libraries were crucial to reach the high continuity of the current human reference genome [40]. An estimation of the gap size with the existing long-range mate-pair technologies (10X genomics and DOVETAIL genomics) is more challenging than with the synthetic libraries due to the fact that in such technologies, the linking information comes from a range of insert sizes and the relative orientation of the read pairs may not be known (DOVETAIL genomics).

Clearly, the synthetic libraries eliminate the bottleneck of sequencing a combination of mate-pair libraries, which were typically required to obtain long-range assemblies [2, 23, 24]. We further showed that short-read scaffolders are able to produce accurate scaffolds when they are fed with the synthetic libraries extracted by Fast-SG, thus leading to results that are superior to or match those obtained by Links, a scaffolder specifically designed for hybrid long-read scaffolding. Futhermore, we showed that Fast-SG is faster than the current state-of-the-art short-read aligners and that better results are achieved by the scaffolding tools when they are coupled with Fast-SG on illumina mate-pair data.

Finally, we demonstrated that Fast-SG in conjunction with efficient algorithms designed for Illumina data can be used to perform a full hybrid assembly of large genomes. The resulting assemblies are superior or comparable to the current state-of-the-art long-read assembly pipelines. Additionally, the modular hybrid pipelines are faster and remarkably efficient at shallow long-read coverage (5X–10X). The scalability to large genomes, moderate computational resources, and the shallow long-read coverage required by the proposed solution represent significant improvements over the current hybrid assembly methods.

Overall, we believe that Fast-SG opens the door to achieve accurate hybrid long-range reconstructions of large genomes with low effort, high portability, and low cost.

## Availability of supporting data

Code snapshots and test data for demonstration of sequence assembly tools are available in the GigaScience GigaDB repository [47].

## Availability and requirements

- Project name: Fast-SG
- RRID (Research Resource Identification Initiative ID) : SCR_015934
- Project home page: https://github.com/adigenova/fast-sg
- Operating system(s): Unix, Linux and Mac OSX
- Programming language: C++ and PERL
- Other requirements: Compilation was tested with g++ version 5.3 (Linux) and clang version 4.2 (Mac OSX)
- License: MIT
- Any restrictions to use by non-academics: none

## Additional files

**Additional file 1:** The Supplementary-Material.pdf file contains the following Sections, Tables and Figures: **Sections**: Supplementary Material 1: *Software and datasets*. Supplementary Material 2: *Long read scaffolding benchmark*. Supplementary Material 3: *Arabidopsis thaliana (Ler-0) and human (NA12878) hybrid genome assemblies*. Supplementary Material 4: *Illumina alignment benchmark*. Supplementary Material 5: *Illumina scaffolding benchmark*. **Tables**: Supplementary Table S8 : Fast-SG*recall at k-mer and read level on synthetic mate-pair libraries extracted from corrected or uncorrected long reads using the E. coli K12 dataset*. Supplementary Table S9 : *Long read datasets used for comparison against*Links. Supplementary Table S10: *Number of k-mer pairs and read pairs extracted from raw long reads by*Links*and*Fast-SG. Supplementary Table S11 : *Long read scaffolding benchmark results for E. coli K12 and S. cerevisiae W303*. Supplementary Table 12 : *Number of synthetic read pairs aligned to the*DiscovarDeNovo*assembly of Arabidopsis thaliana (Ler-0)*. Supplementary Table 13 : *Number of synthetic read pairs aligned to the human (NA12878)*DiscovarDeNovo*assembly*. Supplementary Table S14 : *Example (blue rows) of short contig skipped in chromosome 6*. Supplementary Table S15 : *Example (blue rows) of chimeric contigs in chromosome 6 from the*Canu*and*MaSuRCA*assemblies*. Supplementary Table S16 : Short read alignment benchmark. **Figures**: Supplementary Fig. S1: *Boxplot of synthetic libraries extracted by*Fast-SG*(K21) from the PacBio reads to scaffold the Arabidopsis thaliana (Ler-0) genome*. Supplementary Fig. S2: *Boxplot of synthetic libraries extracted by*Fast-SG*(K22) from the ONT ultra-long reads to scaffold the human (NA12878) genome*. Supplementary Fig. S3: *Amount of bases involved in structural errors by type in the Arabidopsis thaliana (Ler-0) assemblies*. Supplementary Fig. S4: Nucmer*plots of the human (NA12878) assemblies*. Supplementary Fig. S5: *Percentage of paired-end reads aligned by*Fast-SG*and the short read aligners for each Illumina dataset*. Supplementary Fig. S6: *Pairwise contig read coverage correlation between the short read aligners and*Fast-SG. (PDF 2.5 Mb).

**Additional file 2:** The Supplementary_Table_S17.xlsx file contains the Illumina scaffolding benchmark results for *S. aureus* using the ScaffMatch, Opera-LG, Besst2 and Boss scaffolders. (XLSX 56 Kb).

**Additional file 3:** The Supplementary_Table_S18.xlsx file contains the Illumina scaffolding benchmark results for *R. sphaeroides* using the ScaffMatch, Opera-LG, Besst2 and Boss scaffolders. (XLSX 85 Kb).

**Additional file 4:** The Supplementary_Table_S19.xlsx file contains the Illumina scaffolding benchmark results for *P. falciparum* using the ScaffMatch, Opera-LG, Besst2 and Boss scaffolders. (XLSX 132 Kb).

**Additional file 5:** The Supplementary_Table_S20.xlsx file contains the Illumina scaffolding benchmark results for *H. sapiens* using the ScaffMatch, Opera-LG, Besst2 and Boss scaffolders. (XLSX 101 Kb).

## Abbreviations

CPU: central processing unit; DFS: DiscovarDeNovo+Fast-SG+ScaffMatch; MPHF: minimal perfect hash function; ONT: Oxford Nanopore; PacBio: Pacific Biosciences; PD: probabilistic dictionary; SAM: sequence alignment/map; BAC: bacterial artificial chromosome; PCR: polymerase chain reaction; SRA: Sequence Read Archive; ENA: European Nucleotide Archive.

## Competing interests

The authors declare that they have no competing interests.

## Funding

This work was supported by the Conicyt-PIA Concurso AFB 170001, Fondap 150900007, MAIA STIC-AmSud and CONICYT PFCHA/BECA DOCTORADO NACIONAL 2014/FOLIO 21140124 granted to A.D.G. This research was partially supported by the supercomputing infrastructure of the NLHPC (ECM-02) and ANR-16-CE23-0001 (ASTER) project.

## Author Contributions

M.F.S. proposed the scaffolding problem. A.D.G. devised the original ideas for Fast-SG. A.D.G. developed, implemented, and tested Fast-SG. M.F.S., G.R.A., and A.M. provided helpful discussions and guidance for the project. A.D.G. wrote the initial version of the manuscript. M.F.S. improved the initial version of the manuscript. M.F.S., G.R.A., and A.M. revised the manuscript. All authors read the paper and approved the final version.

## Supplementary Material

GIGA-D-17-00335_Original_Submission.pdf

GIGA-D-17-00335_Revision-1.pdf

GIGA-D-17-00335_Revision-2.pdf

Response-to-Reviewer-Comments_Original-Submission.pdf

Response-to-Reviewer-Comments_Revision-1.pdf

Reviewer-1-Report-(Original-Submission) -- Sagar Utturkar 21 Dec 2017 Reviewed

Reviewer-1-Report-(Revision-1) -- Sagar Utturkar 15 Mar 2018 Reviewed

Reviewer-2-Report-(Original-Submission) -- Daniela Puiu 25 Dec 2017 Reviewed

Supplement materials
