# Supplementary material for: Fast-SG: an alignment-free algorithm for hybrid assembly
Source: Gigascience. 2018 May 5;7(5):giy048. doi: 10.1093/gigascience/giy048 (PMC6007556; doi:10.1093/gigascience/giy048)
Supplement: GIGA-D-17-00335_Revision-2.pdf [file giy048_giga-d-17-00335_revision-2.pdf]

GigaScience

Fast-SG: An alignment-free algorithm for hybrid assembly

--Manuscript Draft--

**Manuscript Number:**	GIGA-D-17-00335R2
**Full Title:**	Fast-SG: An alignment-free algorithm for hybrid assembly
**Article Type:**	Technical Note
**Funding Information:**	CONICYT (CONICYT PFCHA/BECA DOCTORADO NACIONAL 2014/FOLIO 21140124) — Dr Alex Di Genova<br>Basal program (PFB03) — Not applicable
**Abstract:**	Background: Long read sequencing technologies are the ultimate solution for genome repeats, allowing near reference level reconstructions of large genomes. However, long read de novo assembly pipelines are computationally intense and require a considerable amount of coverage, thereby hindering their broad application to the assembly of large genomes. Alternatively, hybrid assembly methods which combine short and long read sequencing technologies can reduce the time and cost required to produce de novo assemblies of large genomes.<br>Results: In this paper, we propose a new method, called FAST-SG, which uses a new ultra-fast alignment-free algorithm specifically designed for constructing a scaffolding graph using light-weight data structures. FAST-SG can construct the graph from either short or long reads. This allows the reuse of efficient algorithms designed for short read data and permits the definition of novel modular hybrid assembly pipelines. Using comprehensive standard datasets and benchmarks, we show how FAST-SG outperforms the state-of-the-art short read aligners when building the scaffolding graph, and can be used to extract linking information from either raw or error-corrected long reads. We also show how a hybrid assembly approach using FAST-SG with shallow long read coverage (5X) and moderate computational resources can produce long-range and accurate reconstructions of the genomes of Arabidopsis thaliana (Ler-0) and human (NA12878). Conclusions: FAST-SG opens a door to achieve accurate hybrid long-range reconstructions of large genomes with low effort, high portability and low cost.
**Corresponding Author:**	Alejandro Maass, Ph.D<br>Universidad de Chile, Facultad de Ciencias Fisicas y Matematicas<br>CHILE
**Corresponding Author Secondary Information:**	
**Corresponding Author's Institution:**	Universidad de Chile, Facultad de Ciencias Fisicas y Matematicas
**Corresponding Author's Secondary Institution:**	
**First Author:**	Alex Di Genova, Ph.D
**First Author Secondary Information:**	
**Order of Authors:**	Alex Di Genova, Ph.D
	Gonzalo A Ruz, Ph.D
	Marie-France Sagot, Ph.D
	Alejandro Maass, Ph.D
**Order of Authors Secondary Information:**	
**Response to Reviewers:**	GIGA-D-17-00335R1<br>Fast-SG: An alignment-free algorithm for hybrid assembly<br>Alex Di Genova, Ph.D; Gonzalo A Ruz, Ph.D; Marie-France Sagot, Ph.D; Alejandro Maass, Ph.D<br>GigaScience

Powered by Editorial Manager® and ProduXion Manager® from Aries Systems Corporation

	Dear Dr. Edmunds,<br><br>We are resubmitting a revised manuscript of "FAST-SG: An alignment-free algorithm for hybrid assembly". We have carefully considered all the minor points raised by the reviewers and editors.<br>Our point-by-point answers to the editor's and to the reviewers' minor points are the following:<br><br>Point raised by the Editor:<br><br>Your manuscript "Fast-SG: An alignment-free algorithm for hybrid assembly" (GIGA-D-17-00335R1) has been assessed by our reviewers. Based on these reports, and my own assessment as Editor, I am pleased to inform you that it is potentially acceptable for publication in GigaScience, once you have carried out some very minor revisions suggested by our reviewers and have also created a snapshot of the code and any test data in our GigaDB repository that you will need to cite in an "Availability of supporting data" section. I've cc'd our curators here and they can help you put this together.<br><br>A: We have followed the Editor's suggestion and we deposited a snapshot of the Fast-SG code as well as all the datasets and results described within the manuscript in the GigaDB repository. Currently, the GigaDB page describing this information is the following:<br><br>http://gigadb.org/dataset/view/id/100437/token/hyKuNQtlVs0v5R2D<br><br>The GigaDB curators on 16/04/2018 should finish the page and provide the final link to the Fast-SG repository.<br><br><br>Points raised by Reviewer #1:<br><br>Reviewer #1: Thank you for addressing my comments to satisfaction. Addition of "Procedure for effective hybrid assembly with FAST-SG" section would be very useful for the users.<br><br>Suggestion:<br>I think that Pie-chart representation of scaffolding errors (in previous version) appears neat as compared to current bar-chart. I only requested a minor change to convert the percentage values in pie chart to real numbers. Sometimes, percentage values in pie charts could be a misleading comparison because same percentage value in two pie chart could represent very different real numbers.<br><br>Authors can decide which figure to include in the manuscript and I have no reservation with that decision.<br><br>A: We followed the Reviewer's suggestion and we went back to the pie chart figure. We do thank again the Reviewer for this positive overview of our work.
**Additional Information:**	
**Question**	**Response**
Are you submitting this manuscript to a special series or article collection?	No
**Experimental design and statistics**<br><br><br>Full details of the experimental design and statistical methods used should be given in the Methods section, as detailed in our Minimum Standards Reporting Checklist. Information essential to interpreting the data presented should be made available	Yes

in the figure legends.<br><br>Have you included all the information requested in your manuscript?	
**Resources**<br><br>A description of all resources used, including antibodies, cell lines, animals and software tools, with enough information to allow them to be uniquely identified, should be included in the Methods section. Authors are strongly encouraged to cite Research Resource Identifiers (RRIDs) for antibodies, model organisms and tools, where possible.<br><br>Have you included the information requested as detailed in our Minimum Standards Reporting Checklist?	Yes
**Availability of data and materials**<br><br>All datasets and code on which the conclusions of the paper rely must be either included in your submission or deposited in publicly available repositories (where available and ethically appropriate), referencing such data using a unique identifier in the references and in the “Availability of Data and Materials” section of your manuscript.<br><br>Have you have met the above requirement as detailed in our Minimum Standards Reporting Checklist?	Yes

Powered by Editorial Manager® and ProduXion Manager® from Aries Systems Corporation

Manuscript

Click here to download Manuscript Fast-SG-GigaScience-V08.tex

GigaScience, 2017, 1–20

doi: xx.xxxx/xxxx
Manuscript in Preparation
Technical note

TECHNICAL NOTE

Fast-SG: An alignment-free algorithm for hybrid assembly

Alex Di Genova[1,2,3,4,5], Gonzalo A. Ruz[1,6], Marie-France Sagot[3,4,†] and Alejandro Maass[2,5,7,†]

[1]Facultad de Ingeniería y Ciencias, Universidad Adolfo Ibáñez, Santiago, Chile. and [2]Mathomics Bioinformatics Laboratory, Center for Mathematical Modeling, University of Chile, Av. Blanco Encalada 2120, 7th floor, Santiago, Chile. and [3]Inria Grenoble Rhône-Alpes, 655, Avenue de l'Europe, 38334 Montbonnot, France. and [4]CNRS, UMR5558, Université Claude Bernard Lyon 1, 43, Boulevard du 11 Novembre 1918, 69622 Villeurbanne, France. and [5]Fondap Center for Genome Regulation, Av. Blanco Encalada 2085, 3rd floor, Santiago, Chile. and [6]Center of Applied Ecology and Sustainability (CAPES), Santiago, Chile. and [7]Department of Mathematical Engineering, University of Chile, Av. Blanco Encalada 2120, 5th floor, Santiago, Chile.

[†] To whom correspondence should be addressed: Marie-France Sagot – marie-france.sagot@inria.fr and Alejandro Maass – amaass@dim.uchile.cl

Abstract

Background: Long read sequencing technologies are the ultimate solution for genome repeats, allowing near reference level reconstructions of large genomes. However, long read *de novo* assembly pipelines are computationally intense and require a considerable amount of coverage, thereby hindering their broad application to the assembly of large genomes. Alternatively, hybrid assembly methods which combine short and long read sequencing technologies can reduce the time and cost required to produce *de novo* assemblies of large genomes.
Results: In this paper, we propose a new method, called FAST-SG, which uses a new ultra-fast alignment-free algorithm specifically designed for constructing a scaffolding graph using light-weight data structures. FAST-SG can construct the graph from either short or long reads. This allows the reuse of efficient algorithms designed for short read data and permits the definition of novel modular hybrid assembly pipelines. Using comprehensive standard datasets and benchmarks, we show how FAST-SG outperforms the state-of-the-art short read aligners when building the scaffolding graph, and can be used to extract linking information from either raw or error-corrected long reads. We also show how a hybrid assembly approach using FAST-SG with shallow long read coverage (5X) and moderate computational resources can produce long-range and accurate reconstructions of the genomes of *Arabidopsis thaliana* (Ler-0) and human (NA12878).
Conclusions: FAST-SG opens a door to achieve accurate hybrid long-range reconstructions of large genomes with low effort, high portability and low cost.

Key words: Hybrid assembly; Genome scaffolding; Alignment-free;

Compiled on: April 13, 2018.
Draft manuscript prepared by the author.

Findings

Background

The major challenge of whole genome *de novo* assembly is to solve repeats [1, 2]. These correspond to nearly identical genomic sequences that occur at multiple locations in a genome. To address this challenge, two major types of approaches have been proposed, one using paired short reads [3] and the other long reads [4].

In the second case, the aim is to hopefully entirely capture the repeats within the long reads. The non repeated suffix and prefix sequences of such long reads are used to compute unique overlaps, which then allow to unambiguously expand the original reads into larger ones, called contigs, in a process that may sometimes (but not always) directly lead to inferring the entire genomic sequence.

The first type of approach instead needs to be associated to an operation called genome scaffolding. The short reads are still first assembled into contigs as above, either by also computing overlaps [5] or by using de Bruijn graphs [6]. The contigs obtained in this case will however not span the whole genome. Indeed, most often they will be much shorter. They then need to be joined (*i.e.* linked together) in a second step. The linking information is in general provided by paired-end or mate-pair sequencing. Commonly, genomic fragments larger than 1kb from which both ends are sequenced are denoted as mate-pair libraries, otherwise they are referred to in the literature as pair-end libraries. Genome scaffolding that uses paired short reads introduce gaps (*i.e.* unknown sequences) between the contigs, thereby once again not leading to the entire genomic sequence but to a set of so-called scaffold sequences, or scaffolds for short. A scaffold thus represents a set of ordered and oriented contigs.

The genome scaffolding problem was first formulated by Huson *et al.* [7]. The method proposed by the authors started by building what is called a scaffolding graph where the nodes represent the contigs and the edges encode the number of mate-pairs (weight), the orientation and the distance between two different contigs. A greedy algorithm is then used to heuristically obtain optimal paths that will correspond to the scaffold sequences.

Most of the scaffolding methods that have been developed since use the same type of graph, built with ultra-fast short-read aligners [8, 9, 10] as a foundation for the scaffolding [3]. Algorithmic innovations in the area are mainly focused on how to select optimal paths (usually those of maximal weight) and thus obtain large and accurate scaffolds. Various approaches have been proposed, based on dynamic programing [11], breadth-first search [12], maximum weight matching [13], or branch and bound [14], among others.

The new long read sequencing technologies (Pacific Biosciences, Oxford Nanopore) suddenly changed the genome assembly scene by producing very long (>10kb) reads that however contain a high level of errors (on average 15% at the current

time). These new technologies nevertheless extended the landscape of solvable repeat sequences [15]. Currently, *de novo* assemblers that use such long reads [4, 16] are thus able to finish bacterial genomes and to produce highly continuous reconstructions of human genomes [4, 17]. However, *de novo* assemblies of large genomes based on computing overlaps [5] are computationally intense [4] and require a considerable amount of coverage (50X) to error-correct the inaccurate long read sequences by self-correction methods, thereby hindering a broad application of these methods to the *de novo* assembly of large genomes [17].

De novo assemblies using long reads have nevertheless proven to be scalable to chromosomes [18, 19] when associated with complementary long range information from novel library preparation techniques [20, 21]. Such new experimental libraries are sequenced on Illumina machines leading to conventional paired-end reads. DOVETAIL genomics [20] thus produces useful linking information in the range of 1-200kb, while 10X genomics [22] produces, by using barcodes in a clever manner, linked-reads in the range of up to 100kb. Both technologies then use such long-range information within their assembly pipelines [20, 22] to build a scaffolding graph to which they apply their own algorithmic solutions to obtain the scaffold sequences. Both technologies were

conceived with the aim of replacing the expensive and time consuming experimental protocols required to produce long-range mate-pair libraries [23, 24] with short-read sequencing.

In principle, long-range information can be extracted directly from long reads in ranges restricted to the latter's actual sizes. Such information can then be used to devise a hybrid assembly method, where high quality contigs from short read assemblies are used as nodes of the scaffolding graph, edges are created using linking information from the long reads, and the scaffolds are generated by a short read scaffolder. However, there is currently a lack of algorithms for building a scaffolding graph from the long reads. Such algorithm would allow the reuse of efficient existing short-read algorithms to compose novel hybrid assembly pipelines.

Being able to (i) build such a graph from either short or long reads in an ultra-fast way, with moderate computational resources, while (ii) keeping the structure standard enough to be compatible with the existing efficient short read scaffolders are the main challenges that we address in this paper. The method that we propose, called FAST-SG, uses an alignment-free algorithm [25] strategy as well as information from varied sequence sources (Illumina, Pacific Biosciences and Oxford Nanopore), and was conceived to maximize scalability, speed, and modularity. The latter characteristic in particular allows to define novel hybrid assembly pipelines, which permits the efficient assembly of large genomes.

FAST-SG was extensively tested using a comprehensive set of standard datasets [3, 26] and benchmarks. We show that FAST-SG enables the hybrid assembly of large genomes and is especially effective with shallow long read coverage data (5X-10X). Our hybrid strategy consists in the construction of several synthetic mate-pair libraries which could have insert size up to BACs (180kb) and can be combined with a short read scaffolder to generate long-range scaffolds. Such strategy scales to human size genomes with moderate computational resources. Moreover, we show that FAST-SG is faster (7-15X) than classic short read aligners and is a powerful alternative for scaffolding with short mate-pair data.

We conclude by providing a procedure for an effective hybrid assembly with FAST-SG and we discuss how the strategy that we propose can be extended to use long reads to fill the gaps and error-correct the scaffold sequences.

Algorithm

FAST-SG index

The FAST-SG index consists of all the unique k-mers present in the set of target contigs at a given k-mer length. For each of them, we store the position, the strand and the contig of origin, using lightweight data structures such as Minimal Perfect Hashing [27] and Probabilistic Dictionary [28]. In a first step, we define the unique k-mers as being those having a frequency equal to 1 from the total set of distinct k-mers present in the target contig/genome sequences. To identify unique k-mers, we use KMC3 [29], an ultrafast, parallel and memory frugal k-mer counter. In a second step, each unique k-mer is hashed to the space of $[2^0, 2^{64}]$ using a rolling hash function [30] and with hash values written on the fly to a binary file. Rolling hashing has the nice property of computing hash values for consecutive k-mers in a sequence in $\mathcal{O}(k + l)$ time, where k is the k-mer length, l is the sequence length, and $k < l$. We use an efficient library implementation of rolling hash algorithms called NTHASH [31], which implements a barrel shift function and a seed table of integers to compute hash values in both DNA strands faster.

In a third step, the static hash values stored in the binary file are used as input to create a Minimal Perfect Hash Function (henceforth denoted by MPHF). MPHF provides a collision-free and minimal space way to store and look-up hash values in constant worst-case access time for static sets. We use the library implementation provided by Limasset *et al.* [27], called BBHASH, which is simple, parallel, fast and memory frugal. Moreover, it can store 10^{10} hash values using moderate computational resources (5Gb). The major feature of MPHF is its ability to map each key of S (in our case, the unique k-mer hashed values) to an integer in the interval $[1, N]$ (injective function), with $N = |S|$, while avoiding the implicit storage of hash values by using cascade hash functions

in conjunction with bit vectors. A significant parameter of BBHASH is the γ (gamma) factor. We use a γ factor equal to 4, which is an optimal value for fast query time, fast construction and low memory usage [27]. When performing a query in the MPHF structure, it returns an index in the interval of $[1, N]$ which has the same size of the static set S, allowing to store related data for each $s \in S$ using simple arrays. If we query a key not present in the initial static set S, MPHF could return a value in the interval $[1, N]$ that is a false positive [28].

In a fourth step, to control the false positive rate (p) of MPHF, we use a probabilistic set [28]. For each indexed element $s \in S$ (unique k-mers), we store a fingerprint value using 16 bits in an array of size $N = |S|$ at the corresponding MPHF index of s. The fingerprint is built by re-hashing the hash value of s using the xor-shift hash function in the range $[2^0, 2^{16}]$ and storing it in a bit-set array structure. We selected a fingerprint of size 16 bits, because it has a low false positive rate $p = 1/2^{16} = 0.0000152$.

Finally, we added the associated contig_id, strand and coordinate values of each unique k-mer stored in the MPHF and the probabilistic dictionary (MPHF-PD), by performing a single pass through the set of contigs/genome sequences, using the same k-mer size. For each k-mer hit, we store the values (contig_id, coordinate and strand) in the index returned by the MPHF-PD structure using three vectors having the same size as the set S. After storing all the associated values, we end our index construction and return a reference to the new object. This object is the FAST-SG index. The memory required per k-mer is composed of 6 bits for the MPHF, 16 bits for the probabilistic dictionary, 32 bits for the contig_id, 32 bits for the position, and 1 bit for the strand, adding to a total memory of 87 bits.

FAST-SG alignment-free method

The core of FAST-SG is an alignment-free algorithm specifically designed to construct the scaffolding graph from either short or long reads using light-weight data structures. Such graphs are built using as information the read pairs that map uniquely to different contigs. If the mappings are within an expected distance from one another given the respective orientation of the reads, an edge is added to the graph between the contigs [3]. The uniqueness property of the mapping is ensured by its high quality score which represents the confidence that the read indeed belongs to the reported genomic location [9, 10]. When a read belongs to two possible genomic locations, a score of 0 is commonly assigned.

Current short read aligners identify the high quality score mappings by indexing all the k-mers present in the set of contigs and using a seed-and-extend [9, 10] alignment approach. Instead, in FAST-SG, only the k-mers having a frequency equal to 1 are considered and no alignment is performed. After building the FAST-SG index, the contig location for a pair of reads is determined following a number of steps as illustrated in Figure 1a.

The first step performs look-ups of the k-mers of the forward (resp. reverse) read sequence (on both strands by using a rolling hash function) in the FAST-SG index, and fills a vector of hits of a predefined size. The size of the vector depends on the error rate of the sequencing technology. The default chosen in FAST-SG is of 10 for Illumina and 20 for the long-read technologies. In a second step, the forward (resp. reverse) vector of the k-mer hits is sorted by contig and, inside each contig, by coordinate. In the third step, a score is computed for the forward (resp. reverse) read that corresponds to the maximum number of hits falling inside a window of size equal to the length of the read. If the score of both reads in a pair reaches a predefined minimum, in a fourth step the genomic location of the pair is reported. Otherwise, a pair rescue is attempted (fifth step) by fixing the location of the best scored read and looking for a k-mer hit in the mate-pair that satisfies the expected distance and orientation (Figure 1a).

A major parameter of the algorithm is the k-mer size as this governs the number of unique k-mers to be indexed in a given genome, or in our case, a set of contigs. In Figure 1b, we show how the number of unique k-mers increases as a function of the k-mer size in the human genome (GRCh38.p10). However, large k-mers need reads with low error rates for a successful match. To define an appropriate k-mer size, it is necessary to take into account both the error rate and the length of the query sequence. Almost all short read aligners use as seeds short k-mers (15-32 base pairs) because they have a low probability of containing

errors and provide enough specificity [32, 9, 10]. Additionally, the available long read algorithms such as CANU [4], LORDEC [33], and MASURCA [34] among others, employ short *k*-mers (15-19 base pairs) at some stages to deal with the large error rates (15%) present in the current long read technologies. In practice, FAST-SG supports a *k*-mer size of up to 256 base pairs, but for the Illumina reads, values of k between 15 and 80 were tested while for long reads, these ranged from 15 to 22 base pairs,

which according to our benchmarks provide enough specificity, even for large genomes (Figure 1b). There are for instance 1.83 billion unique 19-mers (Figure 1b) in the human genome, which is a good approximation of the non-repetitive regions for this genome [2].

Another issue of working with *k*-mers is the memory required for storing them for fast look-ups. This was addressed by implementing a novel probabilistic data structure (FAST-SG index) which only requires 87 bits per *k*-mer, while memory increases as a function of the number of unique *k*-mers to store (Figure 1c). To index in memory all the unique *k*-mers of the human genome at a given *k*-mer size (<256bp) therefore requires less than 30Gb of memory (Figure 1c).

Finally, the genomic location of the read pairs is reported using a single representative unique *k*-mer for each read in SAM format [35], thus allowing for an easy integration with scaffolders that support this standard format. The steps of scoring and pair rescuing follow some of the ideas used in the SSAHA [32] and BWA-MEM [36] aligners.

Illumina mate-pair reads alignment

Illumina mate-pair reads are aligned using the algorithm described previously (FAST-SG alignment-free strategy). The forward read (QF) is iterated *k*-mer by *k*-mer where for each *k*-mer, we ask if it is present in the FAST-SG index until 10 hits are stored in the vector *vectorFUH*. If the score of QF is larger than 3, we attempt to fill the vector *vectorRUH* (QR) of the reverse read. Then, if the score of each read is larger than 5, the positions are reported. Otherwise, we attempt pair rescue by fixing the position of the best-scored read and requiring a minimum score of 4 for the rescued read. These parameters of minimum and pair-rescue scores were set from empirically derived defaults. Such default short read parameters can be modified by the user.

Extraction of synthetic pairs from long reads

Synthetic pairs of reads (QF and QR) are extracted from the long-read sequences having a default read length of 200 base pairs in forward-reverse orientation and separated by a distance *D* (insert size). Multiples values of *D* can be specified to comprehensively extract linking information from the long reads. After extracting a synthetic pair, each query sequence (QF and QR) is aligned using the algorithm described previously (FAST-SG alignment-free strategy). A minimum score of 15 and a minimum rescue score of 4 are used as default parameters. Then, as default, a moving window of 100bp is adopted to extract another pair, until the complete long read sequence is scanned. The default long read parameters can be modified by the user.

Estimation of the genomic library parameters

The genomic library parameters for insert size, standard deviation and orientation are estimated using a subset of the mate-pair sequences in order to use them in the rescue step of FAST-SG. These subsets of mate-pair reads are aligned to the target contigs/genomes, and the read pairs located within contigs are used to estimate the library parameters. For Illumina, we use a total of 100,000 pairs which are aligned to the target sequences using a minimum score of 8 and without pair rescue. Then, for each aligned pair within contigs, we save the pair orientation and distance. To infer the average insert size and standard deviation, we remove 10 percent outliers from both tails of the values stored by sorting the observed insert sizes by increasing order. The orientation is computed using a majority rule on the four possible orientations for a pair of reads (FR, RR, FF, RF). For long reads, we use a total of 1,000 long read sequences and we extract the specified insert sizes to infer the average insert size and standard deviation as for the Illumina reads. The orientation for the synthetic libraries is not estimated because all pairs are created in

forward-reverse orientation.

Concurrent steps of Fast-SG

The index construction and alignment steps in Fast-SG are concurrent. The Fast-SG index can use multiple threads to construct the MPHF [27] and store the associated *k*-mer information (contig_id, coordinate, strand). Chunks of 5Mb of contig sequences are used to populate in parallel the Fast-SG index. The Fast-SG alignment step is concurrent by taking chunks of 500,000 and 1,000 for the short and long reads respectively. The concurrent steps are implemented using the Pthread library. The user specifies the number of CPUs to be used.

Data Description

Datasets and software

We collected a comprehensive collection of standard datasets (Table 1) which are frequently used to benchmark the new sequencing technologies, scaffolding tools or genome assembly pipelines.

Long read datasets were used to investigate the capacity of Fast-SG to extract linking information from long reads, and then the performance of short read scaffolders fed with Fast-SG when compared to a dedicated long read scaffolder. In the first case, the genome of *Escherichia coli K.12* was adopted as it have been sequenced by multiple long read technologies and is commonly used to validate the long read algorithms [4]. In the second case, both *E. coli K12* and *Saccharomices cerevisiae* W303 (Table 1) were employed to prove that short read scaffolders can use synthetic mate-pair libraries extracted from long reads.

To explore the amount of long read coverage required by the hybrid solutions, we compared the performance of the latter to the results obtained by Canu [4], a state-of-the-art long read assembler. In a first step, we used the genome of *Arabidopsis thaliana*, and then in a second step, a complete human genome (NA12878, Table 1). NA12878 was selected because it was sequenced on a variety of platforms [17, 22, 20, 37] and assembled by a variety of algorithms [4, 34, 22, 20]. It thus allows to compare the complete landscape of currently available long-range technologies and assembly pipelines.

To assess the performance of Fast-SG for constructing the scaffolding graph from short reads, we employed all the short read datasets and Illumina assemblies defined in Hunt *et al.* [3]. These short read datasets include the genomes of *Staphylococcus aureus*, *Rhodobacter sphaeroides*, *Plasmodium falciparum* and the human chromosome 14 (Table 1), and are commonly used as the gold standard for validation of the scaffolding tools [14, 13, 12, 11].

We coupled Fast-SG with two well established scaffolders, namely Opera-LG [11] and Besst2 [12], and two more recently published ones, namely ScaffMatch [13] and Boss [14], to produce scaffold sequences from short or long read data. All the chosen scaffolders have different algorithms to select optimal paths from the scaffolding graph and use the Sam/Bam format as input. Besst2 was excluded from the hybrid scaffolding experiments due to an exception produced while Besst2 computes the average contig coverage from synthetic mate-pair libraries. All the software and reference genomes used are described in the Supplementary Material 1.

Short and long reads benchmarks

All scaffold sequences generated from alignments produced by Fast-SG, by the short read aligners, and by Links were evaluated following the standard defined by Hunt *et al.* [3]. For each dataset, the true contig layout is known and the scaffold sequences were compared against it in order to determine the following scaffolding errors (represented as a bitwise flag):

0 = Correct pair of contigs.

1 = Contigs originated from same reference sequence, but their orientation in the scaffolds is incorrect.

2 = Contigs originated from different reference sequences.

4 = Contigs originated from the same reference sequence, but are the wrong distance apart.

5 = **4+1**, Contigs originated from same reference sequence, but their orientation and distance in the scaffold are incorrect.

8 = Contigs originated from the same reference sequence, but are not in the correct order.

12 = **8+4**, Contigs originated from the same reference sequence, but are not in the correct order and distance.

From the previous values, we computed the F-Score metric, which was first introduced by Mandric and Zelikovsky [13], and adopted in Luo *et al.* [14] also with the purpose of improving and summarising in a single metric the performance of a scaffolding tool. In brief, if we denote by P the number of potential joins that can be made, TP the number of correct joins performed by a scaffolder (true positives), and FP the number of wrong joins (false positives), we can calculate the following quality metrics:

$$Recall = \frac{TP}{P}$$

$$Precision = \frac{TP}{(TP+FP)}$$

$$F - Score = 2\,\frac{(Recall * Precision)}{(Recall + Precision)}$$

The structural quality of the hybrid and *de novo* assemblies was determined via direct comparison against the nearest reference genomes available using NUCMER [38] and reported using the GAGE statistics [26] which from 1-to-1 alignments evaluates both the identity and the structural breakpoints (inversions, relocations and translocations). All commands executed in each benchmark are specified in the Supplementary Materials 2 to 5.

Results

Extracting synthetic mate-pair libraries from long reads

Despite the high per-base error rate of the long reads technologies, the long-range information encoded in a long read has proven to be highly accurate. On the other hand, current experimental protocols to produce long-range mate-pair libraries using short read technologies are time-consuming and expensive [23, 24]. Moreover, library contamination occurs when the circularization step fails during construction, resulting in mate-pairs with short insert size and in the wrong orientation [12]. Extracting synthetic mate-pair libraries directly from long reads could improve the performance of the current short read scaffolders and replace the need for sequencing multiple mate-pair libraries for scaffolding.

To demonstrate the utility of FAST-SG to create synthetic mate-pair libraries from long reads, we collected the latest chemistry data sequenced with the Oxford Nanopore (1D reads sequenced on R9.2 flow cells) and Pacific Biosciences (Sequel System) technologies, respectively denoted by ONT and PacBio from now on, for the genome of *Escherichia coli* K12 (Table 1). The long reads were error-corrected using Illumina reads (Supplementary Material 2) with LORDEC [33], a hybrid error-correction method.

FAST-SG was used to generate synthetic mate-pair libraries in the range of 0.5-8kb from the corrected and uncorrected long reads using a k-mer size of 15, at which 98% of the k-mers are unique in the reference *E. coli* K12 genome. Synthetic mate-pair reads were aligned to an Illumina assembly of *E. coli K12* (Table 1). Near perfect synthetic mate-pair libraries were obtained with a low percentage of outliers (<9.85%) for all insert sizes (Figure 2). Moreover, the hybrid error-correction reduced the standard deviation and allowed the average insert size to get close to the specified size of each synthetic library. However, the hybrid error-correction increased the number of outliers in both technologies (Figure 2). The observed average insert size (Figure 2) in the synthetic libraries from ONT are slightly higher than the observed ones in PacBio, thus reflecting the nature of the error of each long read technology, which are deletions for ONT [4] and substitutions for PacBio [4].

We computed the recall achieved by FAST-SG at the levels of the k-mers and of the synthetic mate-pair reads (the length of the forward and reverse reads equals 200 base pairs) for each long read technology, from either raw or corrected reads (Supplementary Table S8). At the k-mer level, FAST-SG has a recall of 8.3% and 5.05% for the uncorrected reads of ONT and PacBio, respectively. The hybrid error-correction increased the k-mer recall by 10% for both long read technologies. At the synthetic mate-pair read level, we observed a recall of 49.42% and 31.65% for the raw ONT and raw PacBio reads, respectively. The hybrid error-correction increases the synthetic mate-pair read recall for ONT to 75.12% and for PacBio to 65.02%. We observed that FAST-SG is more effective aligning synthetic mate-pair reads from raw ONT than from raw PacBio reads. We expect that this is due to the nature of the ONT errors (major deletions) as FAST-SG is designed to deal with short indels. Despite the low k-mer recall, FAST-SG achieved a decent synthetic mate-pair read recall on this dataset from both long read technologies, and extracted near perfect synthetic mate-pair libraries. The synthetic mate-pair libraries can be used as input to a short read scaffolder to generate scaffold sequences through a combination of short and long read technologies.

Comparison of FAST-SG coupled with short read scaffolders against LINKS

We compared the results obtained by FAST-SG coupled with OPERA-LG [11], SCAFFMATCH [13] and BOSS [14] against LINKS [39], which is a scaffolder specifically designed to extract paired k-mers from long reads and employ them to join contigs.

FAST-SG and LINKS were applied with default parameters (k-mer of size 15) to create the synthetic mate-pair libraries in the range of 0.5kb to 8kb using as input the uncorrected long reads and Illumina assemblies available for both species (Table 1). Since LINKS performs better with high long read coverage [39], we subsampled 50X and 30X of coverage from *Escherichia coli* K12 and *Saccharomyces cerevisiae* W303, respectively.

FAST-SG is two times faster than LINKS and requires two orders of magnitude less memory to extract linking information from the long reads (Supplementary Table S9). The percentages of linked pairs extracted by both methods is comparable (with FAST-SG being slightly superior) and as expected, the percentage of linked pairs increases as a function of the insert size length for both long read technologies (Supplementary Table S10).

A more informative comparison involved assessing the quality of the scaffolds [3] produced by LINKS on one hand, and on the other, by the short read scaffolders coupled with FAST-SG. To evaluate the scaffolding results, the number of correct and erroneous joins were computed in each test case using the scripts provided in Hunt *et al.* [3]. Moreover, the F-Score metric (Short and long reads benchmarks subsection) was employed to summarise in a single statistics the performance of each scaffolder. Based on the F-Score values, the short read scaffolders using FAST-SG reached better or comparable results than LINKS (Figure 3). Moreover, LINKS produced more scaffolding errors in two out of the three datasets tested (Supplementary Table S11). With respect to the *E. coli* dataset, the scaffolding errors made by the short read scaffolders using FAST-SG (Figure 3) were related to the gap size estimation (type error 4), orientation (type errors 1 and 5), and relocation (type errors 8 and 12). The major source of errors in the scaffolds produced by LINKS was of type 5. This measures the correct orientation and distance between pairs of contigs (Figure 3). On the *S. cerevisiae* W303 dataset, the major source of scaffolding errors was translocation (type error 2) for both methods. However, LINKS has almost double the number of scaffolding errors compared to FAST-SG coupled with OPERA-LG or BOSS on this dataset (Figure 3, Supplementary Table S11).

Concerning the short-read scaffolders, BOSS and SCAFFMATCH reached higher F-score values than OPERA-LG (Figure 3). However, they tended to produce more scaffolding errors (Supplementary Table S11). It is important to notice also that the scaffolding errors observed here can be further reduced because fragmented Illumina assemblies (Table 1) were used in order to maximise the possibility of the scaffolders to make joins.

Overall, the performance of the short read scaffolders coupled with FAST-SG was superior or comparable to LINKS, a scaffolder specifically designed for long reads. FAST-SG thus allows the conversion of tools designed for short read scaffolding into a long

read scaffolder in a fast and modular way.

Using FAST-SG to perform the hybrid assembly of Arabidopsis thaliana (Ler-0)

An important goal of hybrid assembly methods is to reduce the long read coverage required to produce long range genome assemblies. Here, we examine the long read coverage required by our hybrid assembly method to produce long range hybrid assemblies comparable to CANU [4], which is a state-of-the-art *de novo* long read assembler.

Briefly, the hybrid assembly using FAST-SG proceeded as follows. In a first step, a single Illumina library (Table 1) covering 100X the *Arabidopsis thaliana* (Ler-0) genome was assembled using DISCOVARDENOVO [37], which is one of the best tools for assembling a single Illumina fragment (pair-end) library. The resulting assembly contained a total of 2,384 scaffolds with a N50 of 320kb and a total size of 119Mb (Table 2). The DISCOVARDENOVO assembly took 6.6 hours on 20 CPUs. In a second step, a total of 50X of PacBio reads (P5-C3) were error-corrected (Table 1), with the same Illumina reads used for the *de novo* assembly, using LORDEC. LORDEC took 14.2 hours on 20 CPUs. In a third step, the error-corrected long reads were randomly subsampled with a coverage between 5X to 50X, and FAST-SG (using 21-mers) was used to create 12 synthetic mate-pair libraries in the range of 1kb to 20kb for each subsample. The total number of mate-pair reads aligned at each coverage value ranged from 11.85 to 104.99 million for 5X to 50X, respectively (Supplementary Table 12). On average, 7.2% of the synthetic mate-pair reads aligned by FAST-SG were linking (*i.e.* connecting two different contigs) in each subsample. Moreover, a near perfect insert size distribution and a low percentage of outliers were observed for each synthetic library (Supplementary Figure S1). FAST-SG took 2.15 hours on 20 CPUs to process the whole dataset. Finally, OPERA-LG, BOSS and SCAFFMATCH were fed with the FAST-SG alignments to produce the scaffold sequences (Table 2). All short read scaffolders generated scaffold sequences in at most half an hour (OPERA-LG 22min, BOSS 24m and SCAFFMATCH 30min) using a single CPU.

The hybrid and the CANU assemblies available were structurally validated by a whole genome alignment against the reference *Arabidopsis thaliana* TAIR10 genome (Table 2, Supplementary Material 2).

As can be seen from Table 2, all hybrid assembly pipelines were able to produce long-range scaffolds (N50 > 1Mb) with a high coverage of the reference genome, low number of errors (<2.2%), low amount of sequence gaps (1.46Mb as maximum), and with an identity higher than any CANU assembly. All hybrid assemblies at 5X of coverage reached a N50 scaffold size comparable to the contig N50 obtained by a polished CANU assembly requiring 20X of coverage and 100X of Illumina reads (Table 2). Additionally, all hybrid assembly pipelines seemed to plateau after 30X of long read coverage as was previously observed on this dataset [4]. However, SCAFFMATCH, the most aggressive scaffolder tested, at 10X-30X of coverage produced accurate scaffolds having an N50 comparable to the CANU assemblies requiring 50X or 150X of coverage (Table 2).

All assemblies of *Arabidopsis thaliana* (Ler-0) were comparable in terms of the number and amount of sequences involved in structural errors (Table 2). Moreover, the major source of structural errors observed in both assembly strategies were mainly relocations, which explain more than 50% of the amount of sequences involved in miss-assemblies (Supplementary Figure S3).

Overall, we demonstrated that the hybrid assemblies were comparable in terms of continuity, completeness and accuracy to the assemblies obtained by CANU, which is considered a state-of-the-art *de novo* long read assembly pipeline. Furthermore, the proposed hybrid assembly strategy allowed faster and cheaper reconstructions of the *Arabidopsis thaliana* (Ler-0) genome and was remarkably efficient at shallow long read coverage (5X-10X).

Using FAST-SG to perform the hybrid assembly of a diploid human genome (NA12878)

An ultimate benchmark for any assembly method or sequencing technology is to assemble a complete human genome [40, 20, 22, 4, 34]. We performed a hybrid assembly of the Utah/Ceph NA12878 human diploid genome using a low coverage (5X) of ultra-long Nanopore reads (Table 1, Jain et al. [17]), a DISCOVARDENOVO assembly built from 50X of 250bp Illumina reads (Table 1, Weisenfeld

et al. [37]), FAST-SG and SCAFFMATCH [13].

FAST-SG (using 22-mers) was run to create 20 synthetic mate-pair libraries in the range of 2kb-180kb using as input a total of 1.4 million uncorrected Nanopore reads (N50 64.75Kb, Table 1), which have a total size of 23.11Gb and cover about 7X of the human genome. A total of 455.9 million synthetic mate-pair reads (11.15% linking contigs, Supplementary Table 13) were aligned to the DISCOVARDENOVO assembly, with a near perfect distribution of insert sizes and a low percentage of outliers observed (Supplementary Figure S2). FAST-SG required 8 hours using 20 CPUs to complete the task and used a maximum of 25Gb of memory. SCAFFMATCH was then fed with the alignments of FAST-SG and took 5.18 hours using a single CPU with a peak memory of 30.87Gb to generate the scaffold sequences. The resulting hybrid assembly is referred here to as the DFS (DISCOVARDENOVO+FAST-SG+SCAFFMATCH) assembly.

We evaluated the accuracy of the DFS assembly together with the public assemblies of NA12878 that were built using CANU [17], MASURCA [34], 10X genomics [22] and DOVETAIL genomics [20] by means of whole genome alignments against the complete human reference genome (Table 3).

In terms of continuity (N50, Table 3), the DFS assembly is more than 4X larger than a MASURCA hybrid assembly built with the same long read dataset and 100X of Illumina reads (http://masurca.blogspot.cl/2017/06/masurca-assembly-of-na12878-low.html). Moreover, it is comparable to a polished CANU assembly built with 35X of long read coverage [17]. DOVETAIL genomics and 10X genomics reached larger N50 scaffolds (Table 3), which are 2.5X and 3.7X larger than the DFS assembly, respectively. All assemblies are comparable in terms of size, 1-to-1 alignment length and coverage of the reference genome (Table 3).

In terms of identity (Table 3), DOVETAIL genomics and DFS are the leading pipelines. DOVETAIL genomics and DFS both use the DISCOVARDENOVO assembly as input for scaffolding. Both software maintain the high identity of the DISCOVARDENOVO assembly because contig bases are not changed in the scaffolding process.

As concerns the structural errors, all assembly pipelines are highly accurate with less than 1% of the total 1-to-1 alignment length involved in such errors (Table 3, Supplementary Figure S4). Moreover, translocation is the structural error that accumulates the greatest amount of miss-assembled bases on all assembly pipelines (Table 3). A more detailed inspection of the 1-to-1 alignments revealed that DFS, 10X genomics and DOVETAIL genomics tend to skip the short contigs (Supplementary Table S14), which is a known problem of scaffolding tools [3]. However, more complex miss-assemblies involving several structural errors were observed in the chimeric contigs assembled by CANU and MASURCA (Supplementary Table S15).

In terms of speed, the whole DFS pipeline (933 CPU hours) was 22X times faster than MASURCA (21.000 CPU hours; personal communication), 162X times faster than CANU (151.000 CPU hours Jain et al. [17]), and comparable to 10X genomics and DOVETAIL genomics.

Finally, we call attention to the fact that the hybrid assembly solution which we propose (using 14 flow cells and 50X of 250bp PE reads sequenced on Hiseq2500) is approximately 3 times cheaper than the CANU solution (using 53 flow cells and 50X of Illumina).

In summary, we demonstrated in this experiment that the DFS hybrid assembly pipeline produced an accurate and long-range reconstruction of a diploid human genome that was faster and cheaper than the current state-of-the-art long read assembly pipelines.

Compatibility of FAST-SG with Illumina mate-pair libraries

In this section, we explore the usefulness of FAST-SG as an alternative to commonly used short read alignment software for scaffolding graph construction from short read data. Indeed, Hunt *et al.* [3] demonstrated that the quality of the scaffolding results is highly dependent on the short read aligner used, and that precision is more important than maximizing the number of reads aligned to the contigs.

We assessed the performance of FAST-SG for aligning short reads on simulated Illumina data from the complete human reference

genome (GRCh38.p10, Supplementary Material 4) together with Bowtie [8], Bowtie2 [10], Bwa-Mem [36] and Bwa [9], which are commonly used short read aligners for constructing a scaffolding graph [3].

Our results show that the Fast-SG precision is high for any k-mer size (99.21% as minimum), is superior to Bowtie2-local (98.17%), and is comparable to Bowtie2-global (99.74%). However, Bwa-Mem (99.97%) is the leading tool (Supplementary Table S16). In terms of speed, Fast-SG performs the best. Indeed, it is between 7X to 14X times faster (depending on the k-mer size) than the next fastest program, which is Bowtie2-global (Supplementary Table S16). The recall of Fast-SG depends on the k-mer size used (Supplementary Table S16, Figure 1b). The recall of Fast-SG (71.67%) is comparable to the one bowtie (71.52%) for optimal k-mer values (k=25-30). Larger k-mer values ($k > 50$) decrease the recall of Fast-SG due to sequencing errors and read length. To map short reads of 101 base pairs in length, we therefore recommend to use k-mer values in the range of 25-30 base pairs.

A more informative evaluation consists in assessing the performance of Fast-SG on real Illumina data. Such evaluation was done on four real test cases (Table 1) and using four short read scaffolders. The short reads were aligned using Fast-SG and the aforementioned short read aligners. The scaffolders were fed with such alignments and run with identical commands overall (Supplementary Material 5).

In relation to the number of paired reads mapped (Supplementary Figure S5), Fast-SG aligned on average more pairs than Bowtie or Bwa, and was comparable to Bowtie2-global. It however aligns less pairs than Bowtie2-local or Bwa-Mem. From the number of paired reads aligned across the four test cases, we notice that the behaviour of Fast-SG depends on the k-mer size chosen. With larger sizes, Fast-SG resembles global methods, while with shorter sizes, it is closer to local methods (Supplementary Figure S5).

The average contig read-coverage statistics which is used to tag the repeated contigs before scaffolding [2] was extracted from the results of Opera-LG. Such statistics were employed to compute a pairwise Pearson correlation to determine the linear relationship between the short read aligners and Fast-SG (Supplementary Figure S6). We observe that the average contig read-coverage computed from the Fast-SG alignments correlated more on average with Bowtie ($\bar{x}$=0.933), Bwa ($\bar{x}$=0.905) and Bowtie2-global ($\bar{x}$=0.814) than with Bwa-Mem ($\bar{x}$=0.772) or Bowtie2-local ($\bar{x}$=0.725) on the datasets of *S. aureus*, *R. sphaeroides* and *P. falciparum* (Supplementary Figure S6).

The results of the four test cases in terms of F-score and error rate are illustrated in Figure 4 and detailed in Supplementary Tables S17 to S20. For almost all the test cases and scaffolding tools, Fast-SG reached the largest F-score (Figure 4) for some k-mer values. Moreover, Fast-SG had a superior average performance in terms of F-score in relation to the four scaffolders tested in 2 out of the 5 datasets (Figure 4, vertical lines) and allowed the scaffolding tools to obtain more accurate scaffolding results in 4 out of the 5 datasets (Figure 4, vertical lines).

The low GC content genome of *Plasmodium falciparum* proved to be particularly challenging to the scaffolders using local alignment methods (namely Bwa-Mem or Bowtie2-local). These indeed tended to produce several wrong joins (Figure 4), indicating that the local alignment methods are not an appropriate choice for scaffolding this genome. A possible explanation for the poor performance observed in this particular case is that the local alignment methods mapped 10% more reads than the global ones and than Fast-SG (Supplementary Figure S5), but there is a low correlation in the average contig read-coverage between the local alignment methods and Fast-SG (Supplementary Figure S6), suggesting many wrong mappings in the extra 10% aligned reads.

In conclusion, over the four test cases and four scaffolders benchmarked, Fast-SG consistently reached better scaffolding results than the short read aligners evaluated and may be considered as an effective tool for constructing a scaffolding graph from short reads.

Procedure for effective hybrid assembly with FAST-SG

The *de novo* assembly of a large genome is a difficult task. Genome complexity (size, repetitiveness, heterozygosity, polyploidy), as well as the algorithm and the sequencing platform adopted are all factors that may affect the quality of the resulting assembly. In this paper, we provide a procedure for an effective hybrid assembly using FAST-SG that is based on our experience and benchmark results.

The first step is to produce the best possible Illumina contig assembly (Figure 5, N50 > 100kb). To achieve this, we recommend the use of a single Illumina fragment library (paired-end) prepared using a PCR-free protocol (550 bp insert size). The Illumina library should be sequenced using either the MiSeq or the HiSeq2500 platform to generate paired reads of 250 bases at about 60X of genome coverage [37]. The Illumina library must be assembled with a de Bruijn graph assembler supporting a large k-mer size (k = 200) or a multi k-mer approach [41]. We tested DISCOVARDENOVO (k = 200) and obtained good quality contigs (N50 > 100Kb), but either ABYSS [42] or SPADES [41] can be used to create the contigs.

The second step is to sequence at shallow coverage (5X-10X) the longest possible reads by using the ONT or PacBio technologies (Figure 5). At the moment, we recommend the use of 1D ONT reads because the latest ONT machines produce longer reads than PacBio machines and FAST-SG is more adapted to the error profile of ONT than of PacBio. In cases where ONT reads are not available, we recommend to hybrid error correct the PacBio reads using LORDEC (k = 19 – 21) before applying FAST-SG (Figure 5).

The third step is to use FAST-SG to comprehensively extract linking information from the long reads by creating multiple-insert-size synthetic mate-pair libraries that lead to an improved scaffolding [43, 44]. In practice, we were able to create synthetic mate-pair libraries in the range sizes of 2-20 kb and 2-180 kb from PacBio and ONT (ultra-long reads), respectively. The k-mer size parameter of FAST-SG depends on the quality of the long reads. With raw long reads, we recommend to use short k-mer sizes (k = 17 – 22) to overcome the high error rate. Larger k-mer sizes (k = 17 – 40) can be used with error corrected long reads or with Illumina mate-pair libraries (Figure 5). After running FAST-SG, we recommend to verify the quality of each synthetic mate-pair library generated. To check the synthetic librairies, it is possible to plot the distribution of the observed insert size statistics which are computed from the read pairs aligned within contigs. Figure 2 and the Supplementary Figures S1 and S2 provide examples of such distribution. Additionally, statistics of the percentage of outliers and standard deviation can be computed from the observed insert sizes. For instance, a high percentage of outliers (>30%) or a larger than expected standard deviation (>30% of average) are both indicative of a low quality synthetic library. The latter must be discarded from the scaffolding step. FAST-SG computes and reports (log file) the observed average insert size for each synthetic library, which allows for an easy identification of low quality synthetic libraries.

The fourth step is to select a short read scaffolder. We showed that there are two classes of short read scaffolding tools, one more conservative (this class includes OPERA-LG and BESST2) and another more greedy (which includes BOSS and SCAFFMATCH). The greedier scaffolders reach higher F-score values than the conservative ones. However, the greedy ones tend to produce more scaffolding errors (Figure 3-4). According to our evaluations, we recommend a more greedy scaffolder (SCAFFMATCH) when the Illumina contig assembly is not highly fragmented (N50> 100Kb). Otherwise, a more conservative scaffolder (OPERA-LG) should be used to avoid scaffolding errors.

Finally, a full hybrid assembly example is described step-by-step in the following wiki-page of FAST-SG (https://github.com/adigenova/fast-sg/wiki/Hybrid-scaffolding-of-NA12878).

Discussion

The proposed hybrid assembly method could be improved by using the sequence between the synthetic mate pairs (inner sequence), either for assigning a new weight to the edges before scaffolding, or for placing the skipped contigs after scaffolding. An edge of the scaffolding graph can be re-weighted by computing the edit distance among the inner sequences and then eliminating the

pairs having a large edit distance. EDLIB [45] is an efficient library that could be used to perform this task. The skipped contigs can be unambiguously placed by computing a consensus sequence of the scaffolding gaps from the inner sequences, and then aligning the skipped contigs to the consensus gap sequence taking into account the lengths of the gap and of the skipped contig. The consensus of the inner sequences can be computed in a faster way using the SPOA library, which implements a partial order alignment algorithm [46]. These two improvements coupled with an appropriate ultra-long Nanopore read coverage (10X) could lead to an hybrid assembly pipeline that is superior to the current long-range mate-pair technologies where these improvements are not possible due to the fact that, in both technologies, the gap sequence between pairs is unknown.

Clearly, improvement in the base accuracy of long reads will increase the recall of FAST-SG and thus impact positively on the hybrid assembly process. Notice however that read recall is less important because not all the sequenced reads are useful for scaffolding, and indeed we showed with the Illumina scaffolding benchmarks that the short read aligners with higher read recall produced the worst scaffolding results. Additionally, FAST-SG was designed to enable constructing the scaffolding graph from uniquely mapped read pairs (FAST-SG index). It thus discards any repetitive sequence as they are not useful to build the scaffolding graph. Oxford Nanopore is a fast evolving technology and the current use of the new $1D^2$ chemistry or improvement in the base callers are two alternatives that could lead to an increased base accuracy of the ONT reads.

Conclusions

We introduced in this paper a new method, FAST-SG, that enables to construct a scaffolding graph from either short or long reads, allowing for an accurate construction of the scaffold sequences as well as for software reuse.

We showed that near perfect synthetic libraries are obtained with FAST-SG from either corrected or uncorrected PacBio and Nanopore long reads. The insert size is restricted to the actual long read size, but FAST-SG is able, using ultra-long Nanopore reads, to extract synthetic libraries of even Bacterial Artificial Chromosome clone sizes having insert size of 150kb-180kb. Those kinds of libraries were crucial to reach the high continuity of the current human reference genome [40]. An estimation of the gap size with the existing long-range mate-pair technologies (10X genomics and DOVETAIL genomics) is more challenging than with the synthetic libraries due to the fact that in such technologies, the linking information comes from a range of insert-sizes and the relative orientation of the read pairs may not be known (DOVETAIL genomics).

Clearly, the synthetic libraries eliminate the bottleneck of sequencing a combination of mate-pair libraries, which were typically required to obtain long-range assemblies [2, 23, 24]. We further showed that short read scaffolders are able to produce accurate scaffolds when they are fed with the synthetic libraries extracted by FAST-SG, thus leading to results that are superior to or match those obtained by LINKS, a scaffolder specifically designed for hybrid long read scaffolding. Futhermore, we showed that FAST-SG is faster than the current state-of-the-art short read aligners and that better results are achieved by the scaffolding tools when they are coupled with FAST-SG on illumina mate-pair data.

Finally, we demonstrated that FAST-SG in conjunction with efficient algorithms designed for Illumina data can be used to perform a full hybrid assembly of large genomes. The resulting assemblies are superior or comparable to the current state-of-the-art long read assembly pipelines. Additionally, the modular hybrid pipelines are faster and remarkably efficient at shallow long read coverage (5X-10X). The scalability to large genomes, moderate computational resources and the shallow long read coverage required by the proposed solution, represent significant improvements over the current hybrid assembly methods.

Overall, we believe that FAST-SG opens a door to achieve accurate hybrid long-range reconstructions of large genomes with low effort, high portability and low cost.

Availability of supporting data

Code snapshots and test data for demonstration of sequence assembly tools available in the GigaScience GigaDB repository [47].

Availability and requirements

- Project name: Fast-SG
- RRID (Research Resource Identification Initiative ID) : SCR_015934
- Project home page: https://github.com/adigenova/fast-sg
- Operating system(s): Unix, Linux and Mac OSX
- Programming language: C++ and PERL
- Other requirements: Compilation was tested with g++ version 5.3 (Linux) and clang version 4.2 (Mac OSX)
- License: MIT
- Any restrictions to use by non-academics: none

Additional files

Additional file 1 : The Supplementary-Material.pdf file contains the following Sections, Tables and Figures: **Sections**: Supplementary Material 1: *Software and datasets*. Supplementary Material 2: *Long read scaffolding benchmark*. Supplementary Material 3: *Arabidopsis thaliana (Ler-0) and human (NA12878) hybrid genome assemblies*. Supplementary Material 4: *Illumina alignment benchmark*. Supplementary Material 5: *Illumina scaffolding benchmark*. **Tables**: Supplementary Table S8 : *FAST-SG recall at k-mer and read level on synthetic mate-pair libraries extracted from corrected or uncorrected long reads using the E. coli K12 dataset*. Supplementary Table S9 : *Long read datasets used for comparison against LINKS*. Supplementary Table S10: *Number of k-mer pairs and read pairs extracted from raw long reads by LINKS and FAST-SG*. Supplementary Table S11 : *Long read scaffolding benchmark results for E. coli K12 and S. cerevisiae W303*. Supplementary Table 12 : *Number of synthetic read pairs aligned to the DISCOVARDENOVO assembly of Arabidopsis thaliana (Ler-0)*. Supplementary Table 13 : *Number of synthetic read pairs aligned to the human (NA12878) DISCOVARDENOVO assembly*. Supplementary Table S14 : *Example (blue rows) of short contig skipped in chromosome 6*. Supplementary Table S15 : *Example (blue rows) of chimeric contigs in chromosome 6 from the CANU and MASURCA assemblies*. Supplementary Table S16 : Short read alignment benchmark. **Figures**: Supplementary Figure S1: *Boxplot of synthetic libraries extracted by FAST-SG (K21) from the PacBio reads to scaffold the Arabidopsis thaliana (Ler-0) genome.* Supplementary Figure S2: *Boxplot of synthetic libraries extracted by FAST-SG (K22) from the ONT ultra-long reads to scaffold the human (NA12878) genome*. Supplementary Figure S3: *Amount of bases involved in structural errors by type in the Arabidopsis thaliana (Ler-0) assemblies*. Supplementary Figure S4: *NUCMER plots of the human (NA12878) assemblies*. Supplementary Figure S5: *Percentage of pair-end reads aligned by FAST-SG and the short read aligners for each Illumina dataset*. Supplementary Figure S6: *Pairwise contig read coverage correlation between the short read aligners and FAST-SG*. (PDF 2.5 Mb)

Additional file 2 : The Supplementary_Table_S17.xlsx file contains the Illumina scaffolding benchmark results for *S. aureus* using the SCAFFMATCH, OPERA-LG, BESST2 and BOSS scaffolders. (XLSX 56 Kb)

Additional file 3 : The Supplementary_Table_S18.xlsx file contains the Illumina scaffolding benchmark results for *R. sphaeroides* using the SCAFFMATCH, OPERA-LG, BESST2 and BOSS scaffolders. (XLSX 85 Kb)

Additional file 4 : The Supplementary_Table_S19.xlsx file contains the Illumina scaffolding benchmark results for *P. falciparum* using the SCAFFMATCH, OPERA-LG, BESST2 and BOSS scaffolders. (XLSX 132 Kb)

Additional file 5 : The Supplementary_Table_S20.xlsx file contains the Illumina scaffolding benchmark results for *H. sapiens*

using the ScaffMatch, Opera-LG, Besst2 and Boss scaffolders. (XLSX 101 Kb)

Declarations

List of abbreviations

ONT: Oxford Nanopore Technology PacBio: Pacific Biosciences

Competing Interests

The authors declare that they have no competing interests.

Funding

This work was supported by the Basal program PFB03 and CONICYT PFCHA/BECA DOCTORADO NACIONAL 2014/FOLIO 21140124 granted to ADG, as well as MAIA STIC-AmSud. This research was partially supported by the supercomputing infrastructure of the NLHPC (ECM-02) and ANR-16-CE23-0001 (ASTER) project.

Author's Contributions

MFS proposed the scaffolding problem. ADG devised the original ideas for Fast-SG. ADG developed, implemented and tested Fast-SG. MFS, GRA and AM provided helpful discussions and guidance for the project. ADG wrote the initial version of the manuscript. MFS improved the initial version of the manuscript. MFS, GRA and AM revised the manuscript. All authors read the paper and approved the final version.

References

1. Pop M. Genome assembly reborn: recent computational challenges. Briefings in Bioinformatics 2009 Jul;10(4):354–366.
2. Treangen TJ, Salzberg SL. Repetitive DNA and next-generation sequencing: computational challenges and solutions. Nature Reviews Genetics 2011 Nov;13(1):36–46.
3. Hunt M, Newbold C, Berriman M, Otto TD. A comprehensive evaluation of assembly scaffolding tools. Genome Biology 2014;15:R42.
4. Koren S, Walenz BP, Berlin K, Miller JR, Bergman NH, Phillippy AM. Canu: scalable and accurate long-read assembly via adaptive k-mer weighting and repeat separation. Genome Research 2017 May;27(5):722–736.
5. Kececioglu JD, Myers EW. Combinatorial algorithms for DNA sequence assembly. Algorithmica 1995 Feb;13(1-2):7.
6. Pevzner PA, Tang H, Waterman MS. An Eulerian path approach to DNA fragment assembly. Proceedings of the National Academy of Sciences 2001;98(17):9748–9753.
7. Huson DH, Reinert K, Myers EW. The Greedy Path-merging Algorithm for Contig Scaffolding. J ACM 2002 Sep;49(5):603–615.
8. Langmead B, Trapnell C, Pop M, Salzberg SL. Ultrafast and memory-efficient alignment of short DNA sequences to the human genome. Genome Biology 2009;10:R25.
9. Li H, Durbin R. Fast and accurate short read alignment with Burrows-Wheeler transform. Bioinformatics (Oxford, England) 2009 Jul;25(14):1754–1760.
10. Langmead B, Salzberg SL. Fast gapped-read alignment with Bowtie 2. Nature Methods 2012 Apr;9(4):357–359.

11. Gao S, Bertrand D, Chia BKH, Nagarajan N. OPERA-LG: efficient and exact scaffolding of large, repeat-rich eukaryotic genomes with performance guarantees. Genome Biology 2016 May;17:102.
12. Sahlin K, Chikhi R, Arvestad L. Assembly scaffolding with PE-contaminated mate-pair libraries. Bioinformatics (Oxford, England) 2016 Jul;32(13):1925–1932.
13. Mandric I, Zelikovsky A. ScaffMatch: scaffolding algorithm based on maximum weight matching. Bioinformatics (Oxford, England) 2015 Aug;31(16):2632–2638.
14. Luo J, Wang J, Zhang Z, Li M, Wu FX. BOSS: a novel scaffolding algorithm based on an optimized scaffold graph. Bioinformatics (Oxford, England) 2017 Jan;33(2):169–176.
15. Roberts RJ, Carneiro MO, Schatz MC. The advantages of SMRT sequencing. Genome Biology 2013 Jul;14(7):405.
16. Chin CS, Alexander DH, Marks P, Klammer AA, Drake J, Heiner C, et al. Nonhybrid, finished microbial genome assemblies from long-read SMRT sequencing data. Nature Methods 2013 Jun;10(6):563–569.
17. Jain M, Koren S, Quick J, Rand AC, Sasani TA, Tyson JR, et al. Nanopore sequencing and assembly of a human genome with ultra-long reads. Nature Biotechnology 2018;.
18. Jarvis DE, Ho YS, Lightfoot DJ, Schmöckel SM, Li B, Borm TJA, et al. The genome of Chenopodium quinoa. Nature 2017 Feb;542(7641):307–312.
19. Seo JS, Rhie A, Kim J, Lee S, Sohn MH, Kim CU, et al. De novo assembly and phasing of a Korean human genome. Nature 2016 Oct;538(7624):243–247.
20. Putnam NH, O'Connell BL, Stites JC, Rice BJ, Blanchette M, Calef R, et al. Chromosome-scale shotgun assembly using an in vitro method for long-range linkage. Genome Research 2016 Feb;.
21. Zheng GXY, Lau BT, Schnall-Levin M, Jarosz M, Bell JM, Hindson CM, et al. Haplotyping germline and cancer genomes with high-throughput linked-read sequencing. Nature Biotechnology 2016 Mar;34(3):303–311.
22. Weisenfeld NI, Kumar V, Shah P, Church DM, Jaffe DB. Direct determination of diploid genome sequences. Genome Research 2017 Apr;.
23. Williams LJS, Tabbaa DG, Li N, Berlin AM, Shea TP, MacCallum I, et al. Paired-end sequencing of Fosmid libraries by Illumina. Genome Research 2012 Nov;22(11):2241–2249.
24. Wu CC, Ye R, Jasinovica S, Wagner M, Godiska R, Tong AHY, et al. Long-span, mate-pair scaffolding and other methods for faster next-generation sequencing library creation. Nature Methods 2012 Sep;9(9).
25. Vinga S, Almeida J. Alignment-free sequence comparison—a review. Bioinformatics 2003 Mar;19(4):513–523.
26. Salzberg SL, Phillippy AM, Zimin A, Puiu D, Magoc T, Koren S, et al. GAGE: A critical evaluation of genome assemblies and assembly algorithms. Genome Research 2011 Dec;.
27. Limasset A, Rizk G, Chikhi R, Peterlongo P. Fast and scalable minimal perfect hashing for massive key sets. arXiv:170203154 [cs] 2017 Feb;ArXiv: 1702.03154.
28. Marchet C, Lecompte L, Limasset A, Bittner L, Peterlongo P. A resource-frugal probabilistic dictionary and applications in bioinformatics. arXiv:170300667 [cs, q-bio] 2017 Mar;ArXiv: 1703.00667.
29. Kokot M, Długosz M, Deorowicz S. KMC 3: counting and manipulating k-mer statistics. Bioinformatics 2017 May;.
30. Karp RM, Rabin MO. Efficient randomized pattern-matching algorithms. IBM Journal of Research and Development 1987 Mar;31(2):249–260.
31. Mohamadi H, Chu J, Vandervalk BP, Birol I. ntHash: recursive nucleotide hashing. Bioinformatics 2016 Nov;32(22):3492–3494.
32. Ning Z, Cox AJ, Mullikin JC. SSAHA: A Fast Search Method for Large DNA Databases. Genome Research 2001 Oct;11(10):1725–1729.
33. Salmela L, Rivals E. LoRDEC: accurate and efficient long read error correction. Bioinformatics 2014 Dec;30(24):3506–3514.

34. Zimin AV, Puiu D, Luo MC, Zhu T, Koren S, Marcais G, et al. Hybrid assembly of the large and highly repetitive genome of Aegilops tauschii, a progenitor of bread wheat, with the mega-reads algorithm. Genome Research 2017 Jan;p. gr.213405.116.
35. Li H, Handsaker B, Wysoker A, Fennell T, Ruan J, Homer N, et al. The Sequence Alignment/Map format and SAMtools. Bioinformatics (Oxford, England) 2009 Aug;25(16):2078–2079.
36. Li H. Aligning sequence reads, clone sequences and assembly contigs with BWA-MEM. arXiv:13033997 [q-bio] 2013 Mar;ArXiv: 1303.3997.
37. Weisenfeld NI, Yin S, Sharpe T, Lau B, Hegarty R, Holmes L, et al. Comprehensive variation discovery in single human genomes. Nature Genetics 2014 Dec;46(12):1350–1355.
38. Kurtz S, Phillippy A, Delcher AL, Smoot M, Shumway M, Antonescu C, et al. Versatile and open software for comparing large genomes. Genome Biology 2004;5(2):R12.
39. Warren RL, Yang C, Vandervalk BP, Behsaz B, Lagman A, Jones SJM, et al. LINKS: Scalable, alignment-free scaffolding of draft genomes with long reads. GigaScience 2015;4:35.
40. Schneider VA, Graves-Lindsay T, Howe K, Bouk N, Chen HC, Kitts PA, et al. Evaluation of GRCh38 and de novo haploid genome assemblies demonstrates the enduring quality of the reference assembly. Genome Research 2017 May;27(5):849–864.
41. Bankevich A, Nurk S, Antipov D, Gurevich AA, Dvorkin M, Kulikov AS, et al. SPAdes: a new genome assembly algorithm and its applications to single-cell sequencing. Journal of computational biology 2012;19(5):455–477.
42. Jackman SD, Vandervalk BP, Mohamadi H, Chu J, Yeo S, Hammond SA, et al. ABySS 2.0: resource-efficient assembly of large genomes using a Bloom filter. Genome research 2017;27(5):768–777.
43. Myers EW, Sutton GG, Delcher AL, Dew IM, Fasulo DP, Flanigan MJ, et al. A whole-genome assembly of Drosophila. Science 2000;287(5461):2196–2204.
44. Gnerre S, MacCallum I, Przybylski D, Ribeiro FJ, Burton JN, Walker BJ, et al. High-quality draft assemblies of mammalian genomes from massively parallel sequence data. Proceedings of the National Academy of Sciences 2011;108(4):1513–1518.
45. Šošić M, Šikić M. Edlib: a C/C++ library for fast, exact sequence alignment using edit distance. Bioinformatics (Oxford, England) 2017 Jan;.
46. Lee C. Generating consensus sequences from partial order multiple sequence alignment graphs. Bioinformatics 2003 May;19(8):999–1008.
47. Di Genova A, Ruz GA, Sagot MF, Maass A. Software and supporting data for "Fast-SG: An alignment-free algorithm for hybrid assembly". GigaScience Database;http://dx.doi.org/10.5524/100437.
48. Walker BJ, Abeel T, Shea T, Priest M, Abouelliel A, Sakthikumar S, et al. Pilon: An Integrated Tool for Comprehensive Microbial Variant Detection and Genome Assembly Improvement. PLoS ONE 2014 Nov;9(11).

Figure 1: A) Overview of the FAST-SG algorithm. B) Number of unique k-mers (y-axis) in the human genome GRCh38.p10 as a function of the k-mer size (x-axis). C) Memory required for indexing the unique k-mers of the human genome by FAST-SG and using an exact implementation. The blue dotted-line shows the memory required by FAST-SG as a function of the k-mer size. In green is shown the memory required by an exact implementation which uses two bits per base. The amount of memory used by such implementation increases as a function of the k-mer size (x-axis). The memory of the index used in FAST-SG only increases with the number of k-mers to store.

Figure 2: Boxplots of the insert size distribution observed for each synthetic library in the genome of *Escherichia coli* K12. The boxplots were drawn extracting from the FAST-SG alignments a minimum of 5,000 insert sizes from the mate-pair reads mapped within contigs for each combination of synthetic library and long read technology. The percentage of outliers detected in the raw ONT reads ranged from a minimum of 0.37% (0.5kb) to a maximum of 4.24% (8kb), while for raw PacBio it ranged from a minimum of 0.25% (0.5kb) to a maximum of 9.85% (8kb). The number of outliers increased with the error-correction for both long read technologies, reaching an average of 9.32% (std 1.73%) and 8.32% (std 3.74%) for the ONT and PacBio reads, respectively. The boxplots were drawn excluding outliers.

Figure 3 : Synthetic libraries scaffolding benchmark. The F-Score (Methods Section) was computed with the scripts provided by Hunt *et al.* [3] on the scaffold sequences produced by each scaffolding tool. The Pie-chart display the number of scaffolding error for LINKS and for the short read scaffolders fed with the FAST-SG alignments for both *E. coli* K12 and *S. cerevisiae* W303. The definition of the scaffolding errors (colours in Pie-chart) are provided in the short and long reads benchmarks subsection

Figure 4: Illumina scaffolding benchmark. Four real datasets (Table 1), five Illumina libraries and four scaffolding tools were used to assess the performance of FAST-SG and the short read aligners for building the scaffolding graph by means of an F-score metric and percentage of wrong joins (Algorithms Section, and Supplementary Material 4). FAST-SG was run with various k-mer sizes in the range of k=12-28, k=12-70, k=15-66 and k=15-80 for *Staphylococcus aureus*, *Rhodobacter sphaeroides*, *Plasmodium falciparum* and the human chromosome 14, respectively. Short read aligners were run with the wrapper or instructions provided by the scaffolding tools when possible, or using the default parameters. Single data points provide the F-Score and error rate for each combination of scaffolding tool and aligner in each dataset. The vertical lines show for each dataset the average F-score or Error rate values obtained by each of the short read aligners or FAST-SG together with the four scaffolding tools. Vertical lines for BOWTIE were not plotted since it cannot be used with BESST2. For the *Plasmodium falciparum* (short) dataset, the average F-Score (vertical lines) were omitted for BWA, BWA-MEM and BOWTIE2-LOCAL due to a poor performance (High error rate). The commands used for the aligners and scaffolding tools are detailed in the Supplementary Material 5.

Figure 5: FAST-SG hybrid assembly workflow. Thick black lines represent the common path for hybrid assembly with FAST-SG. Thin black lines show alternative paths when long reads were error corrected or Illumina mate-pair libraries were sequenced.

Table 1. Sequencing datasets and Illumina assemblies used to evaluate the performance of Fast-SG

**Long read datasets**						
	**#Reads**	**Average read length (bp)**	**Technology**	**Machine**	**Illumina assemblies**	
					#Contigs	N50
E.coli K12	164,472	9,009	ONT	R9.2	140	106,241
	1,192,955	4,412	PacBIO	Sequel System		
	22,391,084	298	Illumina	MiSeq		
S.cerevisiae W303	594,243	4,795	PacBio	PacBio	890	52,324
A. thaliana (Ler-0)	561,176	9,633	PacBio	Sequel System	2,384	320,571
	46,129,480	300	Illumina	MiSeq		
Human (NA12878)	1,415,868	16,324	ONT	R9.4	37,393	202,174
**Short read datasets**						
	**#Reads**	**Read length**	**Insert size**	**SRA/ENA**	**Illumina assemblies**	
					#Contigs	N50
S.aureus	3,494,070	37	3,500	SRR022865	170	47,016
R.sphaeroides	2,050,868	101	3,500	SRR034528	577	15,351
P.falciparum (short)	52,542,302	76	550	ERR034295	9,318	2,995
P.falciparum (long)	1,562,080	75	3,000	ERR163027		
H.sapiens chr14	22,669,408	101	2,600	SRR067771	19,936	12,963

Further details are provided in the Data description subsection and in the Supplementary Material 1.

Table 2. Hybrid and long read assemblies of *Arabidopsis thaliana* (Ler-0)

**Number Scaffolds**	**Max**	**N50**	**Size (Mb)**	**Fold**	**LRC**	**Scaffolder / Assembler**	**BreakPoints**			**1-to-1 identity**	**% Ref covered**
							Number	Bases (Mb)	% Error		
2,384	1,551,485	320,571	119.45	1.00	-	Discovar	91	0.48	0.49	99.07	82.044
1,577	5,305,497	1,076,408	120.05	3.36	5X	Opera-LG	174	0.978	1.00	99.07	82.054
1,368	9,953,317	2,475,756	120.26	7.72	10X	Opera-LG	202	1.197	1.22	99.07	82.047
1,249	16,906,870	4,165,132	120.32	12.99	15X	Opera-LG	206	1.237	1.26	99.07	82.052
1,179	18,032,662	4,941,257	120.41	15.41	20X	Opera-LG	218	1.588	1.62	99.07	82.060
1,103	14,710,653	4,756,724	120.43	14.84	30X	Opera-LG	227	1.728	1.76	99.07	82.055
1,049	10,003,725	4,667,601	120.41	14.56	50X	Opera-LG	230	1.732	1.76	99.07	82.060
1,345	8,867,374	1,632,787	120.40	5.09	5X	ScaffM	195	1.620	1.65	99.07	82.058
1,143	8,867,059	5,142,417	120.65	16.04	10X	ScaffM	203	1.319	1.34	99.07	82.045
1,072	11,814,750	6,165,459	120.73	19.23	15X	ScaffM	205	1.330	1.36	99.07	82.045
1,020	11,873,221	6,221,109	120.80	19.41	20X	ScaffM	207	1.477	1.50	99.07	82.039
958	13,946,812	7,073,179	120.90	22.06	30X	ScaffM	209	1.651	1.68	99.07	82.042
923	13,957,620	6,292,557	120.85	19.63	50X	ScaffM	210	1.712	1.74	99.07	82.041
1,593	5,296,335	1,037,785	119.96	3.24	5X	Boss	179	1.171	1.19	99.07	82.061
1,371	13,608,688	2,554,739	120.17	7.97	10X	Boss	200	1.335	1.36	99.07	82.054
1,239	13,643,115	2,829,628	120.22	8.83	15X	Boss	207	1.189	1.21	99.07	82.061
1,173	7,977,908	3,005,451	120.23	9.38	20X	Boss	212	1.564	1.59	99.07	82.060
1,093	9,004,636	2,974,378	120.28	9.28	30X	Boss	219	1.575	1.60	99.07	82.057
1,031	11,011,921	3,179,270	120.29	9.92	50X	Boss	229	2.162	2.20	99.07	82.050
1,439	447,211	80,063	89.84	-	10X	Canu	107	0.675	1.10	98.19	51.188
259	4,542,617	1,170,676	118.25	-	20X	Canu-p	201	0.969	0.99	99.06	81.907
258	4,543,625	1,170,942	118.31	-	20X	Canu-q	183	0.831	0.85	99.02	81.808
259	4,535,400	1,168,180	118.05	-	20X	Canu	185	1.030	1.09	98.82	78.874
119	15,152,700	6,219,401	120.67	-	50X	Canu	219	1.766	1.79	99.02	82.565
88	15,945,651	8,307,845	121.45	-	150X	Canu	215	1.935	1.95	99.06	82.938

Continuity was measured using maximum and N50 scaffold/contig size, where N50 is the longest scaffold/contig such that scaffolds/contigs of this length or greater sum at least half of the assembly size. The quality of the assembly was evaluated via a direct comparison against the *Arabidopsis thaliana* TAIR10 reference genome using Nucmer [38] and reported using the Gage [26] statistics, which from 1-to-1 alignments evaluates both identity and structural breakpoints (inversions, relocations and translocations). An optimal assembly has high continuity, low breakpoint errors, high identity and high coverage of the reference genome. LCR stands for Long Read Coverage. Canu-p and Canu-q are Canu assemblies polished with Pilon [48] and Quiver respectively. Pilon and Quiver are tools used after a long read assembly to improve the quality of the consensus sequence. All datasets and commands used for the hybrid assembly of *Arabidopsis thaliana* (Ler-0) are detailed in Table 1 and the Supplementary Materials 2 and 3.

Table 3. Hybrid and long read assemblies of NA12878

		DISCOVAR	DFS	10X	DOVETAIL	CANU-P	MASURCA
**Assembly statistics**	Number	37,393	7,323	9,926	9,463	2,337	4,885
	Min	2,000	2,000	2,000	2,000	2,981	4,103
	Max	1,380,479	30,548,185	69,726,354	95,295,052	50,410,306	9,066,374
	N50	202,174	6,445,123	16,305,019	24,472,662	7,667,013	1,695,766
	Size	2,794,627,041	2,884,349,664	2,835,096,130	2,800,321,128	2,866,880,913	2,849,443,591
**Long read coverage**		-	7X	-	-	35X	7X
**1-to-1 alignments**							
	Length	2,793,980,166	2,797,898,328	2,778,947,064	2,799,630,879	2,811,439,829	2,845,550,340
	Identity	99.8	99.8	99.79	99.8	99.28	99.67
	% Ref covered	90.16	90.29	89.68	90.35	90.73	91.83
**Breakpoints**							
Relocations	Number	120	1151	688	997	501	374
	Bases (Mb)	0.361	5.604	4.810	0.582	2.281	2.071
Translocations	Number	373	1,856	883	976	1,082	941
	Bases (Mb)	4.840	11.279	7.838	6.576	13.781	13.933
Inversions	Number	53	768	871	2,813	299	240
	Bases (Mb)	0.151	3.886	7.273	0.736	2.903	3.008
Total	Number	546	3,775	2,442	4,786	1,882	1,555
	Bases (Mb)	5.353	20.769	19.921	7.894	18.964	19.012
	%1-to-1	0.192	0.742	0.717	0.282	0.675	0.668

Assembly statistics: *Number* - number of contigs/scaffolds assembled, *Max/Min* - the maximum / minimum contig/scaffold size in base pairs; *N50* - contig/scaffold length such that half of the assembly size is obtained by adding contigs/scaffolds sorted in descending order by length; *size* - total size of the assembly in base pairs; 1-to-1 alignments: *length* - total length of non-repetitive alignments between the assembly and GRCh38.p10 detected by NUCMER; *identity* - average identity between the assembly and GRCh38.p10 computed from the 1-to-1 alignments; *%Ref covered*, percentage of the GRCh38.p10 that is covered by 1-to-1 alignments where the length of the reference was set to 3.1Gb. Breakpoints: structural errors were obtained from 1-to-1 alignments and reported using the GAGE metrics (Relocations, Translocations and Inversions); *number* - counts the number of breakpoints by sort; *bases (Mb)* - adds the number of bases involved in breakpoints extracted from the DNADIFF report (qdiff file) in mega bases; *%1-to-1* - percentage of structural errors with respect to the total 1-to-1 alignment length. Public NA12878 assemblies were downloaded and used for validation and comparisons against the DFS hybrid assembly pipeline.

Figure 1

Click here to download Figure Figure1.pdf

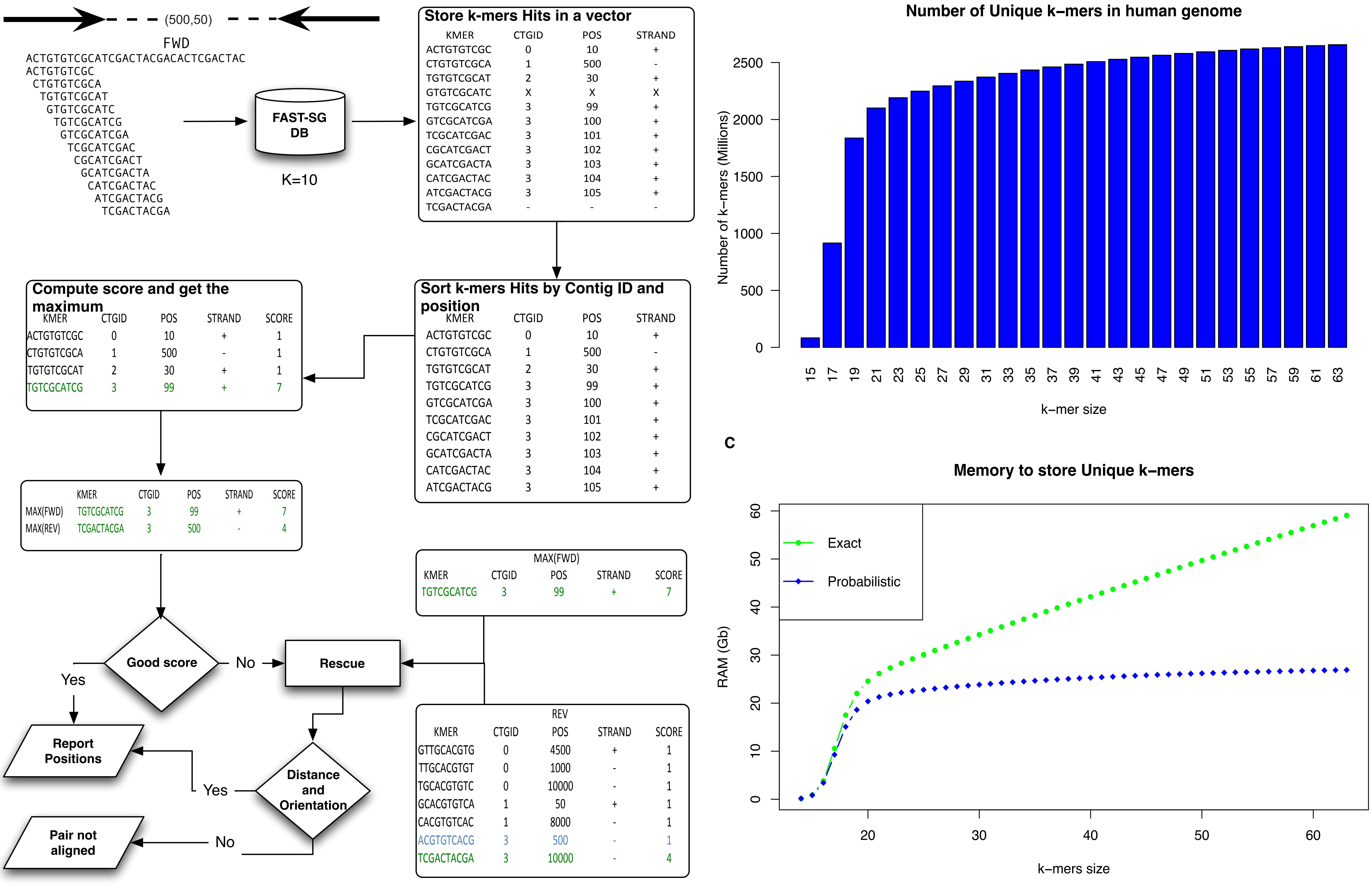

Figure 2

Click here to download Figure Figure2.pdf

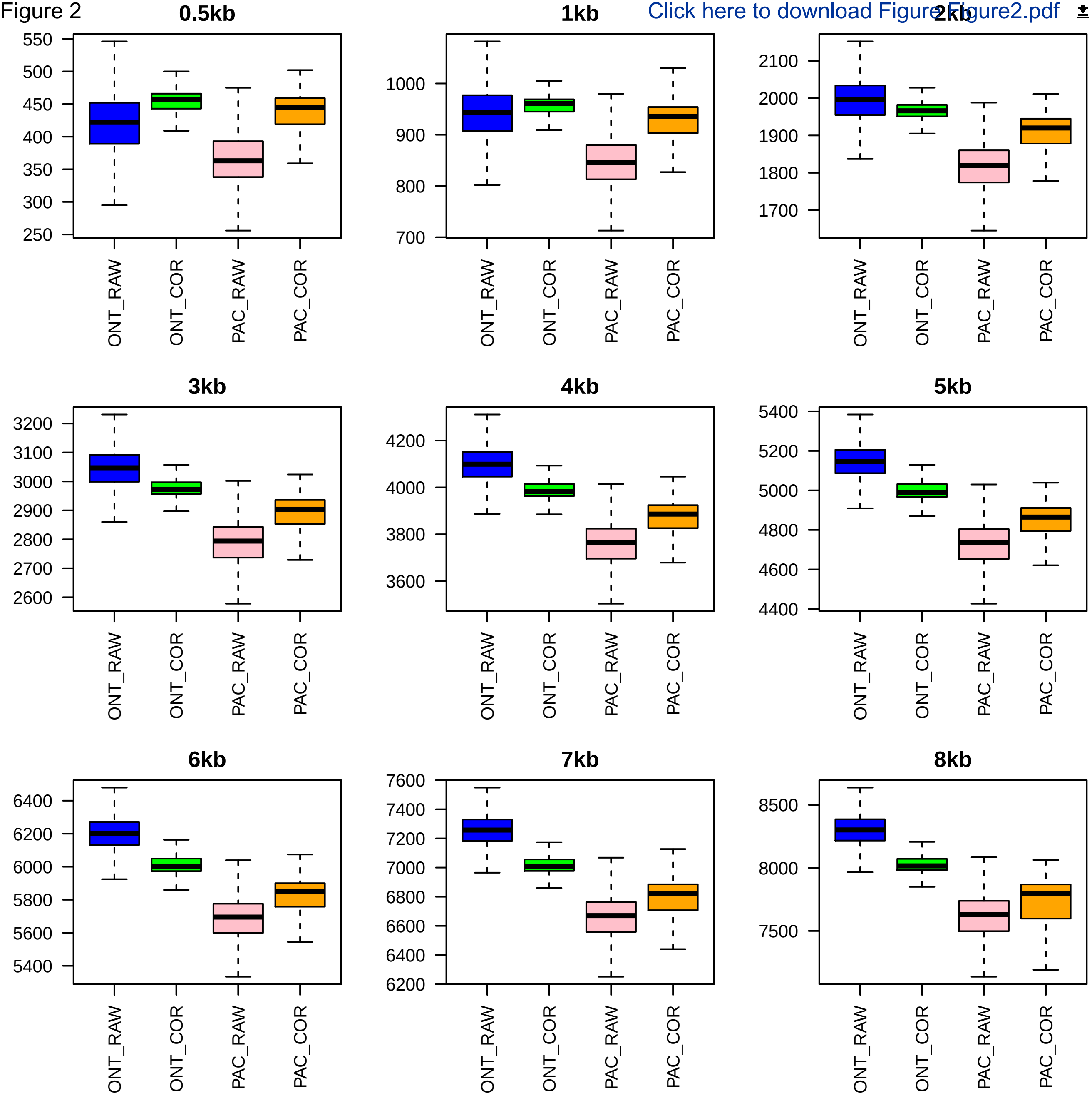

Figure 4

Click here to download Figure Figure

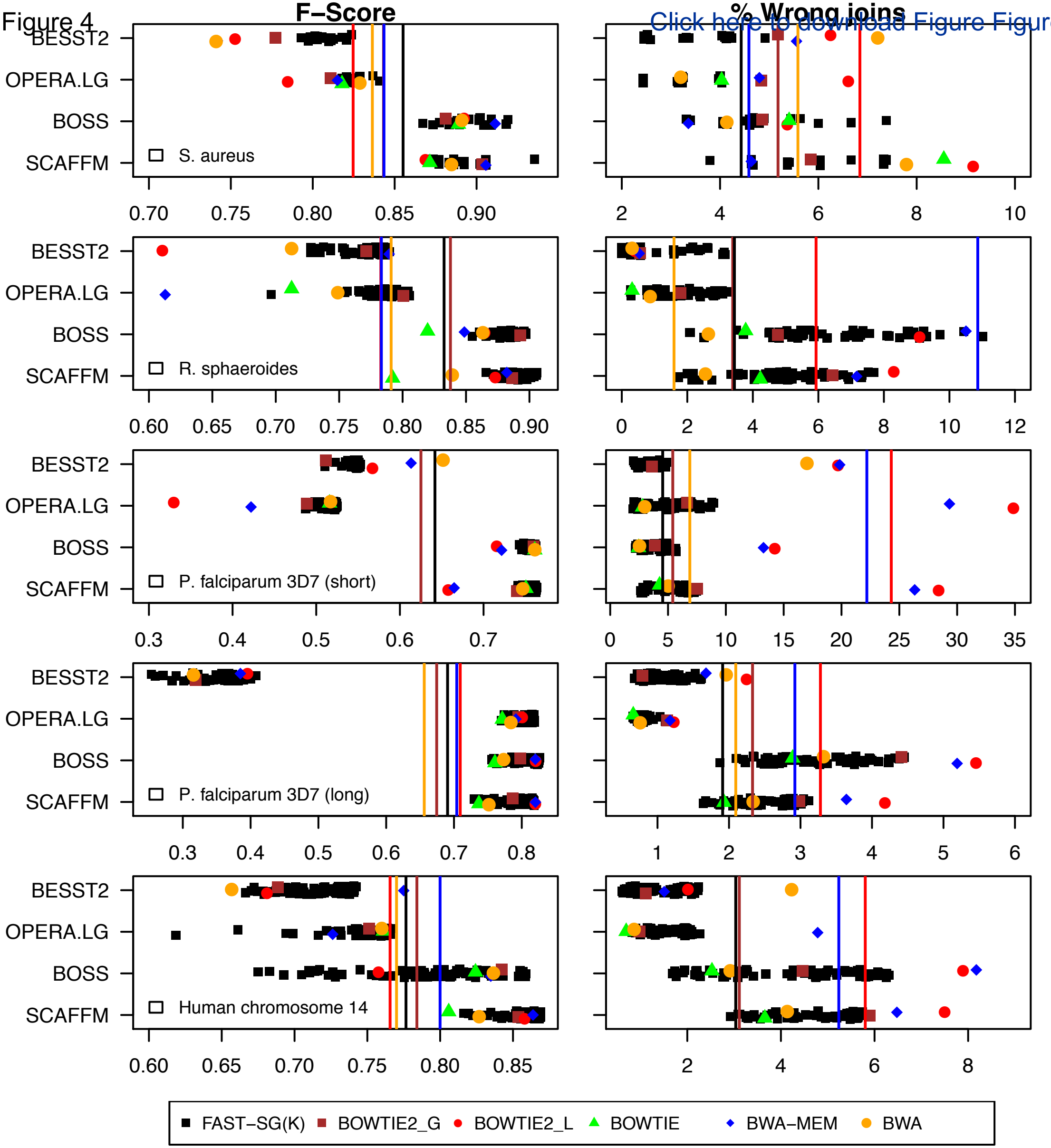

Figure 5

Click here to download Figure Figure5.pdf

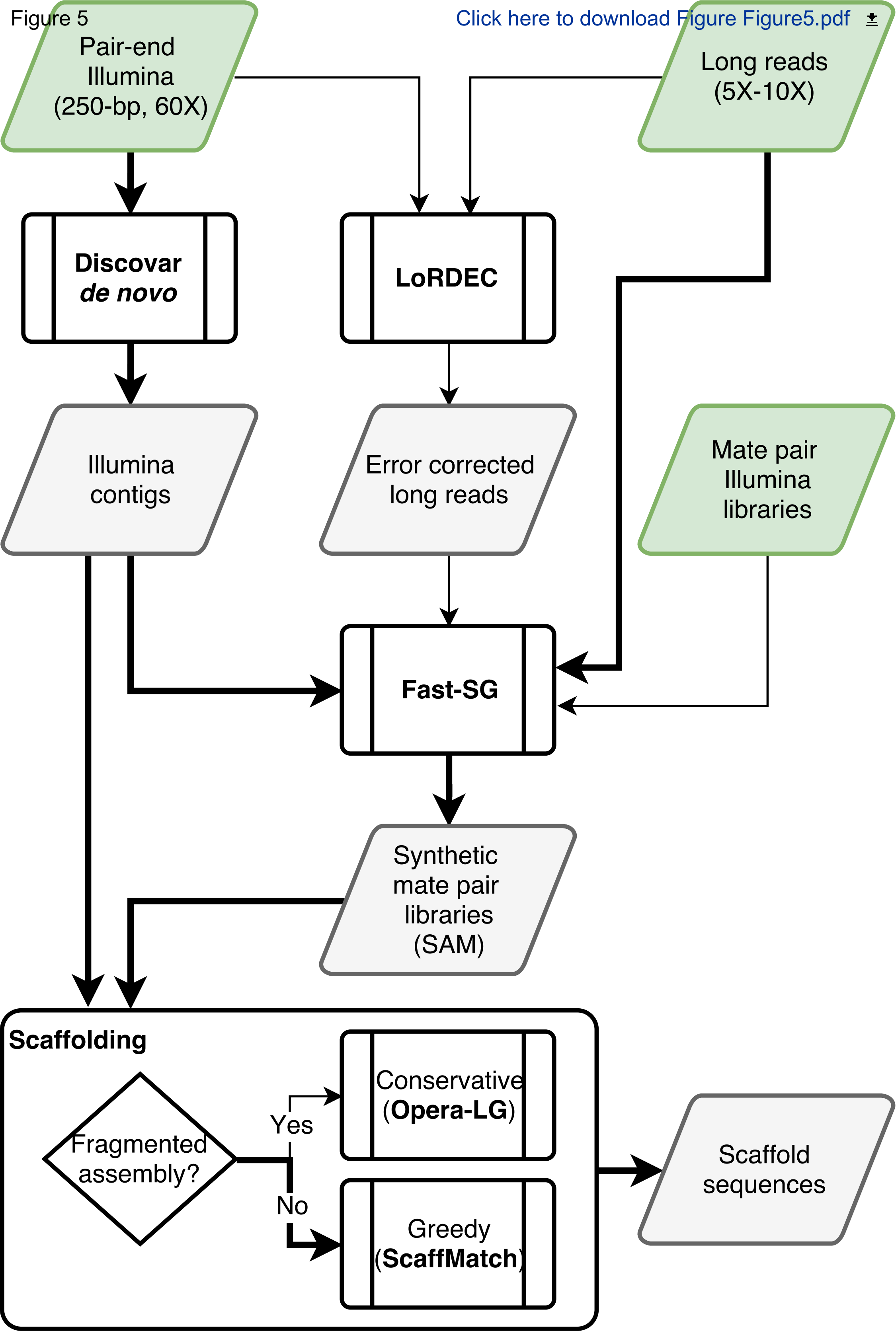

Figure 3

Click here to download Figure Figure3.pdf

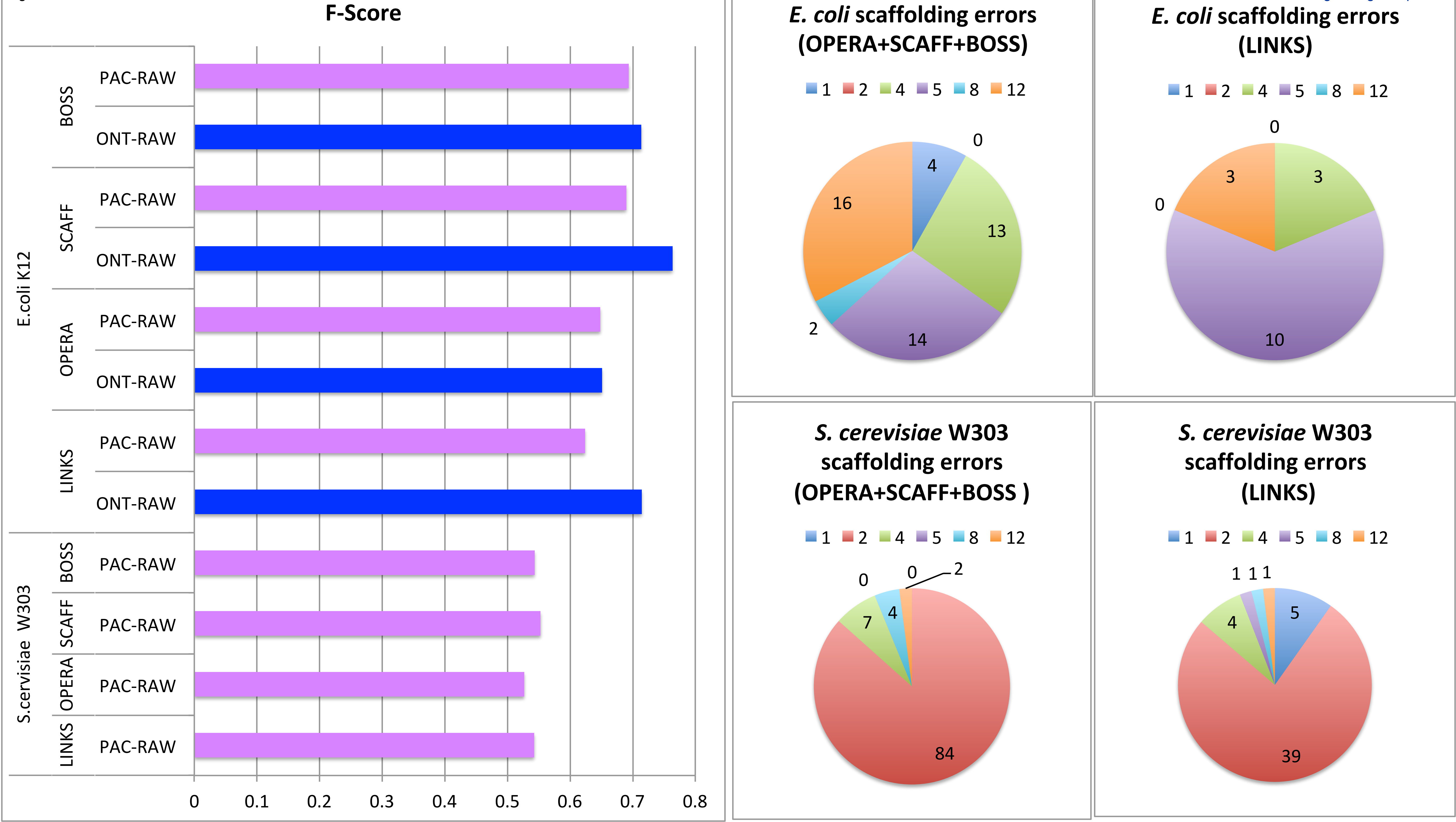

Click here to access/download
Supplementary Material
Supplementary-Material.pdf

Supplementary Table S17

Click here to access/download
Supplementary Material
Supplementary_Table_S17.xlsx

Supplementary Table S18

Click here to access/download
Supplementary Material
Supplementary_Table_S18.xlsx

Supplementary Table S19

Click here to access/download
Supplementary Material
Supplementary_Table_S19.xlsx

Supplementary Table S20

Click here to access/download
Supplementary Material
Supplementary_Table_S20.xlsx
