# Supplementary material for: Fast-SG: an alignment-free algorithm for hybrid assembly
Source: Gigascience. 2018 May 5;7(5):giy048. doi: 10.1093/gigascience/giy048 (PMC6007556; doi:10.1093/gigascience/giy048)
Supplement: Supplement materials [file giy048_supp.zip › Supplementary-Material.pdf]

Supplementary Material for "FAST-SG: An alignment-free algorithm for hybrid assembly."

Index of Contents

Index of Contents ---------- 1
Index of Supplementary Tables ---------- 3
Index of Supplementary Figures ---------- 4
Supplementary Material 1: Software and datasets. ---------- 5
Software ---------- 5
Short read aligners ---------- 5
Short read scaffolders ---------- 5
Long read software ---------- 5
Benchmark software ---------- 5
Other software ---------- 5
Datasets ---------- 6
Short read datasets ---------- 6
Long read datasets ---------- 6
Nanopore reads and human (NA12878) genome assemblies ---------- 6
Reference genomes ---------- 6
Supplementary Material 2: Long read scaffolding benchmark. ---------- 10
Long read error correction ---------- 10
Long read scaffolding ---------- 10
E.coli K12 dataset ---------- 10
S. cerevisiae W303 dataset ---------- 12
A. thaliana (Ler-0) dataset ---------- 12
Long read scaffolding of human (NA12878) ---------- 13
Long read scaffolding validation ---------- 14
E. coli K12 dataset ---------- 14
S. cerevisiae W303 dataset ---------- 14
A. thaliana (Ler-0) dataset ---------- 14
Human (NA12878) dataset ---------- 15
Synthetic libraries and comparison of FAST-SG against LINKS ---------- 15
Supplementary Material 3: *Arabidopsis thaliana* (Ler-0) and human (NA12878) hybrid genome assemblies. ---------- 18
FAST-SG alignments ---------- 18
Synthetic libraries ---------- 18
Structural errors ---------- 18
Supplementary Material 4: Illumina alignment benchmark. ---------- 26
Simulated Illumina reads ---------- 26
Short read alignments ---------- 26
Supplemental Material 5: Illumina scaffolding benchmark. ---------- 29
Short read alignments by scaffolder ---------- 29
OPERA-LG/BESST2 alignments ---------- 29
SCAFFMATCH/BOSS alignments ---------- 29
Scaffolder settings by dataset ---------- 30
OPERA-LG settings ---------- 30
SCAFFMATCH settings ---------- 30

BESST2 settings ---------- 31
BOSS settings ---------- 31
Validation of the scaffolding results ---------- 32
Scaffolding benchmark results for *S. aureus* ---------- 35
Scaffolding benchmark results for *R. sphaeroides* ---------- 35
Scaffolding benchmark results for *P. falciparum* ---------- 35
Scaffolding benchmark results for *H. sapiens* ---------- 35
References ---------- 36

Index of Supplementary Tables

Table S1: Description of the software used within the manuscript. 7
Table S2: Datasets used to perform the short read scaffolding benchmarks. 7
Table S3: Datasets used to perform the long read scaffolding benchmarks. 8
Table S4: Illumina assemblies used in the scaffolding benchmarks. 8
Table S5: CANU long read assemblies of *Arabidopsis thaliana* (Ler-0) genome at different coverage. 8
Table S6: Oxford Nanopore dataset used for the hybrid assembly of the human genome (NA12878). 9
Table S7: Public human (NA12878) assemblies used for validation and hybrid assembly. 9
Table S8: FAST-SG recall at *k*-mer and read level on synthetic mate-pair libraries extracted from corrected or uncorrected long reads using the *E. coli* K12 dataset. 15
Table S9: Long read datasets used for comparison against LINKS. 15
Table S10: Number of *k*-mer pairs and read pairs extracted from raw long reads by LINKS and FAST-SG. The percentage of linking *k*-mer pairs and linking read-pairs is detailed for each synthetic library. 16
Table S11: Long read scaffolding benchmark results for *E. coli* K12 and *S. cerevisiae* W303. FAST-SG coupled with short read scaffolders was compared against LINKS. 17
Table S12: Number of synthetic read pairs aligned to the DISCOVAR$_{DENOVO}$ assembly of *Arabidopsis thaliana* (Ler-0) at various subsamples of long read coverage and percentage of linking pairs by synthetic library. 19
Table S13: Number of synthetic read pairs aligned to the human (NA12878) DISCOVAR$_{DENOVO}$ assembly and percentage of linking pairs by library. 19
Table S14: Example (blue rows) of short contigs skipped in chromosome 6 by the 10X genomics, DOVETAIL genomics and DISCOVAR+FAST-SG+SCAFFMATCH assembly pipelines. 24
Table S15: Examples (blue rows) of chimeric contigs in chromosome 6 from the CANU and MASURCA asssemblies. 25
Table S16: Short read alignment benchmark. 28

Index of Supplementary Figures

Figure S1: Boxplot of synthetic libraries extracted by FAST-SG (K21) from the PacBio reads to scaffold the *Arabidopsis thaliana* (Ler-0) genome. 20
Figure S2: Boxplot of the synthetic libraries extracted by FAST-SG (K22) from the ONT ultra-long reads to scaffold the human (NA12878) genome. 21
Figure S3: Amount of bases involved in structural errors by type in the *Arabidopsis thaliana* (Ler-0) assemblies. 22
Figure S4: Nucmer plots of the human (NA12878) assemblies. In parenthesis, we show the number of structural errors and the amount of miss-assembled sequences for each assembly. 23
Figure S5: Percentage of pair-end reads aligned by FAST-SG (K) and the short read aligners for each Illumina dataset. The horizontal line is the average percentage of mate-pairs aligned by FAST-SG considering all *k*-mer sizes. 33
Figure S6: Pairwise contig read coverage correlation between the short read aligners and FAST-SG. 34

Supplementary Material 1: Software and datasets.

Software

Short read aligners

We compared FAST-SG against the commonly used short read aligners for constructing the scaffolding graph from Illumina reads. In particular, we compared FAST-SG against BOWTIE (Langmead *et al.* 2009), BOWTIE2 (Langmead and Salzberg 2012), BWA (Li and Durbin 2009) and BWA-MEM (Li 2013). The versions and URLs for each short-read aligner are provided in Table S1.

Short read scaffolders

Four short read scaffolders were used to benchmark FAST-SG against the short read aligners when building the scaffold sequences. We used scaffolders that accept an input in the SAM/BAM file format (Li *et al.* 2009). In particular, BOSS (Luo *et al.* 2017) and SCAFFMATCH (Mandric and Zelikovsky 2015) accept an input in SAM/BAM single-end format and OPERA-LG (Gao *et al.* 2016) in SAM/BAM single or paired, however BESST2 (Sahlin *et al.* 2016) only accepts sorted indexes BAM in pair-end mode. FAST-SG writes the result in the SAM format in single or pair-end mode. SAMTOOLS (Li *et al.* 2009) was used for handling the SAM/BAM files when required. The versions and URLs for each short-read scaffolder are provided in Table S1.

Long read software

FAST-SG coupled with the short read scaffolders was compared against LINKS (Warren *et al.* 2015) on two long-read datasets. Additionally, we used LORDEC (Salmela and Rivals 2014) to hybrid error-correct the long reads in order to assess the performance of FAST-SG to extract synthetic mate-pair libraries from raw or error-corrected long reads. The versions and URLs for LINKS and LORDEC are provided in Table S1.

Benchmark software

We used the benchmark described in Hunt *et al.* (2014), to assess the quality of the scaffold sequences produced by short read scaffolders using input from short read aligners and FAST-SG. Additionally, we created two long read benchmarks using the tools of Hunt *et al.* (2014) to assess the quality of the hybrid scaffoldings (LINKS against FAST-SG coupled with the short read scaffolders). Moreover, we used MUMMER (Kurtz *et al.* 2004) and DNADIFF (Phillippy *et al.* 2008) to assess the structural quality of the hybrid and long read assemblies of *Arabidopsis thaliana* (Ler-0) and human (NA12878). The versions and URLs for the scaffolder evaluation and MUMMER are provided in Table S1.

Other software

We used the *k*-mer counter KMC (Kokot *et al.* 2017) to identify the unique *k*-mers in the target contigs. KMC is the only third party software dependency used by FAST-SG. DISCOVAR$_{\text{DENOVO}}$ (Weisenfeld *et al.* 2014) was used to create an Illumina assembly of *Arabidopsis thaliana* (Ler-0) with the purpose of showing the utility of FAST-SG as component of a hybrid assembly pipeline. The versions and URLs for KMC and DISCOVAR$_{\text{DENOVO}}$ are provided in Table S1.

Datasets

Short read datasets

We used the short read datasets provided in Hunt *et al.* (2014) to assess the scaffolding result produced by the short read scaffolders coupled with FAST-SG or short read aligners. The organisms that we used include two bacterial genomes (*Staphylococcus aureus and Rhodobacter sphaeroides*), one fungi (*Plasmodium falciparum*) and the complete human chromosome 14. The URL for the Illumina reads and short read assemblies are provided in Table S2 and Table S4, respectively.

Long read datasets

We used *Escherichia coli K12, Saccharomyces cerevisiae W303* and *Arabidopsis thaliana* (Ler-0) to benchmark the hybrid scaffolding methods.
For *E. coli K12* and *S. cerevisiae W303,* perfect Illumina contigs were created using the tools provided by Hunt *et al.* (2014), in particular, we used the following commands:

```
scaff_test_ctg_to_perfect_ctg.py ecoli_illumina.fa reference.fa artificial_contigs
scaff_test_make_unique_tags.py artificial_contigs.fa reference.fa artificial_contigs.tag
samtools faidx reference.fa
```

where ecoli_illumina.fa is the short read assembly detailed in Table S4 and reference.fa corresponds to NC_000913, the reference genome downloaded from the NCBI database. The same commands were used to build the benchmark files for *S. cerevisiae W303* using the short read assembly detailed in Table S4 and the reference genome GCF_000146045.2, downloaded from the NCBI database. After building the benchmark files, we used the set of reads detailed in Table S3 to build the hybrid scaffolds using LINKS and FAST-SG coupled with the short read scaffolders for each dataset.

For *A. thaliana*, we compared the results obtained by the hybrid method ($DISCOVAR_{DENOVO}$+LORDEC+FAST-SG+the short read scaffolders) against the long read assemblies produced by CANU (Koren *et al.* 2017) and detailed in Table S5. The *A. thaliana* reference genome GCF_000001735.3 was downloaded from the NCBI to validate the quality of the hybrid and long read assemblies using DNADIFF (Phillippy *et al.* 2008). The hybrid assemblies were built for the genome of *A. thaliana* using the long read dataset detailed in Table S3 using various long read coverage.

Nanopore reads and human (NA12878) genome assemblies

The ultra-long nanopore reads (Table S6) produced by Jain *et al.* (2017) for human (NA12878) were downloaded to build an hybrid assembly of the human (NA12878) genome. The results of $DISCOVAR_{DENOVO}$, FAST-SG and SCAFFMATCH were used to generate the hybrid assembly. Moreover, DFS ($DISCOVAR_{DENOVO}$ + FAST-SG + SCAFFMATCH) together with the public assemblies of human (NA12878) produced by 10X genomics (Weisenfeld *et al.* 2017), DOVETAIL genomics (Putnam *et al.* 2016), CANU (Koren *et al.* 2017) and MASURCA (Zimin *et al.* 2017) were downloaded (Table S7) for validation and comparison purposes.

Reference genomes

The reference genomes of *Homo sapiens, Escherichia coli K12, Saccharomyces cerevisiae* and *Arabidopsis thaliana* having NCBI accession numbers GCF_000001405.36, NC_000913, GCF_000146045.2 and GCF_000001735.3 respectively were downloaded from the NCBI to perform various types of benchmarks.

Table S1: Description of the software used within the manuscript.

Program	Type	Version	URL
BOWTIE	Mapper	1.2	https://sourceforge.net/projects/bowtie-bio/files/bowtie/1.2.0/bowtie-1.2-linux-x86_64.zip/download
BOWTIE2	Mapper	2.2.8	https://sourceforge.net/projects/bowtie-bio/files/bowtie2/2.2.8/bowtie2-2.2.8-linux-x86_64.zip/download
BWA	Mapper	0.7.15	https://sourceforge.net/projects/bio-bwa/files/bwa-0.7.15.tar.bz2/download
OPERA-LG	Scaffolder	2.0.6	https://sourceforge.net/projects/operasf/files/OPERA-LG%20version%202.0.6/OPERA-LG_v2.0.6.tar.gz/download
SCAFFMATCH	Scaffolder	0.9	http://alan.cs.gsu.edu/scaffmatch/ScaffMatch-0.9.tar.gz
BESST	Scaffolder	2.0	https://github.com/ksahlin/BESST.git
BOSS	Scaffolder	1.0	https://github.com/bioinfomaticsCSU/BOSS.git
LINKS	Scaffolder	1.8.5	https://github.com/warrenlr/LINKS/blob/master/links_v1-8-5.tar.gz
LORDEC	Hybrid error corrector	0.6	http://www.atgc-montpellier.fr/download/sources/lordec/LoRDEC-0.6.tar.gz
SAMTOOLS	File handling	0.1.19	https://sourceforge.net/projects/samtools/files/samtools/0.1.19/samtools-0.1.19.tar.bz2/download
Scaffolder Evaluation	Benchmark	1.0	https://github.com/martinghunt/Scaffolder-evaluation.git
KMC	Kmer counter	3.0	https://github.com/refresh-bio/KMC.git
DISCOVAR	Assembler	52488	ftp://ftp.broadinstitute.org/pub/crd/DiscovarDeNovo/latest_source_code/discovardenovo-52488.tar.gz
MUMMER	Benchmark	3.23	https://sourceforge.net/projects/mummer/files/mummer/3.23/MUMmer3.23.tar.gz/download

Table S2: Datasets used to perform the short read scaffolding benchmarks.

Organims	#Reads	Read Length	Insert Size	SRA/ENA	URL
S_aureus	3,494,070	37	3,500	SRR022865	http://gage.cbcb.umd.edu/data/Staphylococcus_aureus/Data.original/shortjump_1.fastq.gz, http://gage.cbcb.umd.edu/data/Staphylococcus_aureus/Data.original/shortjump_2.fastq.gz
R_sphaeroides	2,050,868	101	3,500	SRR034528	http://gage.cbcb.umd.edu/data/Rhodobacter_sphaeroides/Data.original/shortjump_1.fastq.gz, http://gage.cbcb.umd.edu/data/Rhodobacter_sphaeroides/Data.original/shortjump_2.fastq.gz
P_falciparum	52,542,302	76	550	ERR034295	ftp://ftp.sra.ebi.ac.uk/vol1/fastq/ERR034/ERR034295/ERR034295_1.fastq.gz, ftp://ftp.sra.ebi.ac.uk/vol1/fastq/ERR034/ERR034295/ERR034295_2.fastq.gz
P_falciparum	1,562,080	75	3,000	ERR163027	ftp://ftp.sra.ebi.ac.uk/vol1/fastq/ERR163/ERR163027/ERR163027_1.fastq.gz, ftp://ftp.sra.ebi.ac.uk/vol1/fastq/ERR163/ERR163027/ERR163027_2.fastq.gz
H_sapiens	22,669,408	101	2,600	SRR067771	http://gage.cbcb.umd.edu/data/Hg_chr14/Data.original/shortjump_1.fastq.gz, http://gage.cbcb.umd.edu/data/Hg_chr14/Data.original/shortjump_2.fastq.gz

Table S3: Datasets used to perform the long read scaffolding benchmarks.

Organism	Chemistry	Type	Machine	#READS	URL
E. coli K12	1D ONT	ONT	R9.2	164,472	https://s3.climb.ac.uk/nanopore/E_coli_K12_1D_R9.2_SpotON_2.pass.fasta
E. coli K12	1.2.1	PacBIO	Sequel System	1,192,955	https://s3.amazonaws.com/files.pacb.com/datasets/secondary-analysis/e-coli-k12-8-plex/Ecoli_8plex_demo.barcoded.subreads.bam
E. coli K12	-	Illumina	Myseq	22,391,084	http://labshare.cshl.edu/shares/schatzlab/www-data/nanocorr/2015.07.07/Ecoli_S1_L001_R1_001.fastq.gz
S. cerevisiae W303	C3	PacBio	PacBio	594,243	http://labshare.cshl.edu/shares/schatzlab/www-data/ectools/w303/Pacbio.fasta.gz
A. thaliana (Ler-0)	1.2.1	PacBIO	Sequel System	561,176	https://downloads.pacbcloud.com/public/SequelData/ArabidopsisDemoData/SequenceData/1_A01_customer/m54113_160913_184949.subreads.bam
A. thaliana (Ler-0)	-	Illumina	Myseq	46,129,480	http://labshare.cshl.edu/shares/schatzlab/www-data/ectools/arabidopsis/Illumina_2x300_R1.fastq.gz, http://labshare.cshl.edu/shares/schatzlab/www-data/ectools/arabidopsis/Illumina_2x300_R2.fastq.gz

Table S4: Illumina assemblies used in the scaffolding benchmarks.

Organism	#CONTIGS	N50	ASM Size (Mb)	#Perfect Contig	Perfect Contig N50	Perfect Contig Size (Mb)	URL
S_aureus	-	-	-	170	47,016	2.85	ftp://ftp.sanger.ac.uk/pub/pathogens/mh12/Scaffolder_evaluation/Scaffolder_evaluation_data.tar.gz
R_sphaeroides	-	-	-	577	15,351	4.48	ftp://ftp.sanger.ac.uk/pub/pathogens/mh12/Scaffolder_evaluation/Scaffolder_evaluation_data.tar.gz
P_falciparum	-	-	-	9,318	2,995	17.82	ftp://ftp.sanger.ac.uk/pub/pathogens/mh12/Scaffolder_evaluation/Scaffolder_evaluation_data.tar.gz
H_sapiens	-	-	-	19,936	12,963	83.68	ftp://ftp.sanger.ac.uk/pub/pathogens/mh12/Scaffolder_evaluation/Scaffolder_evaluation_data.tar.gz
E. coli K12	182	106,405	4.67	140	106,241	4.65	http://labshare.cshl.edu/shares/schatzlab/www-data/ectools/ecoli/ecoli_illumina.fa.gz
S. cerevisiae W303	3,179	48,084	13.21	890	52,324	11.85	http://labshare.cshl.edu/shares/schatzlab/www-data/ectools/w303/w303_illumina.fa.gz

Table S5: CANU long read assemblies of *Arabidopsis thaliana* (Ler-0) genome at different coverage.

Assembler	Coverage	Polishing	URL
CANU	10X	-	http://gembox.cbcb.umd.edu/shared/canu/asm/canu/athal.10X.fasta
CANU	20X	-	http://gembox.cbcb.umd.edu/shared/canu/asm/canu/athal.20X.fasta
CANU	20X	QUIVER	http://gembox.cbcb.umd.edu/shared/canu/quiver/canu/athal.20X.fasta
CANU	20X	PILON	http://gembox.cbcb.umd.edu/shared/canu/pilon/canu/athal.20X.round1.fasta
CANU	50X	-	http://gembox.cbcb.umd.edu/shared/canu/asm/canu/athal.50X.fasta
CANU	150X	-	http://gembox.cbcb.umd.edu/shared/canu/asm/canu/athal.fasta

Table S6: Oxford Nanopore dataset used for the hybrid assembly of the human genome (NA12878).

Flowcell	Number of reads	N50	kit	URL
FAF15665	82,138	114,375	Ultra	http://s3.amazonaws.com/nanopore-human-wgs/rel4-nanopore-wgs-16056159-FAF15665.fastq.gz
FAF13748	53,723	77,045	Ultra	http://s3.amazonaws.com/nanopore-human-wgs/rel4-nanopore-wgs-17958431-FAF13748.fastq.gz
FAF10039	41,385	54,473	Ultra	http://s3.amazonaws.com/nanopore-human-wgs/rel4-nanopore-wgs-2901545329-FAF10039.fastq.gz
FAF09968	19,674	121,393	Ultra	http://s3.amazonaws.com/nanopore-human-wgs/rel4-nanopore-wgs-3439856925-FAF09968.fastq.gz
FAF09277	73,755	117,805	Ultra	http://s3.amazonaws.com/nanopore-human-wgs/rel4-nanopore-wgs-3709819546-FAF09277.fastq.gz
FAF14035	75,692	88,882	Ultra	http://s3.amazonaws.com/nanopore-human-wgs/rel4-nanopore-wgs-3976726082-FAF14035.fastq.gz
FAF15694	61,227	104,528	Ultra	http://s3.amazonaws.com/nanopore-human-wgs/rel4-nanopore-wgs-4109802543-FAF15694.fastq.gz
FAF09713	65,142	93,299	Ultra	http://s3.amazonaws.com/nanopore-human-wgs/rel4-nanopore-wgs-4111860526-FAF09713.fastq.gz
FAF18554	270,189	24,848	Rapid	http://s3.amazonaws.com/nanopore-human-wgs/rel4-nanopore-wgs-4178920553-FAF18554.fastq.gz
FAF15630	9,663	102,804	Ultra	http://s3.amazonaws.com/nanopore-human-wgs/rel4-nanopore-wgs-4244782843-FAF15630.fastq.gz
FAF09640	72,936	92,109	Ultra	http://s3.amazonaws.com/nanopore-human-wgs/rel4-nanopore-wgs-4245291640-FAF09640.fastq.gz
FAF09701	68,169	119,444	Ultra	http://s3.amazonaws.com/nanopore-human-wgs/rel4-nanopore-wgs-4249180049-FAF09701.fastq.gz
FAF15586	71,155	118,548	Ultra	http://s3.amazonaws.com/nanopore-human-wgs/rel4-nanopore-wgs-82266371-FAF15586.fastq.gz
FAF05869	451,020	13,920	Ligation	http://s3.amazonaws.com/nanopore-human-wgs/rel4-nanopore-wgs-87644245-FAF05869.fastq.gz

Table S7: Public human (NA12878) assemblies used for validation and hybrid assembly.

Assembly	URL
DISCOVAR$_{DENOVO}$	ftp://ftp.broadinstitute.org/pub/crd/Discovar/assemblies/51400.newchem/a.lines.fasta
DOVETAIL	ftp://ftp.ncbi.nlm.nih.gov/genomes/all/GCA/001/500/205/GCA_001500205.1_hirise_NA12878_discovar_L1_L2/GCA_001500205.1_hirise_NA12878_discovar_L1_L2_genomic.fna.gz
10X	http://cf.10xgenomics.com/samples/assembly/1.1.0/msNA12878/msNA12878_pseudohap.fasta.gz
CANU-P	http://s3.amazonaws.com/nanopore-human-wgs/canu.35x.contigs.polished2.fasta
MASURCA	ftp://ftp.genome.umd.edu/pub/NA12878/assembly.7xlong.fa

Supplementary Material 2: Long read scaffolding benchmark.

Long read error correction

The long reads were error corrected using LORDEC. For *E. coli K12*, we sampled a total of 50X of coverage from PacBio (Sequel system) and Oxford Nanopore (1D). These reads were error-corrected using the Illumina reads described in Table S3: Datasets used to perform the long read scaffolding benchmarks.Table S3. We repeated the same procedure to error-correct the 50X of PacBio reads of *A. thaliana* (Ler-0) (Table S3). The LORDEC commands used for each dataset were the following:

E. coli K12

```
#PacBio reads
time ${LORDEC} -T 32 -i ECOLI-PACSEQ.200Mb.fasta.gz  -2 Ecoli_S1_L001_R1_001.fastq.gz -k 17 -
o ECOLI-PACSEQ.200Mb.k17s3.lordec -s 3
#ONT 1D reads
time ${LORDEC} -T 32 -i ECOLI-K12-1D.200Mb.fasta.gz -2 Ecoli_S1_L001_R1_001.fastq.gz -k 17 -o
ECOLI-K12-1D.200Mb.k17s3.lordec -s 3
```

A. thaliana (Ler-0)

```
time ${LORDEC} -T 20 -i PACSEQ.50X.fasta.gz  -2 Illumina_2x300_R1.fastq.gz,
Illumina_2x300_R1.fastq.gz -k 17 -o PACSEQ.50X.k17s3.lordec -s 3
```

Long read scaffolding

We compared FAST-SG coupled with the short read scaffolders against LINKS, a scaffolder designed for extracting pairs of *k*-mers from the long reads and used them to join contigs. In particular, we used the *E. coli K12* and *S. cerevisiae W303* datasets for comparison against LINKS. The *A. thaliana* dataset was used to compare the performance of a hybrid assembly strategy against the long read assembler CANU. BESST2 was not used for long read scaffolding due to an exception produced by a division by zero while BESST2 computes the average contig coverage from synthetic mate pair libraries.

E.coli K12 dataset

For *E. coli K12,* we used four different sets of long reads: ONT 1D raw, ONT 1D corrected, PACBIO raw and PACBIO corrected. The command settings for FAST-SG and the short read scaffolders were the following:

FAST-SG command:

```
time -v -o time-fast-sg.time ./FAST-SG.pl -k ${K} -l lib-long-corrected.txt -r ${CTG}.fa -p ${OUT}
Where ${K} is 15-mer, FAST-SG.pl is wrapper script to execute FAST-SG, ${CTG}.fa is the set
of contigs, ${OUT} is the output prefix and lib-long-corrected.txt is the configuration file
with long read details:
#type, lib-label, reads-path, target-insert sizes, sam output(0=paired, 1=unpaired).
long long_pac_cor reads/ECOLI-PACSEQ.200Mb.k17s3.lordec.rename.gz
500,1000,2000,3000,4000,5000,6000,7000,8000 1
long long_pac_raw reads/ECOLI-PACSEQ.200Mb.fasta.rename.gz
500,1000,2000,3000,4000,5000,6000,7000,8000 1
long long_ont_raw reads/ECOLI-K12-1D.200Mb.fasta.rename.gz
500,1000,2000,3000,4000,5000,6000,7000,8000 1
long long_ont_cor reads/ECOLI-K12-1D.200Mb.k17s3.lordec.rename.gz
500,1000,2000,3000,4000,5000,6000,7000,8000 1
```

Short read scaffolder commands:
We show the command details for the ONT 1D reads only. However, identical commands were used for the other long read sets, changing only the input files according to each set. The Linux time utility was used to measure the elapsed time of each command.

SCAFFMATCH

```
scaffmatch -m -w SM_ONTR-K${K} -c ${IN}.fa -1 long_ont_raw.I500.Fast-
SG_K${K}.fwd.sam,long_ont_raw.I1000.Fast-SG_K${K}.fwd.sam,long_ont_raw.I2000.Fast-
SG_K${K}.fwd.sam,long_ont_raw.I3000.Fast-SG_K${K}.fwd.sam,long_ont_raw.I4000.Fast-
SG_K${K}.fwd.sam,long_ont_raw.I5000.Fast-SG_K${K}.fwd.sam,long_ont_raw.I6000.Fast-
SG_K${K}.fwd.sam,long_ont_raw.I7000.Fast-SG_K${K}.fwd.sam,long_ont_raw.I8000.Fast-
SG_K${K}.fwd.sam -2 long_ont_raw.I500.Fast-SG_K${K}.rev.sam,long_ont_raw.I1000.Fast-
SG_K${K}.rev.sam,long_ont_raw.I2000.Fast-SG_K${K}.rev.sam,long_ont_raw.I3000.Fast-
SG_K${K}.rev.sam,long_ont_raw.I4000.Fast-SG_K${K}.rev.sam,long_ont_raw.I5000.Fast-
SG_K${K}.rev.sam,long_ont_raw.I6000.Fast-SG_K${K}.rev.sam,long_ont_raw.I7000.Fast-
SG_K${K}.rev.sam,long_ont_raw.I8000.Fast-SG_K${K}.rev.sam -i
441,965,2016,3066,4115,5165,6216,7268,8318 -p fr,fr,fr,fr,fr,fr,fr,fr,fr -s
50,100,200,300,400,500,600,700,800
```

OPERA-LG

```
#OPERA-LG conf file:
output_folder=opera-long_ont_raw-K15
contig_file=ecoli-ill.fa
filter_repeat=yes
haploid_coverage=50
[LIB]
map_file=long_ont_raw.I500.Fast-SG_K15.sam
[LIB]
map_file=long_ont_raw.I1000.Fast-SG_K15.sam
[LIB]
map_file=long_ont_raw.I2000.Fast-SG_K15.sam
[LIB]
map_file=long_ont_raw.I3000.Fast-SG_K15.sam
[LIB]
map_file=long_ont_raw.I4000.Fast-SG_K15.sam
[LIB]
map_file=long_ont_raw.I5000.Fast-SG_K15.sam
[LIB]
map_file=long_ont_raw.I6000.Fast-SG_K15.sam
[LIB]
map_file=long_ont_raw.I7000.Fast-SG_K15.sam
[LIB]
map_file=long_ont_raw.I8000.Fast-SG_K15.sam
#OPERA-LG command:
${OPERA-DIR}/OPERA-LG opera.K${K}.ont_raw.conf
```

BOSS

```
boss ${IN}.fa long_ont_raw.I500.Fast-SG_K15.fwd.bam long_ont_raw.I500.Fast-SG_K15.rev.bam 75
441 0.1 0.2 5 1 0  long_ont_raw.I1000.Fast-SG_K15.fwd.bam long_ont_raw.I1000.Fast-
SG_K15.rev.bam 75 965 0.1 0.2 5 1 0  long_ont_raw.I2000.Fast-SG_K15.fwd.bam
long_ont_raw.I2000.Fast-SG_K15.rev.bam 75 2016 0.1 0.2 5 1 0  long_ont_raw.I3000.Fast-
SG_K15.fwd.bam long_ont_raw.I3000.Fast-SG_K15.rev.bam 75 3066 0.1 0.2 5 1 0
long_ont_raw.I4000.Fast-SG_K15.fwd.bam long_ont_raw.I4000.Fast-SG_K15.rev.bam 75 4115 0.1 0.2
5 1 0  long_ont_raw.I5000.Fast-SG_K15.fwd.bam long_ont_raw.I5000.Fast-SG_K15.rev.bam 75 5165
0.1 0.2 5 1 0  long_ont_raw.I6000.Fast-SG_K15.fwd.bam long_ont_raw.I6000.Fast-SG_K15.rev.bam
75 6216 0.1 0.2 5 1 0  long_ont_raw.I7000.Fast-SG_K15.fwd.bam long_ont_raw.I7000.Fast-
SG_K15.rev.bam 75 7268 0.1 0.2 5 1 0  long_ont_raw.I8000.Fast-SG_K15.fwd.bam
long_ont_raw.I8000.Fast-SG_K15.rev.bam 75 8318 0.1 0.2 5 1 0  B2ONTR-K${K}
```

LINKS commands:

```
#ONT 1D RAW
time -v -o time_links_ont_raw.txt ${LINKS} -f ecoli-ill.fa -s K12_ONT.raw -d
500,1000,2000,3000,4000,5000,6000,7000,8000 -b ecoli-ont-raw
#PACBIO RAW
time -v -o time_links_pac_raw.txt ${LINKS} -f ecoli-ill.fa -s K12_PAC.raw -d
500,1000,2000,3000,4000,5000,6000,7000,8000 -b ecoli-pac-raw
```

S. cerevisiae W303 dataset

FAST-SG command:

```
time -v -o time-fast-sg.time ./FAST-SG.pl -k ${K} -l lib-long-corrected.txt -r ${CTG}.fa -p ${OUT}
Where ${K} is 15-mer, FAST-SG.pl is wrapper script to execute FAST-SG,  ${CTG}.fa is the set
of contigs, ${OUT} is the output prefix and lib-long-corrected.txt is the configuration file
with long read details:
#type, lib-label, reads-path, target insert sizes, sam output(0=paired, 1=unpaired).
long long_pac_raw reads/Pacbio.30X.rename.fasta.gz
500,1000,2000,3000,4000,5000,6000,7000,8000 1
```

Short read scaffolders commands:
We executed the commands in a similar way as for the *E. coli* k12 dataset (changing only the input SAM/BAM and the contigs) to build the scaffolds with the short read scaffolders.

LINKS commands:

```
#PACBIO raw
time -v -o time_links_pac_raw.txt ${LINKS} -f Scer-w303.fa -s SCER_PAC.raw -d
500,1000,2000,3000,4000,5000,6000,7000,8000 -b Scer-pac-raw
```

A. *thaliana* (Ler-0) dataset

We produced an Illumina assembly of *A. thaliana* using DISCOVAR$_{DENOVO}$ with the following command:

```
time -v -o time_disco2_300bp_ill.txt DiscovarDeNovo
READS="Illumina_2x300_R1.fastq.gz,Illumina_2x300_R2.fastq.gz" OUT_DIR=ler-disco
NUM_THREADS=20 MAX_MEM_GB=700
```

The long reads were then error-corrected using LORDEC as indicated in the previous section. After error-correction, we used FAST-SG at various long read coverages, with the following configuration:

```
time -v -o time-fast-sg.time ./FAST-SG.pl -k ${K} -l lib-long-corrected.txt -r ${CTG}.fa -p ${OUT} -t 20
Where ${K} is 21-mer, FAST-SG.pl is wrapper script to execute FAST-SG,  ${CTG}.fa is the set
of contigs, ${OUT} is the output prefix, -t 20 is 20 CPUs and lib-long-corrected.txt is the
configuration file with long read details:
#lib-label, reads-path, target insert sizes, sam output(0=paired, 1=unpaired).
long long_pac_cor_5X reads/PACSEQ.5X.lordec.rename.fasta.gz
1000,2000,3000,4000,5000,6000,7000,8000,10000,12000,15000,20000 1
long long_pac_cor_10X reads/PACSEQ.10X.lordec.rename.fasta.gz
1000,2000,3000,4000,5000,6000,7000,8000,10000,12000,15000,20000 1
long long_pac_cor_15X reads/PACSEQ.15X.lordec.rename.fasta.gz
1000,2000,3000,4000,5000,6000,7000,8000,10000,12000,15000,20000 1
long long_pac_cor_20X reads/PACSEQ.20X.lordec.rename.fasta.gz
1000,2000,3000,4000,5000,6000,7000,8000,10000,12000,15000,20000 1
long long_pac_cor_30X reads/PACSEQ.30X.lordec.rename.fasta.gz
1000,2000,3000,4000,5000,6000,7000,8000,10000,12000,15000,20000 1
long long_pac_cor_50X reads/PACSEQ.50X.k17s3.lordec.gz.rename.gz
1000,2000,3000,4000,5000,6000,7000,8000,10000,12000,15000,20000 1
```

Short read scaffolder commands:
We ran the short read scaffolders with different subsamples of long read coverage. We show the command used for 5X of long read coverage, but identical commands were used for the other subsamples. The commands used were the following:

SCAFFMATCH

```
scaffmatch -m -w SM2PACC-K${K}-5X -c ${IN}.fa -s
100,200,300,400,500,600,700,800,1000,1200,1500,2000 -i
1000,2000,3000,4000,5000,6000,7000,8000,10000,12000,15000,20000 -p
fr,fr,fr,fr,fr,fr,fr,fr,fr,fr,fr,fr -1 long_pac_cor_5X.I1000.Fast-
```

```
SG_K${K}.fwd.sam,long_pac_cor_5X.I2000.Fast-SG_K${K}.fwd.sam,long_pac_cor_5X.I3000.Fast-
SG_K${K}.fwd.sam,long_pac_cor_5X.I4000.Fast-SG_K${K}.fwd.sam,long_pac_cor_5X.I5000.Fast-
SG_K${K}.fwd.sam,long_pac_cor_5X.I6000.Fast-SG_K${K}.fwd.sam,long_pac_cor_5X.I7000.Fast-
SG_K${K}.fwd.sam,long_pac_cor_5X.I8000.Fast-SG_K${K}.fwd.sam,long_pac_cor_5X.I10000.Fast-
SG_K${K}.fwd.sam,long_pac_cor_5X.I12000.Fast-SG_K${K}.fwd.sam,long_pac_cor_5X.I15000.Fast-
SG_K${K}.fwd.sam,long_pac_cor_5X.I20000.Fast-SG_K${K}.fwd.sam -2 long_pac_cor_5X.I1000.Fast-
SG_K${K}.rev.sam,long_pac_cor_5X.I2000.Fast-SG_K${K}.rev.sam,long_pac_cor_5X.I3000.Fast-
SG_K${K}.rev.sam,long_pac_cor_5X.I4000.Fast-SG_K${K}.rev.sam,long_pac_cor_5X.I5000.Fast-
SG_K${K}.rev.sam,long_pac_cor_5X.I6000.Fast-SG_K${K}.rev.sam,long_pac_cor_5X.I7000.Fast-
SG_K${K}.rev.sam,long_pac_cor_5X.I8000.Fast-SG_K${K}.rev.sam,long_pac_cor_5X.I10000.Fast-
SG_K${K}.rev.sam,long_pac_cor_5X.I12000.Fast-SG_K${K}.rev.sam,long_pac_cor_5X.I15000.Fast-
SG_K${K}.rev.sam,long_pac_cor_5X.I20000.Fast-SG_K${K}.rev.sam
```

OPERA-LG

```
#OPERA-LG configuration file:
output_folder=opera-long_pac_cor_5X-K21
contig_file=ler-disco.1kb.fa
filter_repeat=yes
haploid_coverage=15
[LIB]
map_file=long_pac_cor_5X.I1000.Fast-SG_K21.sam
[LIB]
map_file=long_pac_cor_5X.I2000.Fast-SG_K21.sam
[LIB]
map_file=long_pac_cor_5X.I3000.Fast-SG_K21.sam
[LIB]
map_file=long_pac_cor_5X.I4000.Fast-SG_K21.sam
[LIB]
map_file=long_pac_cor_5X.I5000.Fast-SG_K21.sam
[LIB]
map_file=long_pac_cor_5X.I6000.Fast-SG_K21.sam
[LIB]
map_file=long_pac_cor_5X.I7000.Fast-SG_K21.sam
[LIB]
map_file=long_pac_cor_5X.I8000.Fast-SG_K21.sam
[LIB]
map_file=long_pac_cor_5X.I10000.Fast-SG_K21.sam
[LIB]
map_file=long_pac_cor_5X.I12000.Fast-SG_K21.sam
[LIB]
map_file=long_pac_cor_5X.I15000.Fast-SG_K21.sam
[LIB]
map_file=long_pac_cor_5X.I20000.Fast-SG_K21.sam
#OPERA-LG command
${OPERA-DIR}/OPERA-LG opera.K${K}.5X.pac_cor.conf
```

BOSS

```
boss ${IN}.fa long_pac_cor_5X.I1000.Fast-SG_K${K}.fwd.bam long_pac_cor_5X.I1000.Fast-
SG_K${K}.rev.bam 75 868 0.1 0.2 5 1 0  long_pac_cor_5X.I2000.Fast-SG_K${K}.fwd.bam
long_pac_cor_5X.I2000.Fast-SG_K${K}.rev.bam 75 1826 0.1 0.2 5 1 0
long_pac_cor_5X.I3000.Fast-SG_K${K}.fwd.bam long_pac_cor_5X.I3000.Fast-SG_K${K}.rev.bam 75
2783 0.1 0.2 5 1 0  long_pac_cor_5X.I4000.Fast-SG_K${K}.fwd.bam long_pac_cor_5X.I4000.Fast-
SG_K${K}.rev.bam 75 3739 0.1 0.2 5 1 0  long_pac_cor_5X.I5000.Fast-SG_K${K}.fwd.bam
long_pac_cor_5X.I5000.Fast-SG_K${K}.rev.bam 75 4695 0.1 0.2 5 1 0
long_pac_cor_5X.I6000.Fast-SG_K${K}.fwd.bam long_pac_cor_5X.I6000.Fast-SG_K${K}.rev.bam 75
5652 0.1 0.2 5 1 0  long_pac_cor_5X.I7000.Fast-SG_K${K}.fwd.bam long_pac_cor_5X.I7000.Fast-
SG_K${K}.rev.bam 75 6609 0.1 0.2 5 1 0  long_pac_cor_5X.I8000.Fast-SG_K${K}.fwd.bam
long_pac_cor_5X.I8000.Fast-SG_K${K}.rev.bam 75 7562 0.1 0.2 5 1 0
long_pac_cor_5X.I10000.Fast-SG_K${K}.fwd.bam long_pac_cor_5X.I10000.Fast-SG_K${K}.rev.bam 75
9466 0.1 0.2 5 1 0  long_pac_cor_5X.I12000.Fast-SG_K${K}.fwd.bam long_pac_cor_5X.I12000.Fast-
SG_K${K}.rev.bam 75 11359 0.1 0.2 5 1 0  long_pac_cor_5X.I15000.Fast-SG_K${K}.fwd.bam
long_pac_cor_5X.I15000.Fast-SG_K${K}.rev.bam 75 14213 0.1 0.2 5 1 0
long_pac_cor_5X.I20000.Fast-SG_K${K}.fwd.bam long_pac_cor_5X.I20000.Fast-SG_K${K}.rev.bam 75
18974 0.1 0.2 5 1 0   B2PACC-K${K}-5X
```

Long read scaffolding of human (NA12878)

Using as input the ultra-long Nanopore reads detailed in Table S6 and the DISCOVAR$_{DENOVO}$ assembly of human (NA12878) (Table S7), we generated a hybrid assembly of human (NA12878) by complementing the DISCOVAR$_{DENOVO}$ and long reads with FAST-SG and SCAFFMATCH. The commands were the following:

```
time -v -o time-fast-sg.time ./FAST-SG.pl -k ${K} -l lib-long-corrected.txt -r ${CTG}.fa -p ${OUT} -t 20
Where ${K} is 22-mer, FAST-SG.pl is wrapper script to execute FAST-SG,  ${CTG}.fa is the set
of contigs, ${OUT} is the output prefix, -t 20 is 20 CPUs and lib-long.txt is the
configuration file with long read details:
#type lib-label, reads-path, target insert sizes, sam output(0=paired, 1=unpaired).
long ultra_ont_raw ULTRA-LONG-RENAME-FOLD.fa.gz
2000,4000,6000,8000,10000,12000,14000,16000,18000,20000,30000,40000,50000,60000,70000,80000,1
00000,120000,150000,180000 1
```

FAST-SG was used to create synthetic mate-pair libraries in the range of 2kb to 180kb. The SCAFFMATCH command used was then the following:

```
./time -v -o time-sm-sg${K}.time ./scaffmatch -m -w UONT-K${K}-7X -c ${IN}.fa -s
200,416,632,847,1063,1278,1494,1709,1924,2140,3214,4286,5356,6424,7487,8548,10668,12778,15952
,19139 -i
2009,4164,6320,8473,10630,12786,14941,17099,19249,21404,32146,42865,53561,64240,74875,85482,1
06685,127782,159520,191398 -p fr,fr,fr,fr,fr,fr,fr,fr,fr,fr,fr,fr,fr,fr,fr,fr,fr,fr,fr,fr -1
ultra_ont_raw.I2000.Fast-SG_K${K}.fwd.sam,ultra_ont_raw.I4000.Fast-
SG_K${K}.fwd.sam,ultra_ont_raw.I6000.Fast-SG_K${K}.fwd.sam,ultra_ont_raw.I8000.Fast-
SG_K${K}.fwd.sam,ultra_ont_raw.I10000.Fast-SG_K${K}.fwd.sam,ultra_ont_raw.I12000.Fast-
SG_K${K}.fwd.sam,ultra_ont_raw.I14000.Fast-SG_K${K}.fwd.sam,ultra_ont_raw.I16000.Fast-
SG_K${K}.fwd.sam,ultra_ont_raw.I18000.Fast-SG_K${K}.fwd.sam,ultra_ont_raw.I20000.Fast-
SG_K${K}.fwd.sam,ultra_ont_raw.I30000.Fast-SG_K${K}.fwd.sam,ultra_ont_raw.I40000.Fast-
SG_K${K}.fwd.sam,ultra_ont_raw.I50000.Fast-SG_K${K}.fwd.sam,ultra_ont_raw.I60000.Fast-
SG_K${K}.fwd.sam,ultra_ont_raw.I70000.Fast-SG_K${K}.fwd.sam,ultra_ont_raw.I80000.Fast-
SG_K${K}.fwd.sam,ultra_ont_raw.I100000.Fast-SG_K${K}.fwd.sam,ultra_ont_raw.I120000.Fast-
SG_K${K}.fwd.sam,ultra_ont_raw.I150000.Fast-SG_K${K}.fwd.sam,ultra_ont_raw.I180000.Fast-
SG_K${K}.fwd.sam -2 ultra_ont_raw.I2000.Fast-SG_K${K}.rev.sam,ultra_ont_raw.I4000.Fast-
SG_K${K}.rev.sam,ultra_ont_raw.I6000.Fast-SG_K${K}.rev.sam,ultra_ont_raw.I8000.Fast-
SG_K${K}.rev.sam,ultra_ont_raw.I10000.Fast-SG_K${K}.rev.sam,ultra_ont_raw.I12000.Fast-
SG_K${K}.rev.sam,ultra_ont_raw.I14000.Fast-SG_K${K}.rev.sam,ultra_ont_raw.I16000.Fast-
SG_K${K}.rev.sam,ultra_ont_raw.I18000.Fast-SG_K${K}.rev.sam,ultra_ont_raw.I20000.Fast-
SG_K${K}.rev.sam,ultra_ont_raw.I30000.Fast-SG_K${K}.rev.sam,ultra_ont_raw.I40000.Fast-
SG_K${K}.rev.sam,ultra_ont_raw.I50000.Fast-SG_K${K}.rev.sam,ultra_ont_raw.I60000.Fast-
SG_K${K}.rev.sam,ultra_ont_raw.I70000.Fast-SG_K${K}.rev.sam,ultra_ont_raw.I80000.Fast-
SG_K${K}.rev.sam,ultra_ont_raw.I100000.Fast-SG_K${K}.rev.sam,ultra_ont_raw.I120000.Fast-
SG_K${K}.rev.sam,ultra_ont_raw.I150000.Fast-SG_K${K}.rev.sam,ultra_ont_raw.I180000.Fast-
SG_K${K}.rev.sam
```

Insert sizes and standard deviation for each synthetic library were extracted from the FAST-SG log file and were used as parameters for SCAFFMATCH.

Long read scaffolding validation

E. coli K12 dataset

We assessed the quality of the scaffold sequences using the standard short read scaffolding evaluation tools as described previously. The command was the following:

```
scaff_test_check_using_tags.py --all_circular 8000  artificial_contigs.tag ${SCAFFOLDS}
reference.fa.fai ${SCFFOLDS}.check
```

S. cerevisiae W303 dataset

We assessed the quality of the scaffold sequences using the standard short read scaffolding evaluation tools as described previously. The command was the following:

```
scaff_test_check_using_tags.py --all_circular 8000  artificial_contigs.tag ${SCAFFOLDS}
reference.fa.fai ${SCFFOLDS}.check
```

A. thaliana (Ler-0) dataset

We used NUCMER and DNADIFF to assess the structural quality of the hybrid and long read assemblies, with the following commands:

```
nucmer -l 100 -c 1000 -p ${IN} GCF_000001735.3_TAIR10_genomic.rename.fna ${IN}.fna
dnadiff -d ${IN}.delta -p ${IN}_dnadiff
```

Human (NA12878) dataset

We used NUCMER and DNADIFF to assess the structural quality of the hybrid assembly produced by DISCOVAR+FAST-SG+SCAFFMATCH together with the human (NA12878) genome assemblies described in Table S7, with the following command:

```
nucmer -l 100 -c 500 -maxmatch -p ${IN} GRCh38_latest_genomic.format.fna ${IN}.fna
dnadiff -d ${IN}.delta -p ${IN}_dnadiff
```

Synthetic libraries and comparison of FAST-SG against LINKS

Table S8: FAST-SG recall at *k*-mer and read level on synthetic mate-pair libraries extracted from corrected or uncorrected long reads using the *E. coli* K12 dataset.

**Long reads**	**Total look-ups *k*-mers**	**Total matched *k*-mers**	**Total created read pairs**	**Total aligned read pairs**	***k*-mer Recall**	**Read pair Recall**
ONT_RAW	2,455,571,885	205,169,028	12,399,445	6,128,733	8.355	49.427
ONT_COR	1,261,459,649	240,433,478	13,039,178	9,795,813	19.060	75.126
PAC_RAW	1,690,781,384	85,717,526	6,821,765	2,159,282	5.070	31.653
PAC_COR	766,721,500	112,530,485	6,632,298	4,312,646	14.677	65.025

Table S9: Long read datasets used for comparison against LINKS.

The elapsed time for index construction and alignment was obtained from the log files produced by both programs. The memory peak was obtained from the GNU time utility. For LINKS, we consider the time spent in the alignment step only.

Dataset	Long reads	Number of long reads	Min length	Max length	N50	Total length	Genome coverage	Software	Index and alignment time (s)	Memory Peak (Gb)
E.coli K12	PacBio	48,425	500	34,843	5,350	209,568,152	50X	LINKS	4,983	34.229
								FAST-SG	2,727	0.106
	ONT	23,298	267	105,642	14,781	209,561,263	50X	LINKS	9,455	43.486
								FAST-SG	4,221	0.106
S. cerevisiae W303	PacBio	73,015	500	35,846	6,609	364,696,529	30X	LINKS	11,944	56.493
								FAST-SG	5,359	0.141

Table S10: Number of *k*-mer pairs and read pairs extracted from raw long reads by LINKS and FAST-SG. The percentage of linking *k*-mer pairs and linking read-pairs is detailed for each synthetic library.

For LINKS, the total number of *k*-mer pairs aligned and linking *k*-mer pairs were obtained from the log files. For FAST-SG, the number of read pairs and linking pairs were counted from the SAM files.

Synthetic Libraries	*E. coli K12*				*S .cerevisiae W303*	
	ONT		PacBio		PacBio	
	Total	%Linking	Total	%Linking	Total	%Linking
LINKS (*k*-mer pairs counts)						
500	5,891,840	0.013	1,725,058	0.009	3,978,076	0.449
1000	5,513,218	0.086	1,493,576	0.062	3,701,899	1.097
2000	4,922,777	1.251	1,091,702	1.104	3,167,802	2.587
3000	4,417,107	2.624	748,450	2.400	2,699,967	4.195
4000	3,942,909	4.129	475,890	3.570	2,269,181	5.706
5000	3,501,104	5.608	283,139	4.643	1,891,323	7.395
6000	3,133,391	7.102	156,532	5.667	1,564,795	8.992
7000	2,818,742	8.692	83,805	6.513	1,280,920	10.638
8000	2,544,728	10.152	43,507	7.624	1,039,493	12.441
**Total**	36,685,816	3.457	6,101,659	1.293	21,593,456	4.304
FAST-SG (read pairs counts)						
500	918,651	0.027	544,843	0.034	523,664	0.431
1000	938,720	0.331	549,924	0.218	582,492	1.413
2000	839,101	2.228	404,952	1.640	503,272	4.020
3000	747,432	4.080	277,164	3.313	427,111	6.456
4000	664,374	5.790	176,322	4.894	358,568	8.696
5000	591,746	7.525	103,543	6.464	297,799	10.957
6000	528,779	9.180	57,069	7.964	244,978	13.359
7000	474,232	10.851	30,179	9.808	199,073	15.590
8000	425,698	12.504	15,286	11.736	160,532	17.778
**Total**	6,128,733	4.712	2,159,282	1.937	3,297,489	6.502

Table S11: Long read scaffolding benchmark results for *E. coli* K12 and *S. cerevisiae* W303. FAST-SG coupled with short read scaffolders was compared against LINKS.

Reads	Dataset	Scaffolder	Tags	Total joins	Correct joins	% correct	Potential joins	Recall	1	2	4	5	8	12	Bad joins	% Errors	TPR	PPV	F-Score
ONT-RAW	*E. coli* K12	OPERA-LG	140	76	70	92.11	139	50.36	0	0	1	2	0	3	6	7.89	0.504	0.921	0.651
PAC-RAW	*E. coli* K12	OPERA-LG	140	77	70	90.91	139	50.36	2	0	1	2	0	2	7	9.09	0.504	0.909	0.648
ONT-RAW	*E. coli* K12	SCAFFMATCH	140	102	92	90.20	139	66.19	0	0	2	4	0	4	10	9.80	0.662	0.902	0.763
PAC-RAW	*E. coli* K12	SCAFFMATCH	140	93	80	86.02	139	57.55	2	0	5	2	0	4	13	13.98	0.576	0.860	0.690
ONT-RAW	*E. coli* K12	BOSS	140	88	81	92.05	139	58.27	0	0	2	2	1	2	7	7.95	0.583	0.920	0.714
PAC-RAW	*E. coli* K12	BOSS	140	83	77	92.77	139	55.40	0	0	2	2	1	1	6	7.23	0.554	0.928	0.694
ONT-RAW	*E. coli* K12	LINKS	140	85	80	94.12	139	57.55	0	0	1	3	0	1	5	5.88	0.576	0.941	0.714
PAC-RAW	*E. coli* K12	LINKS	140	79	68	86.08	139	48.92	0	0	2	7	0	2	11	13.92	0.489	0.861	0.624
PAC-RAW	*S. cerevisiae* W303	OPERA-LG	890	353	327	92.63	889	36.78	0	20	3	0	2	1	26	7.37	0.368	0.926	0.527
PAC-RAW	*S. cerevisiae* W303	SCAFFMATCH	890	399	356	89.22	889	40.04	0	38	2	0	2	1	43	10.78	0.400	0.892	0.553
PAC-RAW	*S. cerevisiae* W303	BOSS	890	370	342	92.43	889	38.47	0	26	2	0	0	0	28	7.57	0.385	0.924	0.543
PAC-RAW	*S. cerevisiae* W303	LINKS	890	401	350	87.28	889	39.37	5	39	4	1	1	1	51	12.72	0.394	0.873	0.543

Supplementary Material 3: *Arabidopsis thaliana* (Ler-0) and human (NA12878) hybrid genome assemblies.

FAST-SG alignments

Table S12 and Table S13 provide details on the number of read-pairs and linking pairs obtained by using FAST-SG to scaffold the DISCOVAR$_{DENOVO}$ assemblies of *Arabidopsis thaliana* (Ler-0) and human (NA12878), respectively.

Synthetic libraries

Figure S1 and Figure S2 provide details on the insert sizes distribution observed by each synthetic library for the hybrid scaffolding of the DISCOVAR$_{DENOVO}$ assemblies of *Arabidopsis thaliana* (Ler-0) and human (NA12878). Insert sizes were computed using pairs aligned within contigs.

Structural errors

Figure S3 and Figure S4 show the number and amount of bases involved in structural errors for the *Arabidopsis thaliana* (Ler-0) and human (NA12878) assemblies, respectively.

Table S14 provides particular examples of short contigs skipped in the hybrid scaffolding of the human (NA12878) and Table S15 provides examples of quimeric contigs from CANU and MASURCA.

Table S12: Number of synthetic read pairs aligned to the DISCOVAR$_{DENOVO}$ assembly of *Arabidopsis thaliana* (Ler-0) at various subsamples of long read coverage and percentage of linking pairs by synthetic library.

Insert Size	5X		10X		15X		20X		30X		50X	
	Pairs (M)	Linking (%)	Pairs (M)	Linking (%)	Pairs (M)	Linking (%)	Pairs (M)	Linking (%)	Pairs (M)	Linking (%)	Pairs (M)	Linking (%)
1kb	1.85	3.63	3.66	3.65	5.48	3.66	7.33	3.66	10.99	3.65	16.32	3.65
2kb	1.67	4.62	3.31	4.64	4.95	4.65	6.62	4.65	9.93	4.64	14.73	4.64
3kb	1.49	5.69	2.95	5.70	4.42	5.71	5.92	5.72	8.88	5.73	13.17	5.71
4kb	1.33	6.67	2.63	6.66	3.94	6.68	5.27	6.68	7.92	6.69	11.74	6.66
5kb	1.19	7.81	2.35	7.75	3.52	7.79	4.71	7.79	7.07	7.81	10.48	7.75
6kb	1.05	8.68	2.09	8.62	3.13	8.68	4.19	8.70	6.30	8.73	9.32	8.66
7kb	0.93	9.28	1.85	9.23	2.77	9.31	3.71	9.33	5.58	9.35	8.25	9.28
8kb	0.82	9.97	1.63	9.89	2.44	9.96	3.28	9.98	4.93	10.03	7.29	9.93
10kb	0.63	11.11	1.26	11.06	1.89	11.16	2.54	11.18	3.82	11.22	5.63	11.11
12kb	0.48	12.22	0.95	12.20	1.43	12.33	1.92	12.32	2.90	12.37	4.27	12.24
15kb	0.30	13.67	0.60	13.63	0.90	13.80	1.22	13.77	1.84	13.83	2.70	13.68
20kb	0.11	14.75	0.23	14.86	0.35	15.12	0.48	15.07	0.74	15.12	1.07	14.94
Total	11.85	7.23	23.50	7.21	35.21	7.26	47.18	7.27	70.89	7.29	104.99	7.24

Table S13: Number of synthetic read pairs aligned to the human (NA12878) DISCOVAR$_{DENOVO}$ assembly and percentage of linking pairs by library.

Insert-size	Linking	Within	Total pairs	% Linking
2kb	227,690	46,260,555	46,488,245	0.49
4kb	591,432	41,354,625	41,946,057	1.41
6kb	939,382	37,720,193	38,659,575	2.43
8kb	1,311,609	34,857,814	36,169,423	3.63
10kb	1,643,495	32,459,362	34,102,857	4.82
12kb	1,931,570	30,345,590	32,277,160	5.98
14kb	2,196,953	28,477,799	30,674,752	7.16
16kb	2,431,767	26,798,691	29,230,458	8.32
18kb	2,640,335	25,317,369	27,957,704	9.44
20kb	2,838,925	23,997,333	26,836,258	10.58
30kb	3,537,015	18,621,314	22,158,329	15.96
40kb	3,897,320	14,585,742	18,483,062	21.09
50kb	4,038,956	11,636,924	15,675,880	25.77
60kb	4,049,515	9,375,053	13,424,568	30.16
70kb	3,971,701	7,589,360	11,561,061	34.35
80kb	3,837,227	6,180,553	10,017,780	38.30
100kb	3,472,770	4,155,751	7,628,521	45.52
120kb	3,044,914	2,820,602	5,865,516	51.91
150kb	2,402,226	1,608,269	4,010,495	59.90
180kb	1,835,887	924,098	2,759,985	66.52
Total	50,840,689	405,086,997	455,927,686	11.15

Figure S1: Boxplot of synthetic libraries extracted by FAST-SG (K21) from the PacBio reads to scaffold the *Arabidopsis thaliana* (Ler-0) genome.

The boxplot was drawn using a total of 900,000 insert sizes from read pairs mapped within contigs for each synthetic library. The percentage of outliers detected ranged from a minimum of 0.68% and a maximum of 13.12% for 1kb and 20kb respectively.

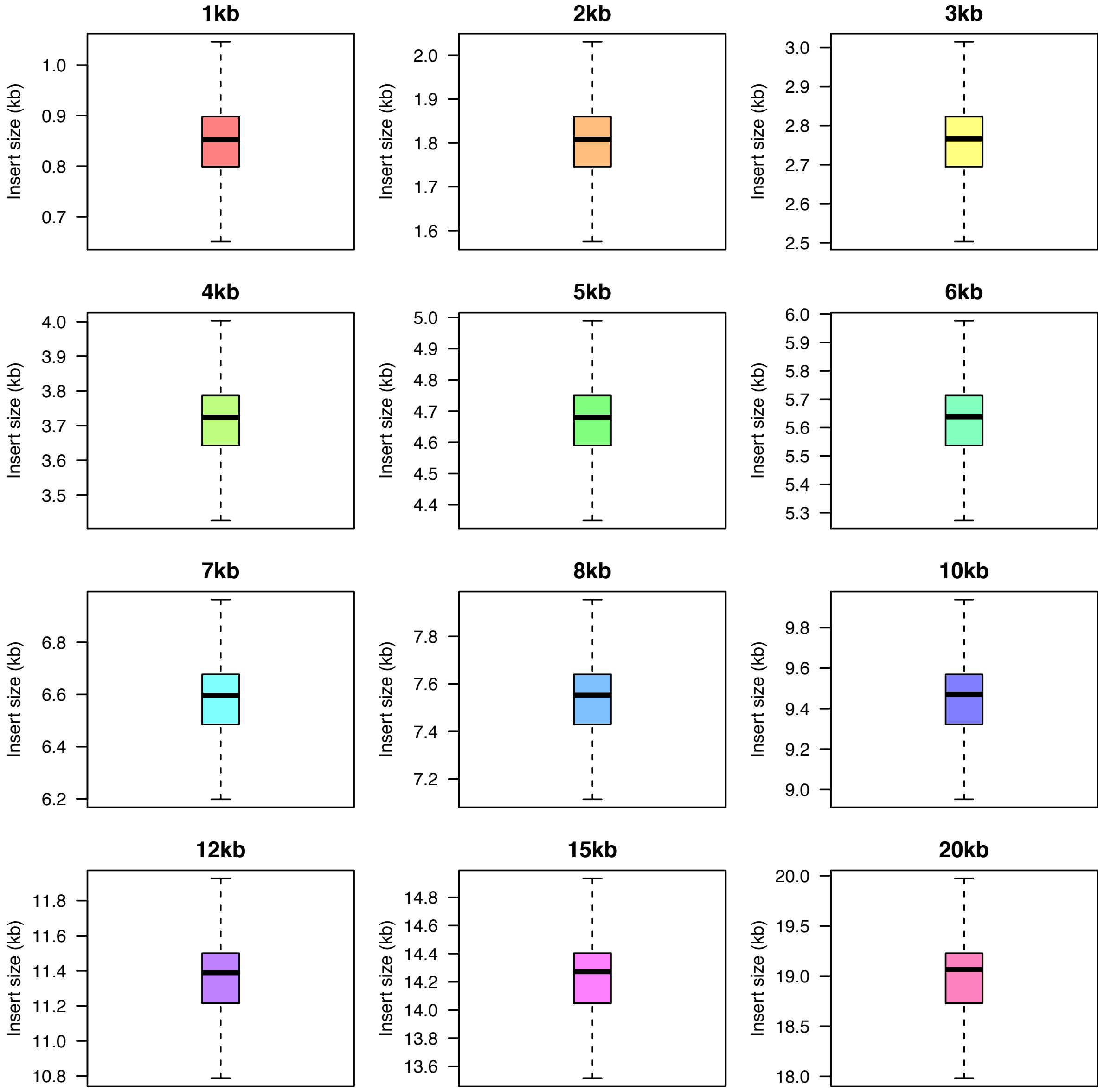

Figure S2: Boxplot of the synthetic libraries extracted by FAST-SG (K22) from the ONT ultra-long reads to scaffold the human (NA12878) genome.

The boxplot was drawn using a total of 900,000 insert sizes from read-pairs mapped within contigs for each synthetic library. The percentage of outliers detected ranged from a minimum of 1.19% and a maximum of 11.56% for 2kb and 180kb respectively.

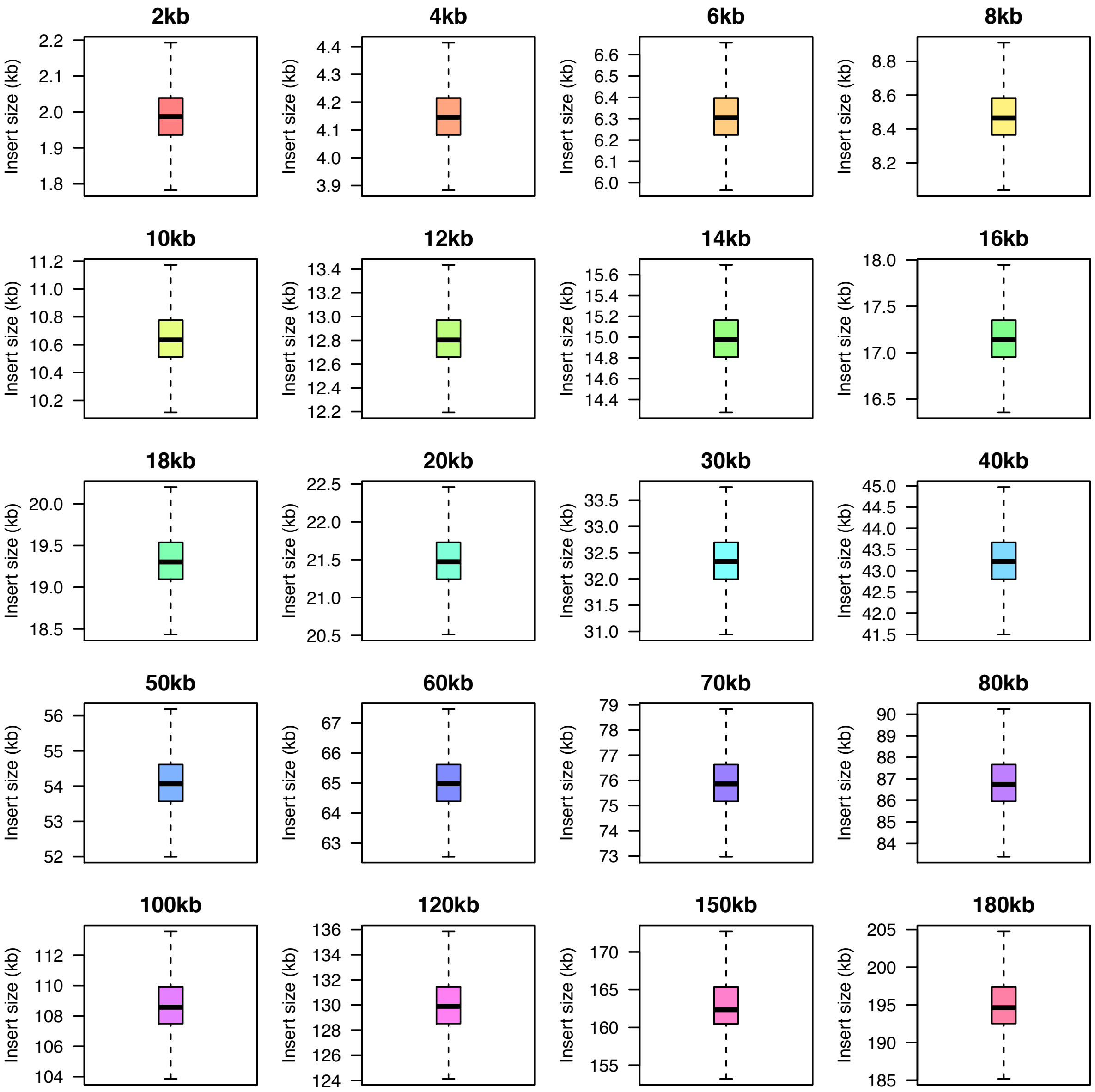

Figure S3: Amount of bases involved in structural errors by type in the *Arabidopsis thaliana* (Ler-0) assemblies.

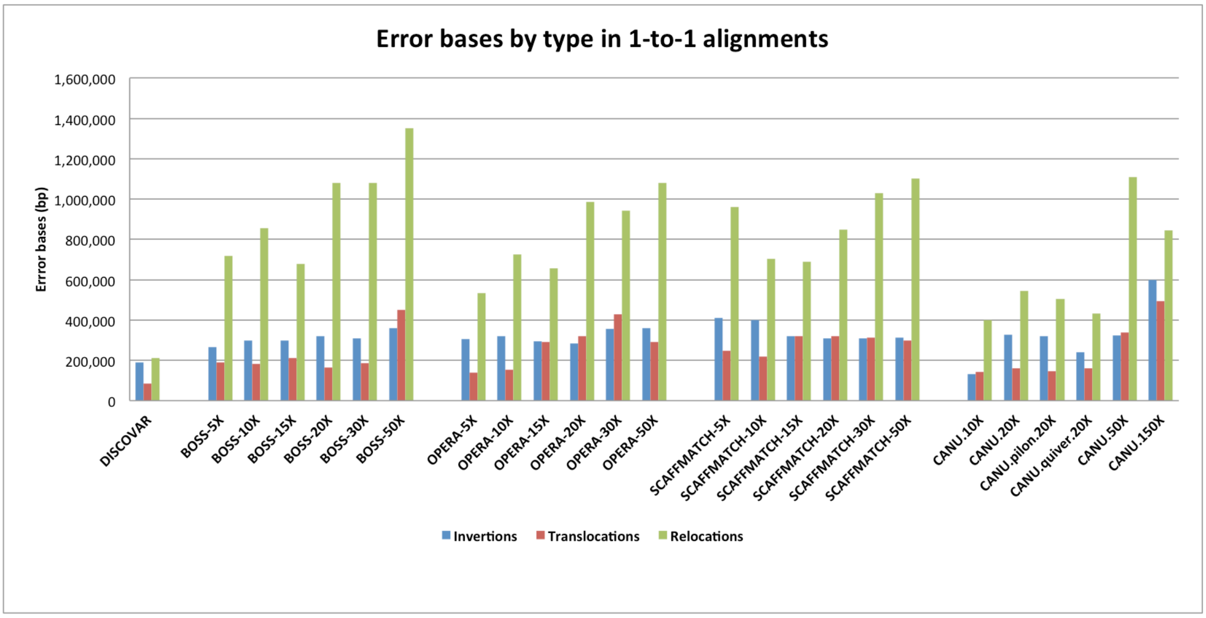

Figure S4: Nucmer plots of the human (NA12878) assemblies. In parenthesis, we show the number of structural errors and the amount of miss-assembled sequences for each assembly.

The plot shows the best (1-to-1) alignments between the reference (*x*-axis) and each assembly (*y*-axis). Red lines indicate forward-strand matches while blue lines indicate reverse-complement matches. The dashed vertical lines delineate the chromosome ends while the dashed horizontal lines delineate the scaffolds. A diagonal indicates concordant matches while off-diagonal matches indicate assembly errors or true DNA differences versus the reference.

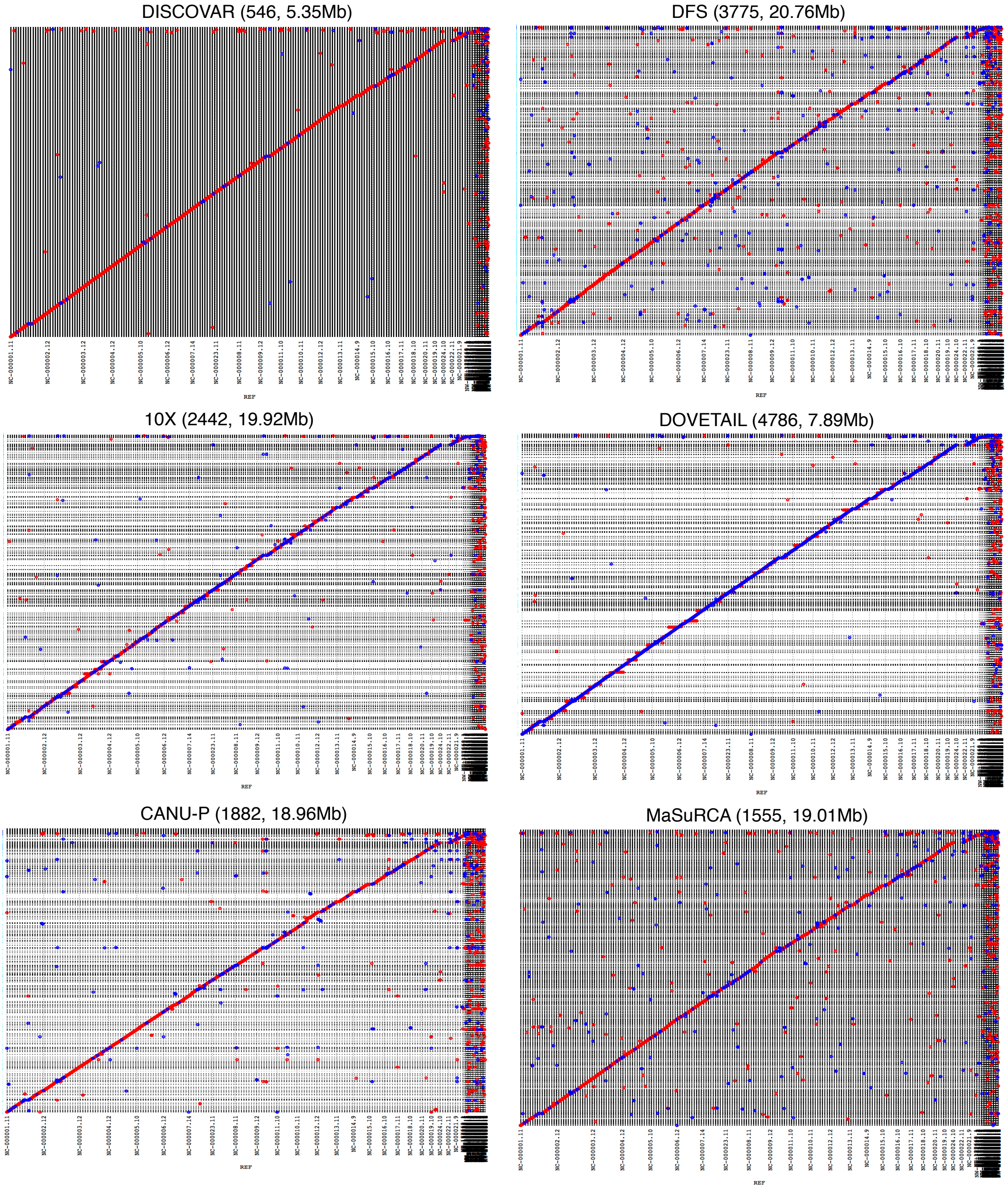

Table S14: Example (blue rows) of short contigs skipped in chromosome 6 by the 10X genomics, DOVETAIL genomics and DISCOVAR+FAST-SG+SCAFFMATCH assembly pipelines.

Start ref	End ref	Start query	End query	Aln. Length	Identity	QLEN	Query coverage	Ref. Name	Query name
**10X Genomics**									
149787377	149859397	44123786	44195724	72021	99.62	61644048	0.12	chr6	4980
149859402	149919865	44195847	44256373	60464	99.65	61644048	0.1	chr6	4980
149919694	149922034	1	2341	2341	100	2341	100	chr6	250327
149922102	149938087	44256774	44272757	15986	99.92	61644048	0.03	chr6	4980
149938329	149942907	4575	1	4579	99.87	4575	100	chr6	262463
149948024	149953425	44369950	44364549	5402	99.96	61644048	0.01	chr6	4980
149956910	149980389	44297758	44321237	23480	99.99	61644048	0.04	chr6	4980
149980662	149983654	1	2994	2993	99.83	2997	99.9	chr6	266826
149984452	150012322	44326238	44354059	27871	98.26	61644048	0.05	chr6	4980
150012599	150015477	1	2878	2879	99.37	2878	100	chr6	271477
150016222	150019555	3334	1	3334	99.76	3334	100	chr6	243667
150019627	150022109	1	2486	2483	99.16	2486	100	chr6	271751
150024639	150168024	44372951	44516259	143386	99.75	61644048	0.23	chr6	4980
150168044	150188479	44516401	44536880	20436	99.69	61644048	0.03	chr6	4980
**DOVETAIL Genomics**									
117924474	117998069	39115310	39041702	73596	99.87	95295052	0.08	chr6	LOQO01006935.1
117998240	118000076	39041601	39039765	1837	99.78	95295052	0	chr6	LOQO01006935.1
117999900	118002040	1	2142	2141	99.72	2142	100	chr6	LOQO01026477.1
118002015	118070052	39039664	38971620	68038	99.83	95295052	0.07	chr6	LOQO01006935.1
118519585	118686144	38522945	38356439	166560	99.77	95295052	0.17	chr6	LOQO01006935.1
118685946	118690872	4930	1	4927	99.37	4930	100	chr6	LOQO01012278.1
118692765	118797230	38356338	38251669	104466	99.61	95295052	0.11	chr6	LOQO01006935.1
119275408	119373298	37773797	37675882	97891	99.75	95295052	0.1	chr6	LOQO01006935.1
119373100	119375749	2649	1	2650	99.74	2649	100	chr6	LOQO01022156.1
119375562	119402267	37675781	37649068	26706	99.89	95295052	0.03	chr6	LOQO01006935.1
119404569	120058896	37648967	36994470	654328	99.82	95295052	0.69	chr6	LOQO01006935.1
120058698	120060702	2005	1	2005	100	2005	100	chr6	LOQO01006524.1
120060504	120155716	36994369	36899183	95213	99.92	95295052	0.1	chr6	LOQO01006935.1
120155733	120205677	36899082	36849173	49945	99.75	95295052	0.05	chr6	LOQO01006935.1
**DISCOVAR+FAST-SG+SCAFFMATCH**									
103293990	103384183	9439402	9349159	90194	99.74	13901799	0.65	chr6	scaf_287
103384151	103492869	9349061	9240351	108719	99.88	13901799	0.78	chr6	scaf_287
103492745	103494866	1	2122	2122	100	2122	100	chr6	scaf_5617
103496319	103557257	9239979	9179120	60939	99.74	13901799	0.44	chr6	scaf_287
103558070	103577774	9178187	9158483	19705	99.83	13901799	0.14	chr6	scaf_287
104246596	104294372	8482783	8435014	47777	99.92	13901799	0.34	chr6	scaf_287
104294467	104376366	8435005	8353119	81900	99.74	13901799	0.59	chr6	scaf_287
104376189	104379769	1	3581	3581	99.41	3581	100	chr6	scaf_1813
104379989	104424335	8349501	8305146	44347	99.57	13901799	0.32	chr6	scaf_287
104424431	104452615	8305146	8276953	28185	99.77	13901799	0.2	chr6	scaf_287

Table S15: Examples (blue rows) of chimeric contigs in chromosome 6 from the CANU and MASURCA asssemblies.

Start ref	End ref	Start query	End query	Aln. Length	Identity	QLEN	Query coverage	Ref. Name	Query name
CANU									
57794754	*57800276*	*607917*	*602445*	*5523*	*96.49*	*6724985*	*0.08*	*chr6*	*tig00001771_pilon_pilon*
57820380	*57865513*	*605097*	*560188*	*45134*	*98.32*	*6724985*	*0.67*	*chr6*	*tig00001771_pilon_pilon*
57865619	*57903199*	*560188*	*522979*	*37581*	*98.36*	*6724985*	*0.55*	*chr6*	*tig00001771_pilon_pilon*
57903360	*57960323*	*522887*	*466997*	*56964*	*97.09*	*6724985*	*0.83*	*chr6*	*tig00001771_pilon_pilon*
58029843	58101842	1	71393	72000	98.88	126739	56.33	chr6	tig00001787_pilon_pilon
58102875	58129124	72249	98311	26250	99.05	126739	20.56	chr6	tig00001787_pilon_pilon
58133200	58161751	98312	126739	28552	99.35	126739	22.43	chr6	tig00001787_pilon_pilon
58178874	*58187991*	*247031*	*237918*	*9118*	*97.91*	*6724985*	*0.14*	*chr6*	*tig00001771_pilon_pilon*
58189266	*58279422*	*237603*	*148546*	*90157*	*97.96*	*6724985*	*1.32*	*chr6*	*tig00001771_pilon_pilon*
58279439	*58380703*	*149330*	*48250*	*101265*	*99.67*	*6724985*	*1.5*	*chr6*	*tig00001771_pilon_pilon*
58380903	*58409019*	*48184*	*20290*	*28117*	*98.91*	*6724985*	*0.41*	*chr6*	*tig00001771_pilon_pilon*
58409262	*58422255*	*20290*	*7823*	*12994*	*93*	*6724985*	*0.19*	*chr6*	*tig00001771_pilon_pilon*
58434762	58453888	5	18179	19127	91.92	27209	66.8	chr6	tig01415240_pilon_pilon
58925651	58931589	7048	12838	5939	96.45	62561	9.26	chr6	tig00006039_pilon_pilon
58981838	*58992692*	*17334*	*27783*	*10855*	*95.52*	*30610*	*34.14*	*chr6*	*tig00007437_pilon_pilon*
59099866	*59106381*	*10806*	*17092*	*6516*	*95.6*	*30610*	*20.54*	*chr6*	*tig00007437_pilon_pilon*
59283293	59289879	29626	23316	6587	95.11	56432	11.18	chr6	tig01415839_pilon_pilon
59784207	*59786687*	*17092*	*19494*	*2481*	*96.09*	*30610*	*7.85*	*chr6*	*tig00007437_pilon_pilon*
59819064	59823095	43966	47876	4032	96.38	62561	6.25	chr6	tig00006039_pilon_pilon
59826711	*59829934*	*1*	*3129*	*3224*	*95.7*	*30610*	*10.22*	*chr6*	*tig00007437_pilon_pilon*
60327010	60357262	418642	388832	30253	98.03	418642	7.12	chr6	tig01415248_pilon_pilon
60357565	60382474	388721	364076	24910	98.38	418642	5.89	chr6	tig01415248_pilon_pilon
60382475	60420111	363884	326369	37637	99.3	418642	8.96	chr6	tig01415248_pilon_pilon
60424420	60481750	326359	269386	57331	98.42	418642	13.61	chr6	tig01415248_pilon_pilon
60513968	60532273	130756	148915	18306	97.89	177522	10.23	chr6	tig00004500_pilon_pilon
60598775	60617561	141784	123356	18787	96.76	141784	13	chr6	tig00004223_pilon_pilon
60617845	60624986	123306	116351	7142	96.67	141784	4.91	chr6	tig00004223_pilon_pilon
60625183	60653795	116343	88040	28613	98.29	141784	19.96	chr6	tig00004223_pilon_pilon
60653802	60692782	88069	49293	38981	98.88	141784	27.35	chr6	tig00004223_pilon_pilon
60693221	60742365	48823	1	49145	99.12	141784	34.43	chr6	tig00004223_pilon_pilon
60712271	*60934236*	*466982*	*246511*	*221966*	*98.64*	*6724985*	*3.28*	*chr6*	*tig00001771_pilon_pilon*
60934237	*60942572*	*245966*	*237623*	*8336*	*98.57*	*6724985*	*0.12*	*chr6*	*tig00001771_pilon_pilon*
MASURCA									
54070031	54088375	2418781	2437126	18346	99.87	4467207	0.41	*chr6*	*scf7180000037033*
54088408	54329253	2437124	2677884	240761	99.79	4467207	5.39	chr6	*scf7180000037033*
54332715	54338649	2681055	2686988	5934	99.92	4467207	0.13	chr6	*scf7180000037033*
54342314	54379187	1	36766	36766	99.44	36766	100	chr6	*scf7180000036086*
54365485	54605150	2713887	2953362	239476	99.74	4467207	5.36	chr6	*scf7180000037033*
54605025	54691651	2953140	3039695	86556	99.67	4467207	1.94	chr6	*scf7180000037033*
54691973	54937730	3039682	3285158	245477	99.61	4467207	5.5	chr6	*scf7180000037033*
55910169	55953093	4256297	4299217	42921	99.93	4467207	0.96	chr6	*scf7180000037033*
55943408	55989719	29671	75927	46257	99.73	75927	60.92	chr6	*scf7180000033017*
55981915	56098441	4307286	4423729	116444	99.72	4467207	2.61	chr6	*scf7180000037033*
56098982	56142129	4424129	4467207	43079	99.64	4467207	0.96	chr6	*scf7180000037033*

Supplementary Material 4: Illumina alignment benchmark.

Simulated Illumina reads

Twenty million pair-end reads of 101bp in length were simulated from the complete human reference genome using WGSIM (version 0.1.19) with 1.5% substitution errors, 0.2% INDELs variants and with fragments size following a normal distribution N(500, 50^2). The WGSIM command used was the following:

```
wgsim -N 20000000 -1 101 -2 101 GRCh38_latest_genomic.fna default.20MB.fwd.fq
default.20MB.rev.fq > 20MB.log
```

Finally, the simulated short reads were compressed using the GZIP utility.

Short read alignments

The following commands were used to align the simulated reads to the human reference genome:

BOWTIE2 global mode:

```
#Index
time bowtie2-build  --threads 1 human.fa human.fa-blocal
#Alignment using 1 CPU
time bowtie2  -p 1 -x human.fa-blocal -1 reads/default.20MB.fwd.fq.gz -2
reads/default.20MB.rev.fq.gz 2> lib20MB.bowtie2.log  > lib20MB.bowtie2.sam
#Alignment using 10 CPU
time bowtie2  -p 10 -x human.fa-blocal -1 reads/default.20MB.fwd.fq.gz -2
reads/default.20MB.rev.fq.gz 2> lib20MB.bowtie2.P10.log  > lib20MB.bowtie2.P10.sam
```

BOWTIE2 local mode

```
#Index
time bowtie2-build  --threads 1 human.fa human.fa-blocal
#Alignment using 1 CPU
time bowtie2 --local -p 1  -x human.fa-blocal -1 reads/default.20MB.fwd.fq.gz -2
reads/default.20MB.rev.fq.gz 2>  lib20MB.bowtie2local.log  > lib20MB.bowtie2local.sam
#Alignment using 10 CPU
time bowtie2 --local -p 10 -x human.fa-blocal -1 reads/default.20MB.fwd.fq.gz -2
reads/default.20MB.rev.fq.gz 2>  lib20MB.bowtie2local.P10.log  > lib20MB.bowtie2local.P10.sam
```

BOWTIE

```
#Index
time bowtie-build human.fa human.fa 2>bowtie.db.err > bowtie.db.log
#Alignment using 1 CPU
time zcat reads/default.20MB.fwd.fq.gz  | bowtie  -p 1 -m 1 -q human.fa - -S lib20MB-
left.bowtie.sam 2> lib20MB-left.bowtie.log
time zcat reads/default.20MB.rev.fq.gz  | bowtie  -p 1 -m 1 -q human.fa - -S lib20MB-
right.bowtie.sam 2> lib20MB-right.bowtie.log
#Alignment using 10 CPU
time zcat reads/default.20MB.fwd.fq.gz  | bowtie  -p 10 -m 1 -q human.fa - -S lib20MB-
left.bowtie.P10.sam 2> lib20MB-left.bowtie.P10.log
time zcat reads/default.20MB.rev.fq.gz  | bowtie  -p 10 -m 1 -q human.fa - -S lib20MB-
right.bowtie.P10.sam 2> lib20MB-right.bowtie.P10.log
```

BWA-MEM

```
#Index
time bwa index human.fa 2> bwa.db.err >bwa.db.log
#Alignment using 1 CPU
time bwa mem -t 1 human.fa  reads/default.20MB.fwd.fq.gz  reads/default.20MB.rev.fq.gz 2>
human.bwamem.log > human.bwamem.sam
#Alignment using 10 CPU
time bwa mem -t 10 human.fa  reads/default.20MB.fwd.fq.gz  reads/default.20MB.rev.fq.gz 2>
human.bwamem.P10.log > human.bwamem.P10.sam
```

BWA

```
#Index
time bwa index human.fa 2> bwa.db.err >bwa.db.log
#Alignment using 1 CPU
```

```
time bwa  aln -t 1 -f lib20MB-left.BWA.sai human.fa reads/default.20MB.fwd.fq.gz 2> lib20MB-
left.BWA.log
time bwa samse -n 1 -f lib20MB-left.BWA.sam human.fa lib20MB-left.BWA.sai
reads/default.20MB.fwd.fq.gz 2>lib20MB-left.BWA.log2
time bwa  aln -t 1 -f lib20MB-right.BWA.sai human.fa reads/default.20MB.rev.fq.gz 2> lib20MB-
rigth.BWA.log
time bwa samse -n 1 -f lib20MB-right.BWA.sam human.fa lib20MB-right.BWA.sai
reads/default.20MB.fwd.fq.gz 2>lib20MB-right.BWA.log2
#Alignment using 10 CPU
time bwa  aln -t 10 -f lib20MB-left.BWA.sai human.fa reads/default.20MB.fwd.fq.gz 2> lib20MB-
left.BWA.log
time bwa samse -n 1 -f lib20MB-left.BWA.T10.sam human.fa lib20MB-left.BWA.sai
reads/default.20MB.fwd.fq.gz 2>lib20MB-left.BWA.log2
time bwa  aln -t 10 -f lib20MB-right.BWA.sai human.fa reads/default.20MB.rev.fq.gz 2>
lib20MB-rigth.BWA.log
time bwa samse -n 1 -f lib20MB-right.BWA.T10.sam human.fa lib20MB-right.BWA.sai
reads/default.20MB.fwd.fq.gz 2>lib20MB-right.BWA.log2
```

FAST-SG

```
#read_file.txt content:
short lib1 reads/default.20MB.fwd.fq.gz reads/default.20MB.rev.fq.gz 1
#Alignment using 1 CPU
time ./FAST-SG.pl -k 15-80:5 -l read_file.txt –r human.fa -p hbenchmark –t 1
#Alignment using 10 CPU
time ./FAST-SG.pl -k 15-80:5 -l read_file.txt –r human.fa -p hbenchmark –t 10
```

The computation time for each short read aligner and FAST-SG was measured using the Linux time utility, in particular we considered the real time value. All benchmarks were executed in the Chilean NLHPC cluster (National Laboratory for High Performance Computing) using machines equipped with Intel(R) Xeon(R) E5-2660 v2 processors (2.20GHz) and 48 Gb of RAM. The exclusive execution of each tool was guaranteed using the –exclusive option in the SLURM jobs. The benchmark statistics (recall, precision, error rate) were computed from alignments with a minimum mapping quality score of 20. An alignment was considered *wrong* if its start position was outside of the simulated fragment position.

Table S16: Short read alignment benchmark.

2 × 10^7 million read pairs of 101bp were simulated from the human reference genome (GRCh38.p10) using WGSIM with 1.5% substitution errors and 0.2% INDELs variants. The insert size follows a normal distribution $N(500, 50^2)$. The benchmark variables (recall and precision) were computed using alignments with a minimum mapping quality of 20. An alignment was considered wrong if its start position was outside of the simulated fragment region. FAST-SG was run using the default Illumina parameters with various k-mer sizes (from k=15 to k=80, increasing by 5 bases). The short read aligners were run using the default settings except for BOWTIE, where we specified to report only the reads having an unique alignment due to the fact that BOWTIE does not compute mapping quality scores. The time used by each short read mapper and by FAST-SG was measured using the Linux time utility. In particular, we considered the real time value in seconds. BOWTIE2-LOCAL and BOWTIE2-GLOBAL are the local and global alignment modes of BOWTIE2 respectively.

	INDEX(s)	Mapping			Reads/sec		Precision (%)	Recall(%)
		1 CPU(s)	10 CPU(s)	Scale factor	1 CPU	10 CPU		
K15	1,952	1,981	260	7.619	20,191	153,846	99.932	2.679
K20	6,362	1,508	240	6.283	26,525	166,666	99.349	62.374
K25	8,359	1,915	249	7.691	20,887	160,642	99.229	71.117
K30	8,810	2,101	264	7.958	19,038	151,515	99.211	69.704
K35	9,187	2,065	259	7.973	19,370	154,440	99.250	64.118
K40	9,507	2,104	255	8.251	19,011	156,862	99.309	55.525
K45	9,677	1,908	244	7.820	20,964	163,934	99.370	45.948
K50	9,996	1,828	233	7.845	21,881	171,673	99.448	35.402
K55	9,672	1,441	213	6.765	27,758	187,793	99.493	26.202
K60	10,209	1,495	198	7.551	26,755	202,020	99.550	19.200
K65	10,445	1,428	196	7.286	28,011	204,081	99.644	13.867
K70	10,778	1,245	183	6.803	32,128	218,579	99.455	10.023
K75	11,249	1,123	180	6.239	35,618	222,222	99.423	7.147
K80	10,992	961	174	5.523	41,623	229,885	99.452	5.036
BOWTIE2_G	6,481	14,341	1,833	7.824	2,789	21,822	99.742	79.838
BOWTIE2_L	6,481	33,783	4,144	8.152	1,184	9,652	98.177	75.666
BOWTIE	7,715	15,970	1,912	8.353	2,504	20,920	99.959	71.493
BWA-MEM	3,661	21,800	2,717	8.024	1,834	14,722	99.977	89.272
BWA-ALN	3,661	20,566	3,985	5.161	1,944	10,037	99.967	83.529

Supplemental Material 5: Illumina scaffolding benchmark.

Short read alignments by scaffolder

The short read alignments were executed using wrappers or following the instructions provided by the scaffolding tools. Otherwise, the short read aligners were run using their default options when possible. The specific commands for each combination of short read aligner and scaffolder used for all short read datasets were the following:

OPERA-LG/BESST2 alignments

OPERA-LG accepts short read alignments in SAM/BAM format and requires read alignments reported in paired or single format. BESST2 supports read aligned in pair-end mode only. OPERA-LG and BESST2 provide wrappers to execute the short read aligner tools. In particular, OPERA-LG provides preprocess_reads.pl for BOWTIE and BWA. BESST2 provides reads_to_ctg_map.py for BWA and BWA-MEM. BOWTIE was not used with BESST2 because it cannot report alignments in pair-end mode for pairs connecting contigs. SAMTOOLS was used for the SAM/BAM file conversions. The commands were the followings:

BOWTIE2_GLOBAL

```
bowtie2  -x ${CTG}.fa -1 ${FWD} -2 ${REV} 2>${ORG}.bowtie2.log | samtools view -Sb - >
${ORG}.bowtie2.bam
```

BOWTIE2_LOCAL

```
bowtie2 --local -x ${CTG}.fa -1 ${FWD} -2 ${REV} 2>${ORG}.bowtie2local.log | samtools view -
Sb - > ${ORG}.bowtie2local.bam
```

BOWTIE

```
#OPERA-LG
preprocess_reads.pl  --contig ${CTG}.fa --illumina-read1 ${FWD} --illumina-read2 ${REV} --out
${ORG}.bowtie.sam  --map-tool bowtie
```

BWA-MEM

```
#OPERA-LG
bwa  mem  ${CTG}.fa  ${FWD}  ${REV}  2>  ${ORG}.bwamem.log  |  samtools  view  -Sb  -  >
${ORG}.bwamem.bam
```

BWA

```
#OPERA-LG
preprocess_reads.pl  --contig ${CTG}.fa --illumina-read1 ${FWD} --illumina-read2 ${REV} --out
${ORG}.bwa.bam
#BESST2
reads_to_ctg_map.py  ${FWD} ${REV} ${CTG}.fa  ${LIB}-bwa-besst --threads 1 --nomem > ${LIB}-
besst-bwa-log.txt
```

FAST-SG

```
#read_file.txt content:
short lib1 ${FWD} ${REV} 2
#Alignment using 1 CPU
./FAST-SG.pl -k ${K} -l read_file.txt -r ${CTG}.fa -p ${OUT}
```

SCAFFMATCH/BOSS alignments

SCAFFMATCH and BOSS require the alignments of the short read in single-end format. SAMTOOLS was used for the SAM/BAM conversions because BOSS processes directly the alignments in BAM format. The commands used were the following:

BOWTIE2_GLOBAL

```
bowtie2  --quiet --no-hd --reorder -q -x ${CTG}.fa -U ${FWD} -S ${LIB}-left.bowtie2.sam 2>
${LIB}-left.bowtie2.log
bowtie2  --quiet --no-hd --reorder -q -x ${CTG}.fa -U ${REV} -S ${LIB}-right.bowtie2.sam 2>
${LIB}-right.bowtie2.log
```

BOWTIE2_LOCAL

```
bowtie2 --local --quiet --no-hd --reorder  -q -x ${CTG}.fa -U ${FWD} -S ${LIB}-
left.bowtie2local.sam 2> ${LIB}-left.bowtie2local.log
bowtie2 --local --quiet --no-hd --reorder  -q -x ${CTG}.fa -U ${REV} -S ${LIB}-
right.bowtie2local.sam 2> ${LIB}-right.bowtie2local.log
```

BOWTIE

```
zcat ${FWD} | bowtie --quiet --sam-nohead -v 3 -q ${CTG}.fa - -S ${LIB}-left.bowtie.sam 2>
${LIB}-left.bowtie.log
zcat ${REV} | bowtie --quiet --sam-nohead -v 3 -q ${CTG}.fa - -S ${LIB}-right.bowtie.sam 2>
${LIB}-right.bowtie.log
```

BWA-MEM

```
bwa mem ${CTG}.fa ${FWD} 2> ${LIB}-left.bwa.log  > ${LIB}-left.bwa.sam
bwa mem ${CTG}.fa ${REV} 2> ${LIB}-right.bwa.log > ${LIB}-right.bwa.sam
```

BWA

```
bwa aln -f ${LIB}-left.BWA.sai ${CTG}.fa ${FWD} 2>${LIB}-left.BWA.log
bwa samse -n 1 -f ${LIB}-left.BWA.sam ${CTG}.fa ${LIB}-left.BWA.sai ${FWD} 2>${LIB}-
left.BWA.log2
bwa aln -f ${LIB}-right.BWA.sai ${CTG}.fa ${REV} 2>${LIB}-right.BWA.log
bwa samse -n 1 -f ${LIB}-right.BWA.sam ${CTG}.fa ${LIB}-right.BWA.sai ${REV} 2>${LIB}-
right.BWA.log2
```

FAST-SG

```
#read_file.txt content:
short lib1 ${FWD} ${REV} 1
#Alignment using 1 CPU
./FAST-SG.pl -k ${K} -l read_file.txt -r ${CTG}.fa -p ${OUT}
```

Scaffolder settings by dataset

To perform a fair comparison between the short read aligners and FAST-SG, all scaffolding tools were run with identical parameters on each short read alignment result. Specifically, the scaffolder settings used were as follows:

OPERA-LG settings

OPERA-LG computes the average insert-size, standard deviation and orientation of the mate-pair library from the alignments. We used the following OPERA-LG configuration file for all datasets.

All datasets

```
OPERA-${MAPPER}.conf:
output_folder=opera-results-${MAPPER}
contig_file=${DATASET}.contigs.fa
kmer=39
filter_repeat=yes
[LIB]
map_file=${MAPPER}.bam
#OPERA-LG run Command
OPERA-LG OPERA-${MAPPER}.conf
Where MAPPER is replaced by each short-read aligner or FAST-SG
```

SCAFFMATCH settings

SCAFFMATCH requires as parameters the average insert size, standard deviation and orientations for each mate-pair library. The commands were the followings:

S. aureus mate-pair library

```
scaffmatch  -m -w ${LIB}-SM-${MAPPER} -c ${CTG}.fa -1  ${LIB}-left.${MAPPER}.sam -2 ${LIB}-right.${MAPPER}.sam -i 3600 -p rf -s 500
```

R. sphaeroides mate-pair library

```
scaffmatch  -m -w ${LIB}-SM-${MAPPER} -c ${CTG}.fa -1  ${LIB}-left.${MAPPER}.sam -2 ${LIB}-right.${MAPPER}.sam -i 3700 -p rf -s 500
```

P. falciparum pair-end library

```
scaffmatch  -m -w ${LIB}-SM-${MAPPER} -c ${CTG}.fa -1  ${LIB}-left.${MAPPER}.sam -2 ${LIB}-right.${MAPPER}.sam -i 650 -p fr -s 100
```

P. falciparum mate-pair library

```
scaffmatch  -m -w ${LIB}-SM-${MAPPER} -c ${CTG}.fa -1  ${LIB}-left.${MAPPER}.sam -2 ${LIB}-right.${MAPPER}.sam -i 2700 -p rf -s 500
```

H. sapiens mate-pair library

```
scaffmatch  -m -w ${LIB}-SM-${MAPPER} -c ${CTG}.fa -1  ${LIB}-left.${MAPPER}.sam -2 ${LIB}-right.${MAPPER}.sam -i 2500 -p rf -s 340
```

BESST2 settings

BESST2 computes the insert size, standard deviation and orientation for each library from the alignments. The commands were the followings:

S. aureus mate-pair library

```
runBESST -c ${IN}.fa -f lib1.${MAPPER}.bam --orientation rf -o L1FK${MAPPER}-BESST
```

R. sphaeroides mate-pair library

```
runBESST -c ${IN}.fa -f lib1.${MAPPER}.bam --orientation rf -o L1FK${MAPPER}-BESST
```

P. falciparum pair-end library

```
runBESST -c ${IN}.fa -f lib1.${MAPPER}.bam --orientation fr -o L1FK${MAPPER}-BESST
```

P. falciparum mate-pair library

```
runBESST -c ${IN}.fa -f lib1.${MAPPER}.bam --orientation rf -o L2FK${MAPPER}-BESST
```

H. sapiens mate-pair library

```
runBESST -c ${IN}.fa -f lib1.${MAPPER}.bam --orientation rf -o L1FK${MAPPER}-BESST
```

BOSS settings

BOSS requires as parameters the average insert size, standard deviation, pair orientations and read lengths for each library. The commands were the followings:

S. aureus mate-pair library

```
boss ${IN}.fa K2BOSS-${MAPPER}.fwd.bam K2BOSS-${MAPPER}.rev.bam 37 3500 0.07 0.2 5 0 0 boss-${MAPPER}
```

R. sphaeroides mate-pair library

```
boss ${IN}.fa K2BOSS-${MAPPER}.fwd.bam K2BOSS-${MAPPER}.rev.bam 101 3700 0.07 0.2 5 0 0 boss-${MAPPER}
```

P. falciparum pair-end library

```
boss ${IN}.fa K2BOSS-${MAPPER}.fwd.bam K2BOSS-${MAPPER}.rev.bam 76 650 0.07 0.2 5 1 0 boss-${MAPPER}
```

P. falciparum mate-pair library

```
boss ${IN}.fa K2BOSS-${MAPPER}.fwd.bam K2BOSS-${MAPPER}.rev.bam 75 2700 0.07 0.2 5 0 0 boss-${MAPPER}
```

H. sapiens mate-pair library

```
boss ${IN}.fa K2BOSS-${MAPPER}.fwd.bam K2BOSS-${MAPPER}.rev.bam 101  2900 0.07 0.2 5 0 0 boss-${MAPPER}
```

Validation of the scaffolding results

Identical commands were used to validate the scaffolding results obtained by each combination of aligner-scaffolder for each dataset. The commands were the followings:

S. aureus mate-pair library

```
scaff_test_check_using_tags.py --all_circular 3600 artificial_contigs.tag ${SCAFFOLDER}-${MAPPER}-scaffolds.fna reference.fa.fai ${SCAFFOLDER}-${MAPPER}_check
```

R. sphaeroides mate-pair library

```
scaff_test_check_using_tags.py -c CP000143 -c CP000144 -c CP000145 -c CP000146 -c CP000147 3600 artificial_contigs.tag ${SCAFFOLDER}-${MAPPER}-scaffolds.fna reference.fa.fai ${SCAFFOLDER}-${MAPPER}_check
```

P. falciparum pair-end library

```
scaff_test_check_using_tags.py -c Pf_M76611 650 artificial_contigs.tag ${SCAFFOLDER}-${MAPPER}-scaffolds.fna reference.fa.fai ${SCAFFOLDER}-${MAPPER}_check
```

P. falciparum mate-pair library

```
scaff_test_check_using_tags.py -c Pf_M76611 2700 artificial_contigs.tag ${SCAFFOLDER}-${MAPPER}-scaffolds.fna reference.fa.fai ${SCAFFOLDER}-${MAPPER}_check
```

H. sapiens mate-pair library

```
scaff_test_check_using_tags.py 2600 artificial_contigs.tag ${SCAFFOLDER}-${MAPPER}-scaffolds.fna reference.fa.fai ${SCAFFOLDER}-${MAPPER}_check
```

Figure S5: Percentage of pair-end reads aligned by FAST-SG (K) and the short read aligners for each Illumina dataset. The horizontal line is the average percentage of mate-pairs aligned by FAST-SG considering all *k*-mer sizes.

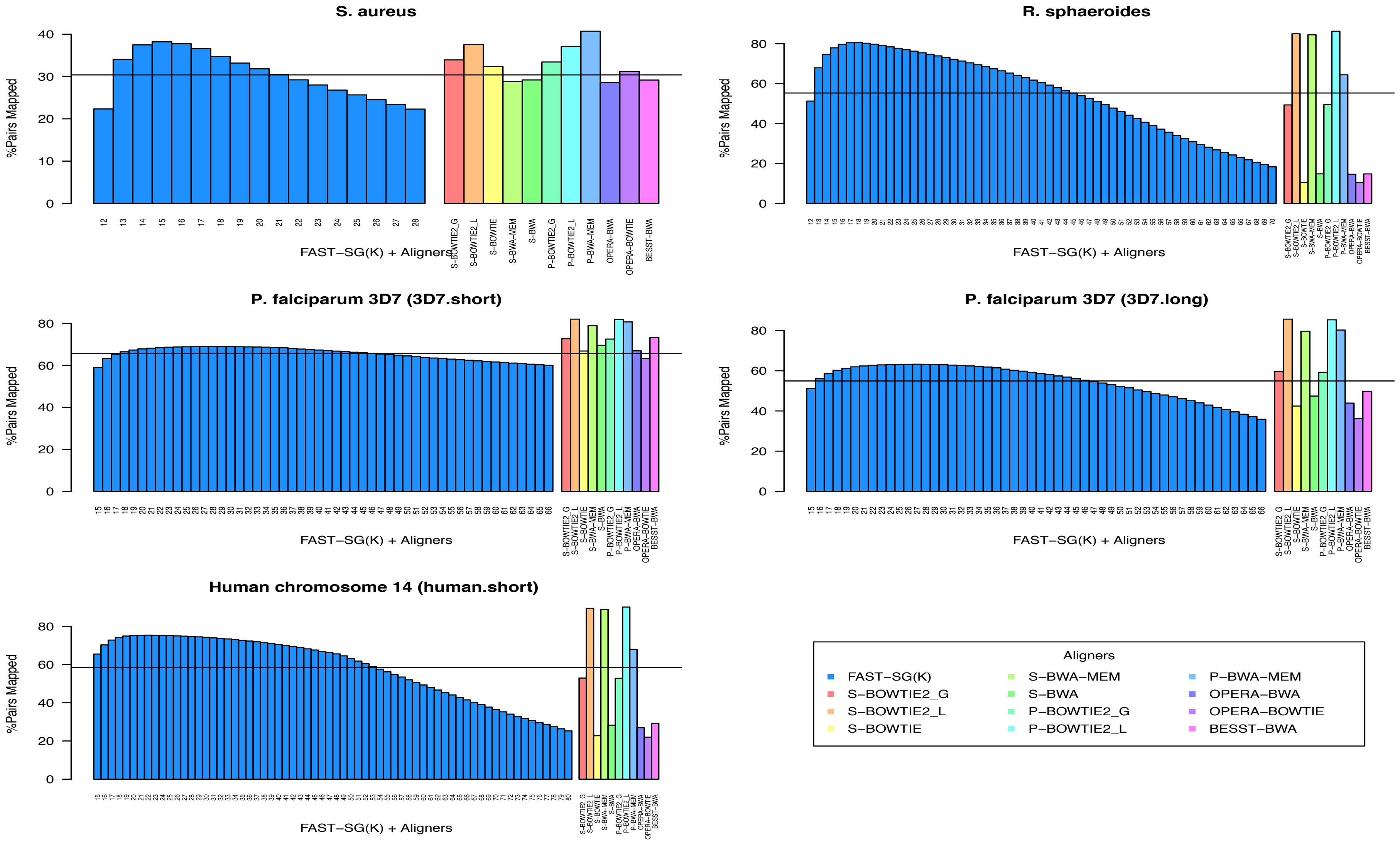

Figure S6: Pairwise contig read coverage correlation between the short read aligners and FAST-SG.

The average contig read coverage was extracted from OPERA-LG (file contigs in the OPERA-LG results) for each aligner; the contig read coverage was normalized dividing each value for the total contig read coverage (addition of each column). The pairwise correlation among the aligners was computed using the Pearson method and was plotted using the corrplot library from R. The blue numbers correspond to the pairwise correlations among aligners. In parenthesis, we show the number of contigs for each dataset. The *K*-values correspond to FAST-SG.

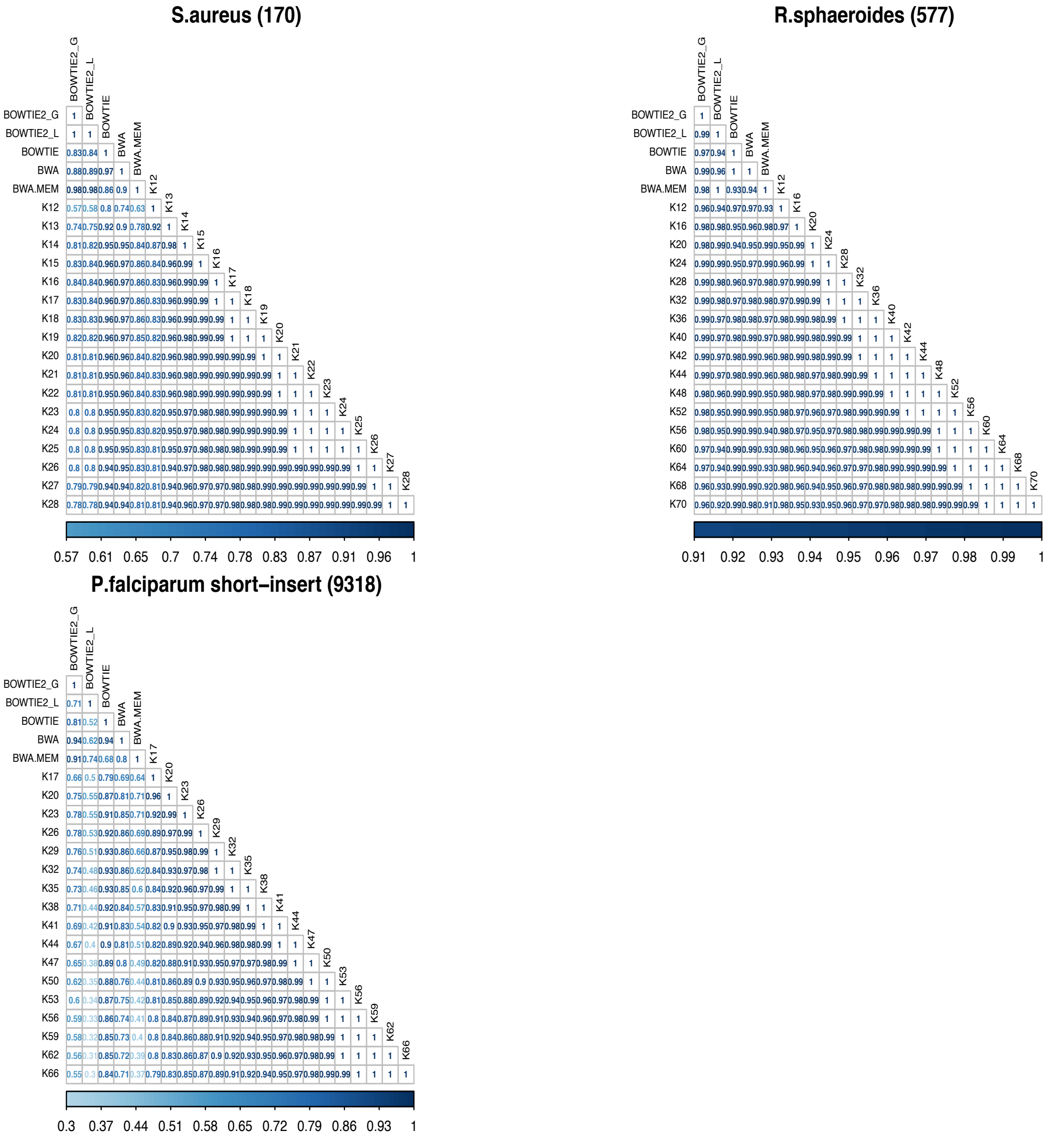

Scaffolding benchmark results for *S. aureus*

The Supplementary_Table_S17.xlsx contains the benchmark details for *S. aureus* using the SCAFFMATCH, OPERA-LG, BESST2 and BOSS scaffolders. The Illumina reads were aligned with the short read aligners and FAST-SG. FAST-SG was run using various *k*-mer size values (*K*=12-28).

Scaffolding benchmark results for *R. sphaeroides*

The Supplementary_Table_S18.xlsx contains the benchmark details for *R. sphaeroides* using the SCAFFMATCH, OPERA-LG, BESST2 and BOSS scaffolders. The Illumina reads were aligned with the short read aligners and FAST-SG. FAST-SG was run using various *k*-mer size values (*K*=12-70).

Scaffolding benchmark results for *P. falciparum*

The Supplementary_Table_S19.xlsx contains the benchmark details for *P. falciparum* using the SCAFFMATCH, OPERA-LG, BESST2 and BOSS scaffolders. The Illumina reads were aligned with the short read aligners and FAST-SG. FAST-SG was run using various *k*-mer size values (*K*=15-66).

Scaffolding benchmark results for *H. sapiens*

The Supplementary_Table_S20.xlsx contains the benchmark details for *H. sapiens* using the SCAFFMATCH, OPERA-LG, BESST2 and BOSS scaffolders. The Illumina reads were aligned with the short read aligners and FAST-SG. FAST-SG was run using various *k*-mer size values (*K*=15-80).

References

Gao S, Bertrand D, Chia BKH, Nagarajan N. 2016. OPERA-LG: Efficient and exact scaffolding of large, repeat-rich eukaryotic genomes with performance guarantees. *Genome Biol* **17**: 102.

Hunt M, Newbold C, Berriman M, Otto TD. 2014. A comprehensive evaluation of assembly scaffolding tools. *Genome Biol* **15**: R42.

Jain M, Koren S, Quick J, Rand AC, Sasani TA, Tyson JR, Beggs AD, Dilthey AT, Fiddes IT, Malla S, et al. 2017. Nanopore sequencing and assembly of a human genome with ultra-long reads. *bioRxiv* 128835.

Kokot M, Długosz M, Deorowicz S. KMC 3: counting and manipulating *k*-mer statistics. *Bioinformatics*.

Koren S, Walenz BP, Berlin K, Miller JR, Bergman NH, Phillippy AM. 2017. CANU: Scalable and accurate long-read assembly via adaptive *k*-mer weighting and repeat separation. *Genome Res* **27**: 722–736.

Kurtz S, Phillippy A, Delcher AL, Smoot M, Shumway M, Antonescu C, Salzberg SL. 2004. Versatile and open software for comparing large genomes. *Genome Biol* **5**: R12.

Langmead B, Salzberg SL. 2012. Fast gapped-read alignment with BOWTIE2. *Nat Methods* **9**: 357–359.

Langmead B, Trapnell C, Pop M, Salzberg SL. 2009. Ultrafast and memory-efficient alignment of short DNA sequences to the human genome. *Genome Biol* **10**: R25.

Li H. 2013. Aligning sequence reads, clone sequences and assembly contigs with BWA-MEM. *ArXiv13033997 Q-Bio*.

Li H, Durbin R. 2009. Fast and accurate short read alignment with Burrows–Wheeler transform. *Bioinformatics* **25**: 1754–1760.

Li H, Handsaker B, Wysoker A, Fennell T, Ruan J, Homer N, Marth G, Abecasis G, Durbin R, 1000 Genome Project Data Processing Subgroup. 2009. The Sequence Alignment/Map format and SAMTOOLS. *Bioinforma Oxf Engl* **25**: 2078–2079.

Luo J, Wang J, Zhang Z, Li M, Wu F-X. 2017. BOSS: A novel scaffolding algorithm based on an optimized scaffold graph. *Bioinforma Oxf Engl* **33**: 169–176.

Mandric I, Zelikovsky A. 2015. SCAFFMATCH: Scaffolding algorithm based on maximum weight matching. *Bioinforma Oxf Engl* **31**: 2632–2638.

Phillippy AM, Schatz MC, Pop M. 2008. Genome assembly forensics: Finding the elusive mis-assembly. *Genome Biol* **9**: R55.

Putnam NH, O'Connell BL, Stites JC, Rice BJ, Blanchette M, Calef R, Troll CJ, Fields A, Hartley PD, Sugnet CW, et al. 2016. Chromosome-scale shotgun assembly using an *in vitro* method for long-range linkage. *Genome Res*.

Sahlin K, Chikhi R, Arvestad L. 2016. Assembly scaffolding with PE-contaminated mate-pair libraries. *Bioinforma Oxf Engl* **32**: 1925–1932.

Salmela L, Rivals E. 2014. LORDEC: Accurate and efficient long read error correction. *Bioinformatics* **30**: 3506–3514.

Warren RL, Yang C, Vandervalk BP, Behsaz B, Lagman A, Jones SJM, Birol I. 2015. LINKS: Scalable, alignment-free scaffolding of draft genomes with long reads. *GigaScience* **4**: 35.

Weisenfeld NI, Kumar V, Shah P, Church DM, Jaffe DB. 2017. Direct determination of diploid genome sequences. *Genome Res*.

Weisenfeld NI, Yin S, Sharpe T, Lau B, Hegarty R, Holmes L, Sogoloff B, Tabbaa D, Williams L, Russ C, et al. 2014. Comprehensive variation discovery in single human genomes. *Nat Genet* **46**: 1350–1355.

Zimin AV, Puiu D, Luo M-C, Zhu T, Koren S, Marcais G, Yorke JA, Dvorak J, Salzberg SL. 2017. Hybrid assembly of the large and highly repetitive genome of *Aegilops tauschii*, a progenitor of bread wheat, with the mega-reads algorithm. *Genome Res* gr.213405.116.
