# Supplementary material for: Fast-SG: an alignment-free algorithm for hybrid assembly
Source: Gigascience. 2018 May 5;7(5):giy048. doi: 10.1093/gigascience/giy048 (PMC6007556; doi:10.1093/gigascience/giy048)
Supplement: Response-to-Reviewer-Comments_Original-Submission.pdf [file giy048_response-to-reviewer-comments_original-submission.pdf]

Author's Response To Reviewer Comments

Dear Dr. Edmunds,

We are resubmitting a revised and improved manuscript of “FAST-SG: An alignment-free algorithm for hybrid assembly”. We have carefully considered all the points raised by the reviewers and we believe that we addressed all of them.
Our point-by-point answers to the editor’s and to the reviewers’ comments are the followings:

Point raised by the Editor:

In addition, please register any new software application in the SciCrunch.org database to receive a RRID (Research Resource Identification Initiative ID) number, and include this in your manuscript. This will facilitate tracking, reproducibility and re-use of your tool.

A: We registered our software in SciCrunch.org and our RRID is SCR_015934 (https://scicrunch.org/browse/resources/SCR_015934). We included this information in the “Availability and requirements” section.

Points raised by Reviewer #1:

Reviewer #1: Manuscript Summary (Sagar Utturkar):
Author's present a novel algorithm FAST-SG that can generate scaffolding graphs even with low (~5X) long-read coverage and produce synthetic libraries with long insert sizes. These can be combined using multiple legacy scaffolding tools and generate assembly results comparable to long-read assemblers (requiring high (~30x) coverage).

Review Summary:
The FAST-SG algorithm and results presented here are novel and valid. This algorithm would serve as a nice addition to current tools for generating high-quality assemblies with low long-read coverage data. However, I recommend a major revision in terms of structure and organization to make it more appealing to the readers. Also request authors to include some additional information.

Review comments:
As per my understanding, the main features of the FAST-SG are:
* Generating scaffolding graphs and synthetic mate-pair libraries which have library size up to BACs (180kb).
* This could be achieved with very low coverage of long-reads (5X) and provides a novel paradigm for hybrid assembly
* These FAST-SG libraries can be combined with any leading scaffolders to generate high-quality assemblies
* FAST-SG assembly results (with 5X long-read coverage) are as good as long-reads heavy (50x) assembly
* FAST-SG is compatible with bacterial as well as human genome and can be achieved with moderate computational power.

These are very interesting results. However, currently manuscript present these results in a somewhat imperative manner rather than stating those upfront. From the last paragraph in the background section and overall results section, this appears as a benchmarking study and presented in kind of report format. Author's should consider stating the novelty and important findings upfront, shorten the section on comparison with read-aligners and include a section on how to effectively use this algorithm (See below).

A: We followed the reviewer’s suggestion and we reorganized entirely the Results section as well as the end of the Introduction to bring forward and better present the novelty and more important findings of our work. Moreover, we shortened the section on Illumina by merging the two previous sections (“Comparison of FAST-SG with the state-of-the-art short read aligners” and “Comparison of FAST-SG with the short read aligners commonly used for constructing a scaffolding graph”) into a new one called “Compatibility of FAST-SG with Illumina mate-pair libraries”. Additionally, we included a new section called “Procedure for effective hybrid assembly with FAST-SG” as suggested.

I see that FAST-SG is used in conjunction with other tools (DISCOVARDENOVO, LORDEC, SCAFFMATCH, BOSS, BESST2, OPERA-LG). It would be beneficial if authors could comment on:

* How to choose one scaffolder over other or any recommendations based on available data types/sequence coverage. Or it is suggested to try out multiple scaffolders and choose the best assembly by statistics?

A: In the scaffolding benchmarks, we show that there are two classes of scaffolding tools, one more conservative (OPERA-LG and BESST) and a second one that is more greedy (BOSS and SCAFFMATCH). The more greedy scaffolders reach higher F-score values than the conservative ones (Figure 3-4). However, the greedy ones tend to produce more scaffolding errors (Figure 3-4).
In real hybrid assembly projects, the quality of the assembly depends on the contig and scaffolding steps. According to our evaluations in the Arabidopsis and Human hybrid assemblies, we recommend a more greedy scaffolder (SCAFFMATCH) when the Illumina input assembly is not fragmented (N50> 100Kb). Otherwise, a more conservative scaffolder (Opera-LG) should be used to avoid scaffolding errors.
The above points were included in the new section “Procedure for effective hybrid assembly with FAST-SG”.

* Are these synthetic libraries compatible with any scaffolding tools e.g. SSPACE?

A: In principle, all short-read scaffolders supporting the SAM/BAM input format are compatible with the synthetic mate-pair libraries produced by FAST-SG. We tested four short-read scaffolders (OPERA-LG, BESST2, BOSS and SCAFFMATCH) and we were able to use all of them with Illumina mate pair libraries. However, BESST2 crashed with synthetic mate pair

libraries while it computes the average contig coverage (division by zero) and we thus excluded BESST2 from the hybrid assembly experiments. As for SSPACE, it is currently not compatible with FAST-SG because it does not support the SAM/BAM input and it performs its own alignment step with bowtie.

* How to verify the quality of the generated synthetic libraries?

A: To verify the quality of the synthetic libraries generated, it is possible to plot the observed insert size statistics computed from the read pairs mapped within contigs as we show in the main Figure 2 and the Supplementary Figures S1 and S2. Additionally, statistics on the percentage of outliers and standard deviation can be computed from the observed insert sizes. A too high percentage of outliers (>30%) or a larger than expected standard deviation (>30% of average) are both indicatives of low quality synthetic libraries. The latter can be obtained if the long reads are chimeric (PCR amplification) or if the Illumina assembly is poorly assembled. FAST-SG computes and reports (log file) the observed average insert size for each synthetic library. The observed average insert size allows an easy identification of potentially problematic synthetic libraries. For instance, if the user specified a synthetic library insert size of 8kb and the observed average insert size computed by FAST-SG is 3kb, this would be a strong evidence of a low quality synthetic library and it should not be used in the scaffolding step.
The points above were included in the new section "Procedure for effective hybrid assembly with FAST-SG".

* Consider providing a flowchart for the FAST-SG workflow. Majority of the times, the audience of such manuscripts are biologists who want to use this tool with their data. Author's comments on usability of this tool would be very helpful to make informed decisions.

A: As suggested by the reviewer, we included a section called "Procedure for effective hybrid assembly with FAST-SG", where we provide suggestions based on our experiments / experience on how to take advantage of the FAST-SG hybrid assembly approach. Additionally, we included the recommended workflow (Figure 5) and provided an informative wiki-page describing step-by-step a full hybrid assembly using FAST-SG (https://github.com/adigenova/fast-sg/wiki/Hybrid-scaffolding-of-NA12878).

While comparing the scaffolded assemblies, what is the percentage of N's (uncalled bases) in each assembly? Better continuity may not always a good result if it has a very high percentage of uncalled bases. This will provide an additional estimate of how assembly compares with the 50x coverage data.

A: For the full hybrid assemblies that we performed for Arabidopsis thaliana (Ler-0), the percentage of N's ranged from a minimum of 0.44% (530Kb, BOSS-5X) to a maximum of 1.21% (1.46Mb, ScaffMatch-30X). In the human hybrid assembly, the percentage of N's was 3.15%. Additionally, it is possible to further reduce the percentage of N's by performing a gap-filling step using the short read data but that is out of the scope of our manuscript and will be addressed in a future work as indicated in the Discussion section.

I appreciate the future directions provided in the conclusion section. Consider moving this to the "Results/Discussion" section. Conclusions should be summarizing the usability and novelty of this method.

A: We followed the reviewer’s suggestion and we moved the future directions to Results/Discussion.

Minor comments:

Figure 2. Consider using the thin vertical lines to show the average performance.

A: Done. Now Figure 4.

Figure 4. In the pie charts, provide the number of errors than percentages. This will allow more meaningful comparison.

A: Done. Now Figure 3.

Authors have mentioned 15% error rate for the PacBio data. This should be accompanied by the fact that "this high error rate could be overcome by providing sufficient sequence coverage (100x)".

A: We mentioned in the background section:
“However, de novo assemblies of large genomes based on computing overlaps [5] are computationally intense [4] and require a considerable amount of coverage (50X) to error-correct the inaccurate long read sequences by self-correction methods.”
That covers the point rightfully raised by the reviewer.

Manuscript contains several complex sentences and incorrect use of grammar (e.g. have been instead of has been) and few typos (MySeq should be MiSeq) etc. Please review the manuscript in this aspect. Use of free tools like "Grammarly" may be helpful for rapid revision.

A: Done.

To summarize, this is a very nice and novel work. Above-mentioned changes should help to make it more appealing to the wide audience.

A: We do thank the reviewer for this positive overview of our work.

Reviewer #2 points:

Reviewer #2 (Daniela Puiu): I have read the article and I found it well written and informative, especially for the genome assembly community. While the idea of generating synthetic mate-pairs is not now, you implemented it in the new context of long reads and unique kmers.
I have also managed to download, install and run FAST-SG, and reproduce some of results

described in the article.
However I had difficulties installing and running ScaffMatch, Integrating it in your package and creating a seamless interface between the two programs would be highly recommended.

A: Actually, FAST-SG can work with ScaffMatch but also with several short-read scaffolders (BOSS, OPERA-LG). In order not to be unfair, we preferred not to directly include any in FAST-SG (which in a future work, will contain its own scaffolder, but instead we added a bash script to the source code of FAST-SG that in the case of ScaffMatch, should facilitate its installation (misc/install_scaffMatch.sh).

A: We do thank the reviewer for this positive overview of our work.
