# Supplementary material for: Fast-SG: an alignment-free algorithm for hybrid assembly
Source: Gigascience. 2018 May 5;7(5):giy048. doi: 10.1093/gigascience/giy048 (PMC6007556; doi:10.1093/gigascience/giy048)
Supplement: Response-to-Reviewer-Comments_Revision-1.pdf [file giy048_response-to-reviewer-comments_revision-1.pdf]

Author's Response To Reviewer Comments

GIGA-D-17-00335R1
Fast-SG: An alignment-free algorithm for hybrid assembly
Alex Di Genova, Ph.D; Gonzalo A Ruz, Ph.D; Marie-France Sagot, Ph.D; Alejandro Maass, Ph.D
GigaScience

Dear Dr. Edmunds,

We are resubmitting a revised manuscript of “FAST-SG: An alignment-free algorithm for hybrid assembly”. We have carefully considered all the minor points raised by the reviewers and editors.
Our point-by-point answers to the editor’s and to the reviewers’ minor points are the following:

Point raised by the Editor:

Your manuscript "Fast-SG: An alignment-free algorithm for hybrid assembly" (GIGA-D-17-00335R1) has been assessed by our reviewers. Based on these reports, and my own assessment as Editor, I am pleased to inform you that it is potentially acceptable for publication in GigaScience, once you have carried out some very minor revisions suggested by our reviewers and have also created a snapshot of the code and any test data in our GigaDB repository that you will need to cite in an "Availability of supporting data" section. I've cc'd our curators here and they can help you put this together.

A: We have followed the Editor’s suggestion and we deposited a snapshot of the Fast-SG code as well as all the datasets and results described within the manuscript in the GigaDB repository. Currently, the GigaDB page describing this information is the following:

http://gigadb.org/dataset/view/id/100437/token/hyKuNQtlVs0v5R2D

The GigaDB curators on 16/04/2018 should finish the page and provide the final link to the Fast-SG repository.

Points raised by Reviewer #1:

Reviewer #1: Thank you for addressing my comments to satisfaction. Addition of "Procedure for effective hybrid assembly with FAST-SG" section would be very useful for the users.

Suggestion:
I think that Pie-chart representation of scaffolding errors (in previous version) appears neat as compared to current bar-chart. I only requested a minor change to convert the percentage values in pie chart to real numbers. Sometimes, percentage values in pie charts could be a misleading comparison because same percentage value in two pie chart could represent very different real

numbers.

Authors can decide which figure to include in the manuscript and I have no reservation with that decision.

A: We followed the Reviewer’s suggestion and we went back to the pie chart figure. We do thank again the Reviewer for this positive overview of our work.
