# Supplementary material for: Fast-SG: an alignment-free algorithm for hybrid assembly
Source: Gigascience. 2018 May 5;7(5):giy048. doi: 10.1093/gigascience/giy048 (PMC6007556; doi:10.1093/gigascience/giy048)
Supplement: Reviewer-1-Report-(Original-Submission) -- Sagar Utturkar — 21 Dec 2017 Reviewed [file giy048_reviewer-1-report-(original-submission).pdf]

Reviewer Report

Title: Fast-SG: An alignment-free algorithm for hybrid assembly

Version: Original Submission **Date:** 21 Dec 2017

Reviewer name: Sagar Utturkar

Reviewer Comments to Author:

Manuscript Summary:
Author's present a novel algorithm FAST-SG that can generate scaffolding graphs even with low (~5X) long-read coverage and produce synthetic libraries with long insert sizes. These can be combined using multiple legacy scaffolding tools and generate assembly results comparable to long-read assemblers (requiring high (~30x) coverage).

Review Summary:
The FAST-SG algorithm and results presented here are novel and valid. This algorithm would serve as a nice addition to current tools for generating high-quality assemblies with low long-read coverage data. However, I recommend a major revision in terms of structure and organization to make it more appealing to the readers. Also request authors to include some additional information.

Review comments:
As per my understanding, the main features of the FAST-SG are:
* Generating scaffolding graphs and synthetic mate-pair libraries which have library size up to BACs (180kb).
* This could be achieved with very low coverage of long-reads (5X) and provides a novel paradigm for hybrid assembly
* These FAST-SG libraries can be combined with any leading scaffolders to generate high-quality assemblies
* FAST-SG assembly results (with 5X long-read coverage) are as good as long-reads heavy (50x) assembly
* FAST-SG is compatible with bacterial as well as human genome and can be achieved with moderate computational power.

These are very interesting results. However, currently manuscript present these results in a somewhat imperative manner rather than stating those upfront. From the last paragraph in the background section and overall results section, this appears as a benchmarking study and presented in kind of report format. Author's should consider stating the novelty and important findings upfront, shorten the section on comparison with read-aligners and include a section on how to effectively use this algorithm (See below).

I see that FAST-SG is used in conjunction with other tools (DISCOVARDENOVO, LORDEC, SCAFFMATCH, BOSS, BESST2, OPERA-LG). It would be beneficial if authors could comment on:

* How to choose one scaffolder over other or any recommendations based on available data types/sequence coverage. Or it is suggested to try out multiple scaffolders and choose the best assembly by statistics?
* Are these synthetic libraries compatible with any scaffolding tools e.g. SSPACE?
* How to verify the quality of the generated synthetic libraries?
* Consider providing a flowchart for the FAST-SG workflow. Majority of the times, the audience of such manuscripts are biologists who want to use this tool with their data. Author's comments on usability of this tool would be very helpful to make informed decisions.

While comparing the scaffolded assemblies, what is the percentage of N's (uncalled bases) in each assembly? Better continuity may not always a good result if it has a very high percentage of uncalled bases. This will provide an additional estimate of how assembly compares with the 50x coverage data.

I appreciate the future directions provided in the conclusion section. Consider moving this to the "Results/Discussion" section. Conclusions should be summarizing the usability and novelty of this method.

Minor comments:

Figure 2. Consider using the thin vertical lines to show the average performance.

Figure 4. In the pie charts, provide the number of errors than percentages. This will allow more meaningful comparison.

Authors have mentioned 15% error rate for the PacBio data. This should be accompanied by the fact that "this high error rate could be overcome by providing sufficient sequence coverage (100x)".

Manuscript contains several complex sentences and incorrect use of grammar (e.g. have been instead of has been) and few typos (MySeq should be MiSeq) etc. Please review the manuscript in this aspect. Use of free tools like "Grammarly" may be helpful for rapid revision.

To summarize, this is a very nice and novel work. Above mentioned changes should help to make it more appealing to the wide audience.

Level of Interest

Please indicate how interesting you found the manuscript: An article whose findings are important to those with closely related research interests

Quality of Written English

Please indicate the quality of language in the manuscript: Needs some language corrections before being published

Declaration of Competing Interests

Please complete a declaration of competing interests, considering the following questions:

- Have you in the past five years received reimbursements, fees, funding, or salary from an organisation that may in any way gain or lose financially from the publication of this manuscript, either now or in the future?
- Do you hold any stocks or shares in an organisation that may in any way gain or lose financially from the publication of this manuscript, either now or in the future?
- Do you hold or are you currently applying for any patents relating to the content of the manuscript?
- Have you received reimbursements, fees, funding, or salary from an organization that holds or has applied for patents relating to the content of the manuscript?
- Do you have any other financial competing interests?
- Do you have any non-financial competing interests in relation to this paper?

If you can answer no to all of the above, write 'I declare that I have no competing interests' below. If your reply is yes to any, please give details below.

I declare that I have no competing interests

I agree to the open peer review policy of the journal. I understand that my name will be included on my report to the authors and, if the manuscript is accepted for publication, my named report including any attachments I upload will be posted on the website along with the authors' responses. I agree for my report to be made available under an Open Access Creative Commons CC-BY license (http://creativecommons.org/licenses/by/4.0/). I understand that any comments which I do not wish to be included in my named report can be included as confidential comments to the editors, which will not be published.

I agree to the open peer review policy of the journal

To further support our reviewers, we have joined with Publons, where you can gain additional credit to further highlight your hard work (see: https://publons.com/journal/530/gigascience). On publication of this paper, your review will be automatically added to Publons, you can then choose whether or not to claim your Publons credit. I understand this statement.

Yes
